# Benchmarking methods for mapping functional connectivity in the brain

**DOI:** 10.1038/s41592-025-02704-4

**Published:** 2025-06-06

**Authors:** Zhen-Qi Liu, Andrea I. Luppi, Justine Y. Hansen, Ye Ella Tian, Andrew Zalesky, B. T. Thomas Yeo, Ben D. Fulcher, Bratislav Misic

**Affiliations:** 1 Montréal Neurological Institute, McGill University, Montreal, Quebec Canada; 2 Melbourne Neuropsychiatric Centre, The University of Melbourne, Melbourne, Victoria Australia; 3 Yong Loo Lin School of Medicine, National University of Singapore, Singapore, Singapore; 4 School of Physics, The University of Sydney, Sydney, New South Wales Australia

**Keywords:** Computational neuroscience, Network topology, Statistical methods

## Abstract

The networked architecture of the brain promotes synchrony among neuronal populations. These communication patterns can be mapped using functional imaging, yielding functional connectivity (FC) networks. While most studies use Pearson’s correlations by default, numerous pairwise interaction statistics exist in the scientific literature. How does the organization of the FC matrix vary with the choice of pairwise statistic? Here we use a library of 239 pairwise statistics to benchmark canonical features of FC networks, including hub mapping, weight–distance trade-offs, structure–function coupling, correspondence with other neurophysiological networks, individual fingerprinting and brain–behavior prediction. We find substantial quantitative and qualitative variation across FC methods. Measures such as covariance, precision and distance display multiple desirable properties, including correspondence with structural connectivity and the capacity to differentiate individuals and predict individual differences in behavior. Our report highlights how FC mapping can be optimized by tailoring pairwise statistics to specific neurophysiological mechanisms and research questions.

## Abstract (web summary)

In this Analysis, Liu et al. benchmark more than 200 pairwise statistics for functional brain connectivity in tasks such as hub mapping, distance relationships, structure–function coupling and behavior prediction, revealing varying effectiveness for specific neurophysiological applications.

## Main

The brain is a network of anatomically connected and perpetually interacting neuronal populations^1^. Its spectrum of functions—from perception to cognition to action—depends on interregional signaling. Over the past 20 years, the dominant paradigm to infer interregional signaling has been to estimate functional connectivity (FC)^2–7^. Regional time series of metabolic, electromagnetic or hemodynamic neural activity are first recorded, and systematic coactivation between regions is then estimated and used to map FC networks^8–13^.

Perhaps the most widespread paradigm of estimating FC networks is the use of task-free or resting-state functional magnetic resonance imaging (fMRI)^12,14^. Neuronal population dynamics, in this setting, are recorded without task instruction or stimulation; the resulting ‘intrinsic’ FC is thought to reflect spontaneous neural activity. Intrinsic functional patterns are highly organized^15,16^, reproducible^17^, individual-specific^18,19^, correlated with structural connectivity^20,21^ and comparable to task-driven coactivation patterns^22,23^.

Unlike structural connectivity, which represents anatomical connections, FC is a statistical construct and does not represent a physical entity^12,24,25^. As a result, there is no straightforward ‘ground truth’, and how FC is estimated is a subjective methodological choice made by each individual researcher. Although multiple methods have been proposed, the most common method remains the simple zero-lag linear (Pearson’s) correlation coefficient. Yet, the broader scientific literature on estimating pairwise interactions among random variables is rich and vast, including those that capture, for example, nonlinear dependencies and time-lagged interactions^26–28^. A prominent example are methods based on inverse covariance, which attempt to model and remove the common network influences on two nodes in order to emphasize their direct relationships. Indeed, the brain mapping community increasingly calls for methods that are sensitive to multiple underlying mechanisms^29^. How FC matrices vary with the choice of pairwise statistic is a fundamental methodological question that affects all studies in this field, limiting our understanding of the brain’s functional organization, as well as our capacity to develop optimized algorithms for structure–function coupling, individual fingerprinting and brain–behavior prediction^3,9,10,14,30–40^.

Here, we comprehensively benchmark multiple features of resting-state FC using 239 pairwise interaction statistics. We first chart the similarities and differences among broad families of statistics. We then investigate how commonly studied features of the FC matrix—such as hubs, relationships with physical distance and structural connectivity—vary with the choice of pairwise statistic. We next show that individual differences in FC organization, including fingerprinting and brain–behavior relationships, also depend on the choice of pairwise statistic. Finally, we use an information-theoretic decomposition to study how pairwise statistics capture different mechanisms of information flow.

## Results

Pairwise statistics were derived for *N* = 326 unrelated healthy young adults from the Human Connectome Project (HCP)^41^. Functional time series were taken from the HCP S1200 release. We used the pyspi package to estimate 239 pairwise statistics from 49 pairwise interaction measures in 6 families of statistics, yielding 239 FC matrices^26^ for each participant. All main text results are shown for the undirected component of the matrices (upper triangular vector), and in the Schaefer 100 × 7 atlas. For other atlases and alternative processing choices, see the ‘Sensitivity analyses’ section in the Methods.

### Massive profiling of pairwise interaction statistics

**Fig. 1:**
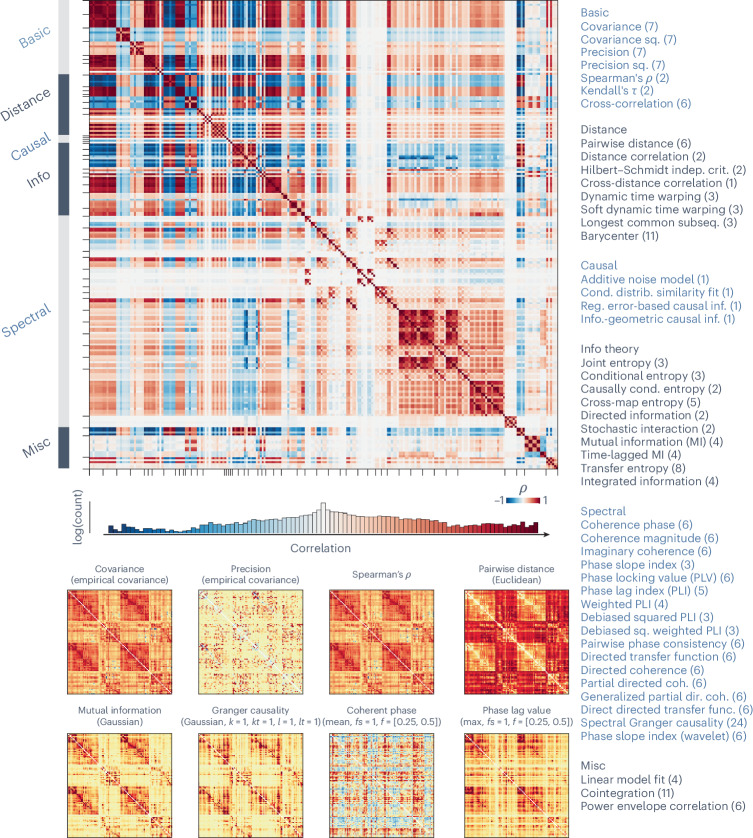
Massive profiling of pairwise interaction statistics for resting-state functional activity across the brain. Pairwise statistics for functional time series were estimated between all pairs of brain regions to generate 239 distinct FC matrices. Top left: group-average similarity between all pairs of 239 pairwise statistics. Edge-wise similarities between individual pairwise statistics were quantified using Spearman’s rank correlation (*ρ*) for each participant and then averaged across participants. The histogram of similarity values is shown below the matrix. The color represents [−1, 1], and the bar height represents log-transformed count in each bin within the range of [−1, 1]. Bottom left: group-average matrices for exemplar statistics calculated between pairs of time series. The annotation above each matrix denotes the broader family of the statistic and (in parentheses) details for the specific statistic. Right: a list of 239 pairwise statistics grouped into 49 measures across 6 major model families, following the categorization of ref. ^26^. Numbers in parentheses indicate the number of specific variants of the statistics calculated for the measure. The color bar covers only positive values (0th to 97.5th percentile, in red) for statistics with only positive values, and covers both negative values (0th percentile to zero, in blue) and positive values (zero to 97.5th percentile, in red) otherwise. A detailed list of the 239 pairwise statistics can be found in Supplementary Table 1. The variance of the similarity matrix across participants and runs can be found in Supplementary Fig. 1. Info, information; Misc, miscellaneous; sq., squared; indep., independence; crit., criterion; subseq., subsequence; reg., regression; info., information; cond., conditional; distrib., distribution; MI, mutual information; coh., coherence; dir., directed; func., function. *k*, *kt*, *l*, *lt*, *fs* and *f* are parameters for specific statistics.

We first show edge-wise similarities between the 239 FC matrices (Fig. 1, top). Pairwise statistics are stratified according to the broad model family from which they are derived (for example, information theoretic, spectral and so on). The 49 pairwise measures are listed on the right, as well as the number of variants of each measure, which we refer to as pairwise statistics (239 total)^26^.

Pairwise statistics are highly organized and form clusters that reflect families of statistics. For reference, the conventional zero-lag Pearson’s correlation is shown as the covariance family, and partial correlation is shown in the precision family in all figures. Some statistics are, by definition, highly similar to others. The most widely used family of statistics for FC calculation, covariance estimators, for example, are most correlated with correlation, distance correlation and mutual information estimators. As expected, these measures of similarity tend to be highly anticorrelated with measures of dissimilarity such as precision, distance and entropy. Others—for example, spectral measures—show mild-to-moderate correlation with most other measures. Importantly, the correlations among the pairwise statistics distribute widely across the positive to negative range. For example, eight sample FC matrices are shown in Fig. 1 (bottom), with clear differences in organization, such as the extent to which they display block-like structure. Collectively, this suggests that different methods used to compute the FC matrix may yield networks with very different configurations.

### Benchmarking topological and geometric organization

**Fig. 2:**
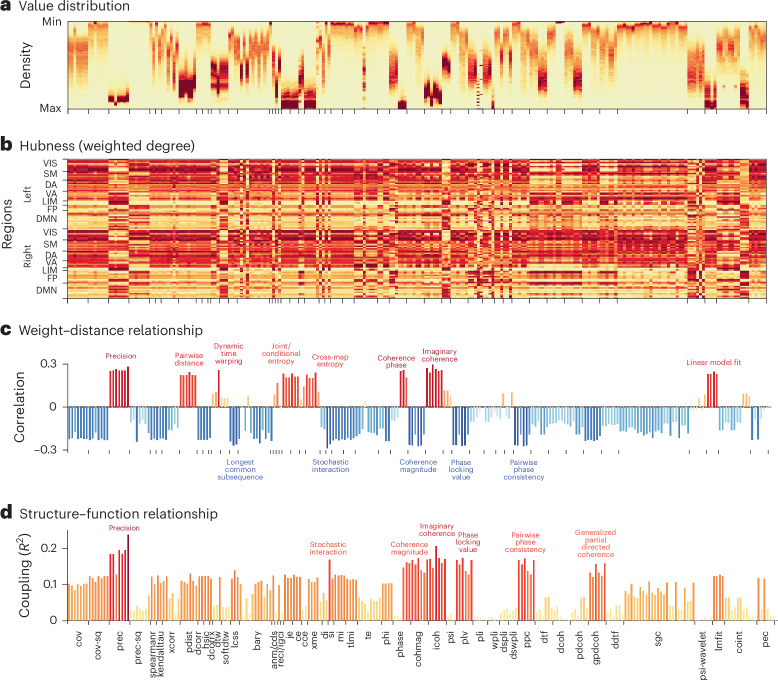
Benchmarking topological and geometric organization. **a**, Value distribution for each interaction statistic. Values were min–max-normalized within each statistic. Darker red denotes greater density. **b**, Ranking of hubs quantified by weighted degree (strength) of the pairwise statistic matrices. Absolute values are taken from the pairwise statistics before ranking. Note that pairwise statistics with positive correlations with spatial distance (shown in **c**) have flipped rankings to ensure a more consistent hub representation. Regions are ordered by intrinsic functional networks from ref. ^15^ for left and right hemispheres. Darker red means greater weighted degree (‘hubness’). VIS, visual; SM, somatomotor; DA, dorsal attention; VA, ventral attention; LIM, limbic; FP, frontoparietal; DMN, default mode network. The organization of hubs when considering positive and negative values separately can be found in Supplementary Fig. 2. The similarity of hub organization across pairwise statistics and their representation on the cortex are shown in Supplementary Fig. 3. **c**, The weight–distance relationship quantified by computing the Spearman’s rank correlation of each edge in each pairwise statistic matrix with interregional Euclidean distance (physical distance between brain regions). Colors and bar height represent the magnitude of correlation. The most extreme measures are labeled with text. **d**, Structure–function coupling between matrices of interaction statistics and predictor matrices derived from structural connectivity. Structure–function coupling is represented using the coefficient of determination (adjusted *R*^2^), such that low values indicate poor structure–function coupling and high values indicate strong structure–function coupling^46,47^. Colors and bar height represent the magnitude of coupling. The most extreme measures are labeled with text. Full names for the statistics can be found in Supplementary Table 1.

If pairwise statistics yield FC matrices that look different, do these matrices also have different topological and geometric features? We start by inspecting the probability density of edge weights for each matrix (Fig. 2a; each column represents a pairwise interaction statistic, following the order in Fig. 1). Some densities are highly skewed while others are more evenly distributed, suggesting differences in topological organization, such as the presence or nonpresence of hubs, respectively. We next consider the weighted degree of every brain region in each of the FC matrices (brain regions × pairwise statistics; Fig. 2b). Although there exist some patterns that are common to most pairwise statistics (for example, high weighted degrees in dorsal attention, ventral attention, visual and somatomotor networks), there is also considerable variability across pairwise statistics. For instance, some families of statistics tend to have more spatially distributed hubs, additionally emphasizing transmodal regions, such as precision-based pairwise statistics that detect prominent hubs in default and frontoparietal networks (Fig. 2b).

Next, we quantify to what extent each of the pairwise statistics recapitulates two well-studied features of brain networks: (1) the inverse relationship between physical proximity and edge weight^21,42–45^, and (2) the positive relationship between structural connectivity and FC^20,21,45,46^. For each pairwise statistic, we compute the correlation between the interregional Euclidean distance and the magnitude of FC (Fig. 2c). Note that some pairwise statistics are defined as the distance (dissimilarity) between time series (for example, precision, pairwise distance and linear model fit); in those cases, greater values indicate dissimilar time series, and we expect to see a positive correlation between physical distance and FC. Overall, most pairwise statistics display a moderate inverse relationship between physical proximity and pairwise association (0.2 < ∣*r*∣ < 0.3), although several display a weaker relationship (∣*r*∣ < 0.1). This finding illustrates how even a fundamental feature of brain networks that has been reported across imaging and tracing techniques, spatial scales, and species can vary substantially depending on how FC is defined. This suggests that pairwise statistics are differentially sensitive to different types of underlying mechanisms, a question we explore in greater detail in the ‘Decomposing FC matrices into information flow patterns’ section.

For each pairwise statistic matrix, we evaluate the goodness of fit between diffusion MRI-estimated structural connectivity and the magnitude of FC (Fig. 2d). Here, we expect a positive relationship, reflecting the fact that axonal projections support interregional signaling and the emergence of coherent dynamics among neuronal populations^21^. Again, we observe substantial variability across pairwise statistics, with structure–function coupling ranging from 0 to 0.25 (measured by *R*^2^). Pairwise statistics with the greatest structure–function coupling include precision, stochastic interaction and imaginary coherence. These results parallel the findings above in two ways. First, they show gross compatibility but also substantial variability for an observation that has been reported in multiple studies. Second, we observe the strongest ‘expected’ results (inverse relationship with distance and positive relationship with structural connectivity) for commonly used covariance-based pairwise statistics and for some others, such as precision-based pairwise statistics. These statistics may be well suited for optimizing structure–function coupling because they seek to partial out or account for shared influence among multiple regions, emphasizing functional interactions that arise from structural connections (Discussion).

### Alignment with multimodal neurophysiological networks

**Fig. 3:**
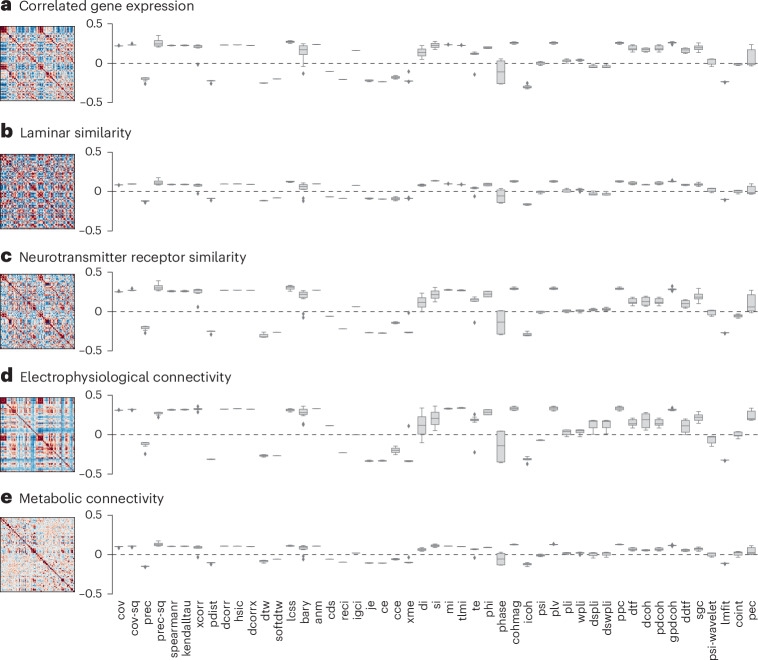
Alignment with multimodal neurophysiological networks. **a**, Interregional similarity networks (left) correlated with interaction statistics matrices (right) for correlated gene expression derived from Allen Human Brain Atlas microarray data^88^. **b**, Laminar similarity derived from BigBrain histological intensity profile segmented into cortical layers^89,90^. **c**, Neurotransmitter receptor similarity derived from PET tracer images of 18 neurotransmitter receptors and transporters^49^. **d**, Electrophysiological connectivity derived from resting-state MEG^47,48,84^. **e**, Metabolic connectivity derived from correlating FDG-PET time-resolved activity^84,91^. The *y* axis represents the Spearman’s rank correlation between interregional similarity networks and interaction statistics matrices. The box plots show the median as the center line, upper and lower quantiles as box limits, 1.5× interquartile range as whiskers and outliers as points. The mean and variance of the alignment across the five neurophysiological networks are shown in Supplementary Fig. 5.

The previous section demonstrates that even basic relationships with geometry and anatomical connectivity can vary substantially depending on how FC is estimated. Here, we extend this question and consider how different types of FC correspond to other networks that reflect biological similarity between brain regions. Specifically, we estimate multiple forms of interregional similarity, including correlated gene expression (Fig. 3a, derived from the Allen Human Brain Atlas microarray data), laminar similarity (Fig. 3b, derived from the Merker-stained BigBrain Atlas), neurotransmitter receptor similarity (Fig. 3c, derived from multiple positron emission tomography (PET) tracers), electrophysiological connectivity (Fig. 3d, derived from magnetoencephalography (MEG)) and metabolic connectivity (Fig. 3e, derived from dynamic [^18^F]-fluorodeoxyglucose (FDG)-PET). For a complete description of how each matrix is constructed, see the Methods. Our main question here is how well each FC matrix aligns with interregional biological relationships estimated at different spatial and temporal scales.

We show the correlation between each FC matrix and each biological interregional similarity matrix in Fig. 3. We observe the strongest correspondence with neurotransmitter receptor similarity and electrophysiological connectivity. This is consistent with the previous literature and potentially reflects the fact that regions with similar chemoarchitectural profiles are subject to common neuromodulatory influences, leading to coherent electrophysiological dynamics^47–49^. We find similar results when we estimate the alignment between pairwise interaction statistics matrices and a ‘cognitive similarity’ matrix that indexes how areas coactivate across cognitive tasks (derived from the Neurosynth meta-analytic engine) (Supplementary Fig. 6). Perhaps counterintuitively, we do not observe strong correspondence between fMRI-estimated FC and FDG–PET-estimated metabolic connectivity, despite the fact that the two methods should theoretically be measuring related biological processes. Finally, in what is a recurring theme, FC estimated using precision-based statistics generally continue to be closely aligned with multiple biological similarity networks.

### Quantifying individual differences

**Fig. 4:**
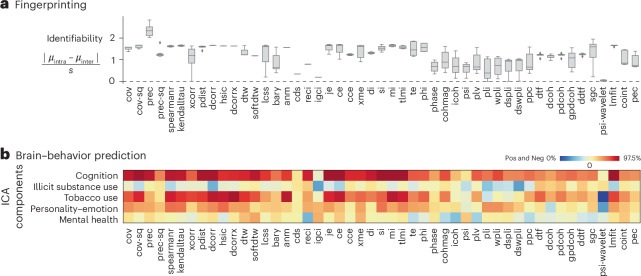
Quantifying individual differences. **a**, Individual fingerprinting quantified using the identifiability index^51,54^, estimated from the four fMRI runs for each participant. The box plot shows the median as the center line, upper and lower quantiles as box limits, 1.5× interquartile range as whiskers and outliers as points. **b**, Brain–behavior prediction using pairwise statistics as predictors and five ICA-derived cognitive-behavioral components as the outcome^55^. Kernel ridge regression is performed under a nested tenfold cross-validation setting. The heatmap colors display the mean Pearson’s correlation between the empirical and predicted behavior scores across the test folds. The colorbar covers both negative values (0th percentile to zero, in blue) and positive values (zero to 97.5th percentile, in red). Sensitivity analyses, using alternative machine learning algorithms (kernel ridge regression with a cosine kernel, linear ridge regression and LASSO regression) are shown in Supplementary Figs. 8–10. Pos, positive; Neg, negative.

A common application of resting-state FC is to study individual differences^50^. Here, we examine how FC estimated using different pairwise statistics can be used for (1) identifying individuals (fingerprinting)^18,51^ and (2) predicting individual differences in cognition and behavior^52,53^. We show participant identifiability for FC matrices computed using different pairwise statistics in Fig. 4a. The identifiability index is a measure of effect size, where a magnitude of ≥0.8 is considered large^54^. In brief, identifiability measures how similar an individual is to themselves across multiple scans, compared with other individuals^51,54^. Consistent with previous reports, we find that covariance measures (for example, Pearson’s correlation) generally perform well (identifiability ~1.5)^54^. Precision-based statistics outperform all others (identifiability >2.1), mirroring the results in the previous section. The broad question of whether FC organization persists across participants and scans is sometimes alternatively formulated as test–retest reliability. For completeness, we also perform a test–retest analysis using intraclass correlation, yielding results consistent with previous literature^17^ and similar to fingerprinting identifiability (Supplementary Fig. 7). In particular, precision-based statistics have relatively low test–retest reliability, suggesting its targeted ability toward capturing more of individual differences than similarities.

We next consider how well different FC pairwise statistics can be used for out-of-sample prediction of individual differences in cognition and behavior. Following the approach outlined by Tian and colleagues^55^, we apply independent component analysis (ICA) to 109 measures in the HCP dataset to derive a five-component solution. The components broadly capture individual differences in cognition, illicit substance use, tobacco use, personality–emotion and mental health^55^. We then use kernel ridge regression in a nested tenfold cross-validation setting to predict individual component scores from individual FC matrices^52,56^. The resulting mean correlation between empirical and predicted scores across the test folds is shown in Fig. 4b. We generally observe greater prediction for cognition and tobacco use, and poor prediction for illicit substance use and mental health, consistent with previous reports^54,55,57^. Pairwise statistics that perform well for individual fingerprinting (for example, covariance, precision and information theory-based statistics) also tend to perform well for predicting cognition and behavior; likewise, pairwise statistics that perform poorly for fingerprinting also perform poorly here (for example, spectral statistics). Collectively, the substantial variation in identifiability and prediction accuracy suggests that the choice of pairwise statistic for computing FC is an important one that could be tailored or optimized for different research questions.

### Decomposing FC matrices into information flow patterns

**Fig. 5:**
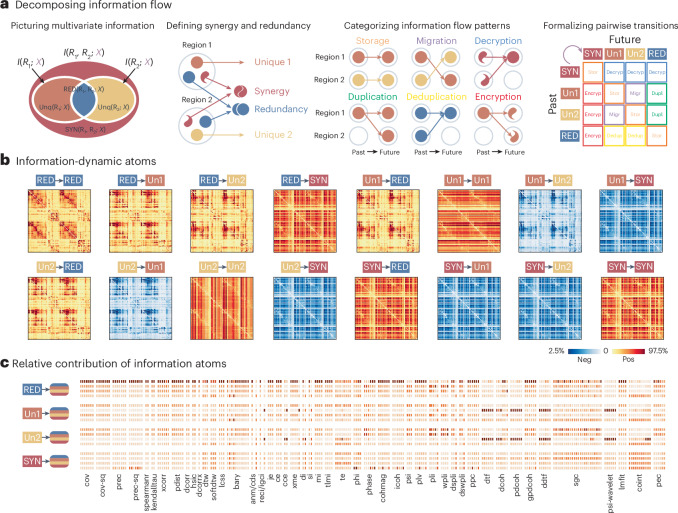
Decomposing FC matrices into information flow patterns. **a**, A schematic of the integrated information decomposition framework (ΦID), showing how pairwise information is estimated and decomposed into basic motifs of information flow^59–61^. **b**, The framework yields 16 distinct information-dynamic atoms that represent different patterns of information flow. **c**, The relative contribution of the 16 information-dynamic atoms to each pairwise interaction statistic, quantified as relative importance estimated using dominance analysis. In each column, darker red shows greater contribution from a specific information-dynamic atom. The 16 rows, grouped by the ‘past’ state, correspond to the 16 information-dynamic atoms in the same order as **b**.

Up to now, we focused on associating FC matrices with other types of interregional relationship (for example, structural connectivity, spatial proximity and interregional biological similarity) and with exogenous measures (for example, individual identity or behavior). Here, we ask whether FC computed using different pairwise statistics reflects different underlying patterns of information flow. We estimate, for instance, ‘synergistic’ interactions where two sources of information, when considered together, provide new information that cannot be retrieved from either source individually, and by contrast, ‘redundant’ interactions where the opposite is true, and each source provides the same information as the other. A recent information-theoretic framework makes it possible to partition pairwise interactions into synergistic, redundant and unique information, also known as information atoms^58–61^ (Fig. 5a). In brief, for each pair of cortical regions (treated as sources), we can ask how much information about their future neural activity can be obtained from knowing their past activity—and whether this information is carried redundantly by each of them separately, or uniquely by one of them or synergistically by both together. We can then also ask if the way that information is carried changes over time, giving rise to different types of information dynamics. For example, if information was initially provided uniquely by region A, and then it is provided uniquely by region B, this is a case of information transfer from A to B.

We show the 16 information flow patterns arising from this information decomposition in Fig. 5b ref. ^61^. We then estimate the contribution of each of the 16 information flow patterns to each FC matrix (Fig. 5c). We find that classic statistics, such as covariance, precision and mutual information, mostly reflect the pattern whereby redundant information stays redundant. Some spectral statistics, such as directed transfer function and partial coherence, predominantly reflect a pattern where information that is provided exclusively by one region stays unique to that region. While both of the cases above belong to a pattern of information storage, whereby information is consistently conveyed in the same way over time, a greater diversity of information flow patterns exists. For example, we observe the presence of information migration, duplication and deduplication in phase lag value. We also observe information encryption and decryption (also known as downward and upward causation^61^) in transfer entropy and cointegration. Altogether, these results show that, while most statistics capture redundant information storage, there exists a wider landscape of information flow patterns that can potentially be selectively sampled using specific pairwise statistics.

### Summary rankings, sensitivity analyses and validation

To summarize the benchmarking findings so far, we compile the rankings of pairwise statistics according to six criteria: (1) negative weight–distance relationship, (2) positive structure–function coupling, (3) close correspondence with biological similarity networks, (4) high individual–participant identifiability, (5) high brain–behavior prediction and (6) low susceptibility to participant motion (Supplementary Table 5 and Supplementary Fig. 16). Broadly, inverse covariance measures tend to have the greatest composite ranking, but the individual rankings vary considerably between criteria. Collectively, these results suggest that there is not necessarily a single optimal pairwise statistic, but rather different options that can be used to target desired mechanisms.

**Fig. 6:**
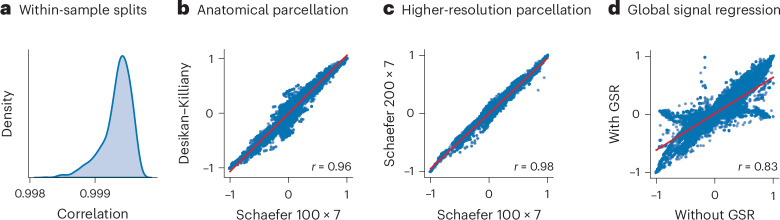
Sensitivity analyses. The group average similarity profile of the pairwise statistics in Fig. 1 is reproduced under different processing conditions for comparison. **a**, The distribution of correlations between split-half discovery and validation sets for 1,000 random splits. **b**, The correlation between the functionally derived atlas (Schaefer 100-node 7-network) used in the main analyses and an anatomically derived atlas (Desikan–Killiany). **c**, The correlation between the 100-node atlas (Schaefer 100-node 7-network, Schaefer 100 × 7) used in the main analyses and a higher-resolution atlas (Schaefer 200-node 7-network, Schaefer 200 × 7). A subset of 179 interaction statistics were used in this panel for faster calculation and can be found in Supplementary Table 2. **d**, The correlation between functional time series without global signal removal used in the main analyses and with global signal removal. GSR, global signal regression. The dimensions of the similarity matrices are the same as the number of pairwise interaction statistics (239), except for **c**, where a reduced set of measures (179) was used. Additional analyses regarding the effect of participant motion can be found in Supplementary Fig. 11. Spearman’s rank correlation is used in all panels.

We next seek to determine the extent to which the main results are sensitive to the several processing and data-handling choices that exist in resting-state fMRI network modeling. We first test the stability of the group-level similarity matrix (originally shown in Fig. 1). We perform 1,000 random splits of the sample into discovery and replication sets and compute the correlation between them. The distribution of correlation coefficients is centered above *r* = 0.999 (Fig. 6), suggesting close concordance. To test the effect of atlas, we compute the similarity between matrices generated using a functional parcellation (Schaefer 100 × 7) and an anatomical parcellation (Desikan–Killiany), revealing a correlation of *r* = 0.96. To test the effect of atlas resolution, we compute the similarity between matrices generated using a lower-resolution atlas (Schaefer 100 × 7) and a higher-resolution atlas (Schaefer 200 × 7), revealing a correlation of *r* = 0.98. To test the effect of global signal regression, we compute the similarity between matrices generated with and without global signal regression, revealing a correlation of *r* = 0.82. Similar results were observed when repeating all sensitivity analyses at the individual participant level (Supplementary Figs. 13 and 14).

Finally, we ask whether the present results generalize to other datasets and acquisitions. We apply the same analytic procedure to six additional fMRI datasets that including a wide range of acquisitions (single- and multiband, as well as a range of voxel sizes, repetition time and scan durations) and preprocessing pipelines: (1) HCP–retest (*N* = 20), (2) AOMIC–PIOP1 (*N* = 216), (3) AOMIC–PIOP2 (*N* = 226), (4) MSC (*N* = 10), (5) MPI–MBB (*N* = 126) and (6) RBC–NKI (*N* = 592) (see the Methods for more details). Supplementary Fig. 17 shows a conserved similarity structure of pairwise statistics across datasets, while Supplementary Fig. 18 shows a conserved similarity structure of pairwise statistics for a single dataset using three different acquisition protocols and two different motion correction methods. Finally, Supplementary Figs. 19 and 20 show the stability of each individual pairwise statistic across the datasets. Collectively, these sensitivity checks suggest that the global relationships among pairwise statistics are relatively stable with respect to multiple methodological choices.

## Discussion

Resting-state FC is rapidly becoming one of the most widely used brain imaging phenotypes. Despite its popularity, the operational definition of FC is arbitrary, and most groups use simple zero-lag linear correlations by default. In the present report, we benchmark the network architecture, biological underpinnings and brain–behavior associations of FC matrices computed using a large library of pairwise interaction statistics. Our results reveal a rich landscape of methods that are sensitive to different features of brain organization.

Even for well-studied phenomena, we observe substantial variability across methods. The arrangement of highly connected hub regions, a topic of great interest over the past 10–15 years^62–64^, systematically varies depending on the method, with some localizing hubs in unimodal cortex and others more widespread across the unimodal–transmodal axis. The weight–distance relationship, reported not only for FC-fMRI but also for diffusion MRI^44,65^, and tract tracing in multiple species^66^, is captured by most methods, but the magnitude of the effect varies considerably. Finally, a similar result is observed for structure–function coupling, whereby most methods identify an overall positive relationship, but the effect size displays variability across methods. In other words, the choice of pairwise interaction statistics has substantial influence on the spatial and topological organization of reconstructed functional networks.

One reason for the observed variability is that pairwise statistics are sensitive to different underlying mechanisms of interregional signaling^67^. We find that different FC methods often align with different forms of interregional biological similarity, from microscale correlated gene expression or receptor similarity, to macroscale electrophysiological coupling. Indeed, numerous reports have found evidence of association between resting-state BOLD FC and correlated gene expression^68^, receptor similarity^49^ and electrophysiological rhythms^47,48,69^. Indeed, the different pairwise statistics are optimized to capture different types of communication process^70^. Resting-state functional dynamics are thought to be mostly macroscopically linear^71^, and as a result, many conventional FC methods are designed to capture linear effects. However, the complexity of functional dynamics extends beyond simple linear effects, and a broader set of pairwise statistics is necessary to completely capture the rich spectrum of interactions in fMRI BOLD neural dynamics^26,28,40^.

Ultimately, one of the main reasons why neuroscientists study statistical relationships between regional BOLD time series is the belief that brain regions exchange, store and process information and that this information can be reflected by statistical relationships. However, there is a growing understanding that information can be transmitted, processed and stored in different ways—raising the question of how each pairwise statistic captures (or fails to capture) these different kinds of information dynamics. To directly address this question, we applied information decomposition and found that different FC methods align with different forms of information dynamics. Most FC methods appear to capture storage of redundant information, whereby both regions convey the same information—as previously observed for Pearson correlation^59,72^. However, some measures are sensitive to other forms of communication, including synergistic and unique information flow. These results demonstrate a multitude of communication patterns between brain regions that are explored less often but that should be taken into account for a more comprehensive mapping of the functional connectome and more nuanced inferences about what FC represents^25,73^.

Across the benchmark criteria tested, there is not necessarily a single optimal pairwise statistic. In this sense, our results can be seen as a rough guide for matching a pairwise statistic to an experimental question. A salient example is how the choice of FC method is context dependent in individual differences and brain–behavior relationships, where we find that the predictive utility of a FC method depends on the phenotype that one seeks to predict^54,55,57^. More broadly, our results highlight the idea that, in the absence of any ground truth, picking a pairwise statistic is an important question that strongly depends on the research question at hand^25,29,73–75^.

What recommendations can be derived from the present findings? Although we sampled a limited set of possible analyses, some broad arcs come into focus. First, as discussed above, a pairwise statistic should be matched to the experimental question. Second, covariance (distance)-based methods appear to have many desirable properties, including robust relationships with physical proximity, structural connectivity and biological interregional similarity, as well as the capacity to differentiate individuals and predict individual differences in multiple phenotypes. Methods based on precision (inverse covariance or partial correlation) stand out. Indeed, these measures have often been touted as the superior alternative to the Pearson’s correlation for estimating FC^4,76–79^. By removing mutual dependencies on common influences from other areas, precision has the theoretical advantage of more directly measuring directed, anatomically mediated interactions among brain areas^9,29,33,37,38,80–83^. An exciting future avenue would be to combine multiple FC matrices to engineer new types of FC that are potentially sensitive to a wider range of desirable properties^84^.

It is also important to consider whether the assumptions inherent in a pairwise statistic match the acquisition and processing of a dataset. Procedures that alter the temporal sequence of frames, such as censoring or concatenation, can be problematic because they result in an irregular sampling rate in a time series and violate basic assumptions of many frequency-based measures (for example, phase synchrony) but do not affect measures that tolerate temporal exchangeability (for example, Pearson’s correlation). In this sense, processing strategies that do not remove entire frames (for example, ICA-FIX) may potentially circumvent this problem. In a similar vein, acquisitions that use faster sampling rates (for example, multiband) theoretically allow more optimal deployment of some pairwise statistics, such as those based on phase relationships.

Finally, the present results should be interpreted in light of multiple methodological limitations. First, we considered only undirected components of pairwise statistics, effectively ignoring directed or causal mechanisms^29,35^. Second, the main text analyses are based on the well-studied HCP dataset which involves a specially designed acquisition sequence and processing pipeline. To ensure generalizability, we repeated all benchmarks for six additional datasets but more work is needed to understand how acquisition affects the reconstruction of functional connectomes. Third, although we ensured robustness to common preprocessing choices such as parcellation type and size, removal of the global signal and alternative motion correction methods, we did not exhaustively consider the effects of all processing choices^36,74,85^. Fourth, we did not exhaustively consider all common research questions, such as the lifespan trajectory of FC or the effects of psychiatric and neurological disease on FC^86^. Fifth, we focused only on descriptive pairwise interaction statistics and did not explicitly consider model-based ‘effective connectivity’ methods, such as structural equation modeling or path analysis, dynamic causal modeling or biophysical neural mass modeling^5,8,87^.

In summary, the present report comprehensively benchmarks the architecture of resting-state BOLD FC using a large library of pairwise statistics. We observe substantial variation across FC methods and across a wide array of analyses, reflecting differential sensitivity to biological features and to types of information flow. As FC continues to grow in popularity as a neuroimaging phenotype, our results provide the foundation for future studies to tailor their choice of FC method to the neurophysiological mechanism they are targeting and to their research question.

## Methods

### Resting-state functional MRI

Resting-state functional time series from 326 unrelated participants were obtained from the HCP Young Adults cohort (HCP-YA; S1200 release^92^). Structural and functional MRI data were preprocessed using HCP minimal preprocessing pipelines^41,93^. High-resolution T1-weighted and T2-weighted structural images were corrected for gradient distortion and registered to the MNI152 atlas. Cortical surfaces were constructed using the FreeSurfer recon-all procedure. Resting-state BOLD functional images (four scans approximately 15 min long for each participant) were corrected for slice timing, gradient distortion, motion, echo planar imaging (EPI) distortion and registered to the high-resolution T1-weighted structural image, which further underwent intensity normalization and bias removal. The surface representations were then created by mapping the volumetric BOLD signal to the fsLR grayordinate space using MSMAll, a multimodal surface-based functional alignment algorithm^94^. Physiological noise and confounds were removed with the ICA-FIX procedure^95^. Details of the preprocessing steps can be found in the original technical reports^93^.

### Calculating pairwise interactions with pyspi

We used the recently developed Python Toolkit of Statistics for Pairwise Interactions (pyspi; v0.4.1, commit c19d06) to calculate the alternative measures (statistics of pairwise interactions; SPIs) of FC^26^. Resting-state fMRI time series derived in the previous step were parcellated using the Schaefer 100-node 7-network atlas^96^ and normalized (*z*-scored along the time dimension) before pyspi calculation. Starting with the original list of SPIs, we derived a subset of SPIs with a reasonable calculation time (<30 min) for a single participant and calculated the SPIs for all individual participants and resting-state runs. After aggregating the results, we further excluded the SPIs with (1) zero variance or (2) infinity or NaN (not a number) values for at least 1/4 of all participants and runs, finally obtaining 239 SPIs from 49 pairwise interaction measures across 6 major categories (see Supplementary Table 1 for the full list of SPIs used).

The calculation resulted in 239 node-by-node matrices for each participant and run. A group consensus matrix was calculated for each statistic by taking the average across all participants and runs (shown in Fig. 1; see Supplementary Fig. 1 for variance). A total of 239 group consensus matrices were generated, which we refer to as group-averaged measure matrices. We also calculated the similarity of the statistics by taking the Spearman’s rank correlation between pairs of statistic matrices for each participant and run, which we refer to as similarity profile matrices. A group consensus similarity profile matrix was calculated by taking the average across participants and runs.

Unless otherwise noted, we used the upper triangular values for the analyses (see Supplementary Fig. 12 for a brief account of directed pairwise statistics) and using Spearman’s rank correlation coefficient to assess the relationships between SPIs and other measures.

### Structure–function relationship

#### Structural network reconstruction

Structural network of the cohort was reconstructed from diffusion MRI tractography. Diffusion MRI scans were processed using the MRtrix3 package^97^). Fiber orientation distributions were modeled using multishell multitissue constrained spherical deconvolution algorithm^98,99^. White-matter streamlines were then reconstructed^100^ and optimized^101^ to provide robust estimate of tract weights. We estimated a binary group consensus structural connectivity matrix using a distant-dependent algorithm that approximates the group-level average edge length distribution^102^. The final weighted group consensus matrix was then calculated by applying the binary matrix on the simple average of structural connectivity matrices of all participants.

#### Structure–function coupling estimation

Following previous practices of quantifying structure–function relationships^46,47^, we used a multilinear regression model with network communication predictors to quantify the correspondence between structural and functional networks. This approach takes into account of potential dynamics processes happening on the network and provides a multi-faceted view of structure–function correspondence than using the structural connectivity alone. We adopted Euclidean distance and five commonly used network communication measures derived from the group consensus structural connectivity matrix as predictors. They represent a spectrum of routing strategies ranging from centralized, globally optimized shortest path to decentralized, locally focused diffusion^103–106^. We estimated the goodness of fit, adjusted *R*^2^ to quantify the extent of structure–function coupling in this case.

$${{\bf{W}}}_{{\mathrm{FC}}}={\beta }_{0}+\sum _{k}{\beta }_{k}{{\bf{W}}}_{k},$$

where **W**_FC_ denotes the pairwise interaction measures and **W**_*k*_ denotes predictor matrices: Euclidean distance, shortest path length, navigation efficiency, search information, communicability and diffusion efficiency.

We also calculated a more direct version of structure–function coupling using Spearman’s rank correlation between nonzero elements of the structural connectivity and the pairwise interaction statistic matrices (Supplementary Fig. 4)^107^.

#### Network communication measures

We used the Euclidean distance between region centroids as the physical distance between nodes. We also derived a connection length matrix *L* from the structural connectivity matrix when it came to quantifying the cost of traversing the edges. We used a monotonic weight-to-length transform in the form of $L=-\log \frac{{\bf{W}}}{{{\bf{W}}}_{{\mathrm{max}}}+1}$. The resulting connection length matrix (*L*) will have infinity values between a pair of regions that do not have a direct structural connection.

The shortest path length represents the shortest distance to travel from a source and a target node^108^. We calculated the shortest path lengths using the Floyd–Warshall algorithm^109^ with the connection length matrix *L*.

Network navigation was introduced to brain networks by Seguin and colleges^110–113^, quantifying routing without global optimization by simulating a walker that steps toward the neighbor node that is closest in distance to the target node. Here, we used Euclidean distance as the distance metric, and navigation efficiency is calculated as the inverse of the navigation path length.

Search information measures the amount of information necessary for a random walker on the network to travel along a specific path and does not take detours. The measures were adapted to the brain networks and the shortest path on weighted network in refs. ^113–116^.

Communicability measures the number of possible routes between a source and a target node pair. It is defined as the weighted sum of all paths and walks between those nodes^117,118^.

Diffusion efficiency is calculated as the inverse of the mean first passage time, which quantifies the time (number of steps) expected for a random walker to travel from a source to a target node. For asymmetric measures, we symmetrized the matrix by taking the average of the matrix with its transpose^114,115,119^.

The network measures were implemented using the Brain Connectivity Toolbox^120^ (https://sites.google.com/site/bctnet, version 2019-03-03), Brainconn (https://github.com/FIU-Neuro/brainconn, master branch at commit 8cd436) and netneurotools (https://github.com/netneurolab/netneurotools, v0.2.3).

### Biological networks

We adopted annotated networks from multiple modalities to contextualize the functional relationships. Here, we briefly describe how we acquire the networks. More technical details can be found in the previous reports^84^.

Electrophysiology connectivity was derived from resting-state MEG recordings^121^. Resting-state MEG data (approximately 6 min for each participant) for *N* = 33 healthy unrelated participants were taken from HCP. Preprocessing was carried out using open-source Brainstorm software (https://neuroimage.usc.edu/brainstorm/ (ref. ^122^)). In brief, raw MEG recordings were registered to high-resolution anatomical space before being submitted to notch filtering (60, 120, 180, 240 and 300 Hz), high-pass filtering (0.3 Hz), band channel removal and automatic artifact removal. Artifacts including heartbeats from electrocardiogram, eye blinks from electrooculogram, saccades, muscle movements as low-frequency (1–7 Hz) and high-frequency (40–240 Hz) components and noisy segments were removed using signal-space projections. Sensor-level data were then submitted to source estimation using a linearly constrained minimum variance beamformer on the HCP fsLR4K surface. The ‘median eigenvalue’ method from Brainstorm was used to reduce the variable source depth effect. Time series on fsLR4k surface were parcellated to the Schaefer 100 × 7 atlas using the first principal component of the corresponding vertices. MEG FC matrices were estimated using amplitude envelope correlation^123^ for the six canonical frequency bands: delta (*δ*; 2–4 Hz), theta (*θ*; 5–7 Hz), alpha (*α*; 8–12 Hz), beta (*β*; 15–29 Hz), low gamma (lo-*γ*; 30–59 Hz) and high gamma (hi-*γ*; 60–90 Hz). The spatial leakage effect was corrected using an orthogonalization process^124^. The final electrophysiology connectivity matrix used in this project is derived as the first principal component of the connectivity matrices for the six canonical bands. Details of preprocessing can be found in refs. ^48,84^.

The correlated gene expression network quantifies the transcriptional similarity between cortical regions. Spatially resolved microarray gene expression data were obtained from the Allen Human Brain Atlas^88^, preprocessed and mapped to the Schaefer 100 × 7 atlas using the abagen toolbox^125^. In brief, the preprocessing procedure includes intensity-based filtering, representative probe selection, tissue sample matching, normalization and aggregation^126^. The final region-by-region correlated gene expression matrix was estimated by calculating the Pearson’s correlation coefficient using normalized gene expression profiles across regions.

The laminar similarity network measures the similarity of cellular profiles across the cortical layers between pairs of regions. Histology-based cell-staining intensity values were derived from a postmortem brain, quantifying cell density and soma size^90,127,128^. Depth-resolved intensity values were sampled from 50 equivolumetric surfaces from white to pial surface. The intensity profiles were acquired on fsaverage surface using the BigBrainWarp toolbox^89,129^, and subsequently parcellated to the Schaefer 100 × 7 atlas. The region-by-region laminar similarity network was calculated using partial correlation, correcting for mean intensity across cortical regions.

Metabolic connectivity represents the cofluctuation of glucose metabolism between cortical regions. Volumetric FDG-PET images were recorded over time for 26 healthy participants^91,130^. PET images were reconstructed and preprocessed using a previously reported pipeline, resulting in 225 16-s fPET volumes for each recording^131^. They were subsequently motion corrected, underwent a spatial temporal gradient filter and were registered to the MNI152 template. Finally, they were parcellated to the Schaefer 100 × 7 atlas, and the metabolic connectivity matrix was calculated as Pearson’s correlation coefficient for each participant. The group-averaged matrix was used in this project.

The receptor similarity network measures the similarity of receptor density profiles between regions. PET tracer data for 18 neurotransmitter receptors and transporters were taken from ref. ^49^ and neuromaps (v0.0.1, https://github.com/netneurolab/neuromaps (ref. ^132^)). The neurotransmitter systems include dopamine (D_1_^133^, D_2_^134–137^ and DAT, dopamine transporter^138^), norepinephrine (NET, norepinephrine transporter^139–142^), serotonin (5-HT_1A_^143^, 5-HT_1B_^143–150^, 5-HT_2_^151^, 5-HT_4_^151^, 5-HT_6_^152,153^ and 5-HTT^151^), acetylcholine (α_4_β_2_^150,154^, M_1_^155^ and VAChT, vesicular acetylcholine transporter^156,157^), glutamate (mGluR_5_^158,159^), GABA (GABA_A_^160^), histamine (H_3_^161^), cannabinoid (CB_1_^162–165^) and opioid (MOR, mu opioid receptor^166^). Each PET image was parcellated to the Schaefer 100 × 7 atlas. The final receptor similarity matrix was calculated as Pearson’s correlation coefficient between the receptor profiles for pairs of regions.

### Fingerprinting

Fingerprinting of individual differences was calculated using the identifiability metric proposed in refs. ^51,54^.

$$\,\text{Identifiability}\,=\frac{| {\mu }_{{\rm{intra}}}-{\mu }_{{\rm{inter}}}| }{s}.$$

For each pairwise statistic, four matrices from BOLD runs per participant were used to calculate the mean values of within-participant correlations *μ*_intra_ and between-participant correlations *μ*_inter_. The pooled standard deviation *s* is also estimated. The resulting measure of identifiability is analogous to an effect size statistic^54^.

### Behavior prediction

We used a robust set of ICA-derived cognitive-behavioral phenotypes derived by^55^. Briefly, HCP behavioral dataset were filtered for measures related to alertness, cognition, emotion, sensory-motor function, personality, psychiatric symptoms, substance use, and life function. A total of 109 measures were selected and subjected to an ICA procedure. Before the ICA procedure, normalization (87 out of 109) and confound regression (age and sex) were carried out to clean the raw behavioral data. The consistency and reliability of the ICA procedure was validated with bootstrapping and agglomerative clustering, followed by a sampling and matching process. A five-component model emerged as the most robust and concise representation of the original data structure: cognitive performance, illicit substance use, tobacco use, personality and emotion traits, and mental health. Details of the can be found in ref. ^55^. The intersecting *N* = 310 participants were used for this study.

For the pairwise interaction measures, we used the vectorized upper triangular values of the SPI matrices for each participant, averaged across the four BOLD runs. To make the prediction more robust, we filtered the data using quartile coefficient of dispersion (QCoD) to provide a conservative representation of the predictor vector. We first calculated QCoD across participants for each SPI and excluded those with minimal variance for all region pairs (absolute maximum QCoD <0.01; pli_multitaper_max_fs-1_fmin-0_fmax-0-25, pli_multitaper_max_fs-1_fmin-0-25_fmax-0-5, wpli_multitaper_max_fs-1_fmin-0_fmax-0-25). For each prediction, we further calculated the 10th and 90th QCoD percentile and included only the region pairs within this range to avoid spurious values with very large or little variance that may affect the prediction.

Following previous best practices^52,53,56,167^, we used kernel ridge regression with linear kernel for behavior prediction. We set up the prediction pipeline with nested *k*-fold cross-validation. The inner tenfold cross-validation loop was used to select the optimal regularization parameter *α*, and the final performance was evaluated in the independent test split in the outer tenfold cross-validation loop. Both training and testing data were standardized using statistics estimated only from the training data to avoid leakage. We calculated Pearson’s correlation between empirical and predicted values for the final evaluation. The same process was also repeated with kernel ridge regression with cosine kernel, linear ridge regression and LASSO regression. A comparison of average performance and variability across the 49 pairwise measures is shown in Supplementary Fig. 8 and Supplementary Fig. 9, respectively. The performance details for each of the 239 individual statistics are shown in Supplementary Fig. 10.

### Integrated information decomposition (ΦID)

We used integrated information decomposition (ΦID^59–61^), a temporally extended framework of partial information decomposition (PID^58,168,169^) to estimate the information flow patterns (‘information flow atoms’).

The original PID framework aims to study the multivariate information by jointly considering multiple source variables with an additional target variable. As shown in Fig. 5a, in a two-variable scenario, *I*(*R*_1_; *X*), *I*(*R*_2_; *X*) and *I*(*R*_1_, *R*_2_; *X*) represent their specific information, quantifying the information provided by the source variables when provided the information about the target variable *X*. PID decomposes the information contents into their unique information components (Unq(*R*_1_; *X*) and Unq(*R*_2_; *X*)), a redundant information component (RED(*R*_1_, *R*_2_; *X*)) and a synergistic information component (SYN(*R*_1_, *R*_2_; *X*)). Here, the term ‘redundant’ suggests information identically provided by each of the two variables individually, and the term ‘synergistic’ suggests new information that emerges when the two variables are considered together (see refs. ^58,60,61,168^ for formal definitions).

ΦID extends this framework by introducing a temporal dimension. Taking a pair of time series as inputs, ΦID defines a past and a future state, and derives 16 information flow atoms, denoted as pairwise transitions between the initial four information atoms. The 16 types of information flow can be mechanistically categorized into several types: storage (information that remains carried in the same way over time; Red → Red, Un^1^ → Un^1^, Un^2^ → Un^2^ and Syn → Syn), duplication (information that becomes redundantly available from both variables, and was not before; Un^1^ → Red and Un^2^ → Red), migration (information that moves between variables, such that it was uniquely present in a single variable, and subsequently it is uniquely present in the other; Un^1^ → Un^2^ and Un^2^ → Un^1^), deduplication (information that is pruned from duplication, such that it is no longer redundant; Red → Un^1^ and Red → Un^2^), decryption (collective/distributed information that becomes individual information in the future, also known as downward causation; Syn → Un^1^, Syn → Un^2^ and Syn → Red), and encryption (individual information that becomes collective/distributed information in the future, also known as upward causation; Un^1^ → Syn, Un^2^ → Syn and Red → Syn) (see refs. ^59–61^ for more rigorous definitions).

Technically, ΦID requires a choice of how redundancy is defined, just like PID. Here, we chose the minimum mutual information definition of redundancy, following previous work^59,61,170,171^. Overlapping segments of the functional time series with one time step delay were used to define the past and future states. We calculated ΦID for every pair of the original functional time series using time-delayed mutual information (mutual information between the past and future states) under the Gaussian assumption for continuous variables. This process generated 16 information flow matrices (Fig. 5b). Note that there are many potential implementations of redundancy and temporal states; here we adopt a straightforward definition as previously validated in refs. ^59–61^. An open-source implementation can be found at https://github.com/Imperial-MIND-lab/integrated-info-decomp.

To establish the relationship between information flow atoms and pairwise interaction statistics (Fig. 5c), we constructed linear models utilizing the former as predictors and the latter as the outcome. We used dominance analysis^172,173^ to quantify the contribution of individual predictors in the presence of potential multicollinearity^174^. The ‘total dominance’ statistic is used to calculate the relative contribution of each predictor compared with the goodness of fit (*R*^2^) of the full linear model. The function is implemented in netneurotools (https://github.com/netneurolab/netneurotools), which is adapted from the Dominance-Analysis package (https://github.com/dominance-analysis/dominance-analysis).

### Sensitivity analyses

For sensitivity analyses, the time series were additionally parcellated into the Desikan–Killiany atlas^175^ and Schaefer 200-node 7-network atlas^96^. They also underwent global signal removal. To effectively calculate the sensitivity analysis using a higher atlas resolution with 200 regions, we generated a minimized list of SPIs by removing those taking more than 30 min to calculate for a single participant, resulting in 197 SPIs calculated. After taking the intersection with the list of SPIs above, 179 SPIs were used for the sensitivity analysis (see Supplementary Table 2 for the full list of SPIs used). The group-average measure similarity matrix shown in Fig. 1 was calculated for each scenario. The similarity matrices were 239 by 239 in dimension, except when the reduced set of measures was used, which gives matrices of 179 by 179 in dimension, and matched elements from the 239-by-239 matrix were extracted for comparison. Spearman’s rank correlation coefficient was calculated between the upper triangular elements to quantify the correlation.

### Reporting summary

Further information on research design is available in the Nature Portfolio Reporting Summary linked to this article.

## Online content

Any methods, additional references, Nature Portfolio reporting summaries, source data, extended data, supplementary information, acknowledgements, peer review information; details of author contributions and competing interests; and statements of data and code availability are available at 10.1038/s41592-025-02704-4.

## Supplementary information

Supplementary Information Supplementary Note 1, Figs. 1–20 and Tables 1–5.

Reporting Summary

## Data Availability

The HCP data are available at https://db.humanconnectome.org/data/projects/HCP_1200 (ref. ^41^). Multimodal neurophysiological networks (including the Neurosynth-derived cognitive similarity network) are available via GitHub at https://github.com/netneurolab/hansen_many_networks (ref. ^84^). Behavioral phenotypes are available via GitHub at https://github.com/yetianmed/subcortex (ref. ^55^). The raw pyspi outputs and the singularity container used for calculation are available at https://osf.io/75je2/.

## Code availability

The code and scripts used for analysis are available via GitHub at https://github.com/netneurolab/liu_fc-pyspi, distributed under the BSD 3-Clause license. The calculation was performed using a singularity container available (https://osf.io/75je2/) for reproducibility. The pyspi package is available via GitHub at https://github.com/DynamicsAndNeuralSystems/pyspi (ref. ^26^) under GNU General Public License v3.0.
