# Supplementary material for: Benchmarking methods for mapping functional connectivity in the brain
Source: Nat Methods. 2025 Jun 6;22(7):1593–602. doi: 10.1038/s41592-025-02704-4 (PMC12240839; doi:10.1038/s41592-025-02704-4)
Supplement: Supplementary file 1 — Supplementary Note 1, Figs. 1–20 and Tables 1–5. [file 41592_2025_2704_MOESM1_ESM.pdf]

nature methods

Supplementary information https://doi.org/10.1038/s41592-025-02704-4

Benchmarking methods for mapping functional connectivity in the brain

In the format provided by the authors and unedited

Supplementary Note 1. Validation across datasets

We included a set of validation datasets: HCP–Retest (N=20), AOMIC–PIOP1 (N=216), AOMIC–PIOP2 (N=226), MSC (N=10), MPI–MBB (N=126), and RBC–NKI (N=592). For all the datasets, we calculated a reduced set of pairwise interaction statistics (also used for validation of higher resolution parcellation in Fig. 6c; see Supplementary Table 2 for a full list) from the *pyspi* toolbox using the same procedure as in the main text. A detailed list of datasets with their acquisition and preprocessing characteristics follows.

A. HCP–Retest

Human Connectome Project Healthy Young Adult (HCP–YA) Retest dataset consists of 46 subjects that underwent the full 3T HCP imaging and behavioral acquisitions for a second time [1–3]. We selected 20 subjects intersecting with the 326 unrelated subjects from our main analyses. We used officially preprocessed data from HCP S1200 release. The original preprocessing includes MSMAll registration and ICA–FIX denoising (see [4] for full preprocessing details). Preprocessed data in fsLR 32k space were downloaded from HCP S3 repository and parcellated to Schaefer 100-node 7-network atlas using Connectome Workbench. No additional preprocessing steps were applied.

B. AOMIC–PIOP1 and AOMIC–PIOP2

The Amsterdam Open MRI Collection (AOMIC) dataset is a large collection of neuroimaging and psychometric data from healthy participants [5]. We used two datasets from this collection: PIOP1 (N=216) and PIOP2 (N=226). The resting-state fMRI acquisitions used a gradient-echo EPI sequence. For PIOP1, multiband scans were acquired with 3mm isotropic voxels, TR=750mm, and 6 minutes total acquisition time. For PIOP2, single-band scans were acquired with 3mm isotropic voxels, TR=2000mm, and 8 minutes acquisition time. We used officially preprocessed data release from the AOMIC project. The original preprocessing steps used fMRIPrep and details can be found in [5, 6]. No further denoising steps were applied. Preprocessed data in fsaverage5 space were downloaded from openneuro.org S3 repository and parcellated to Schaefer 100-node 7-network atlas. Parcellated time series were demeaned and z-scored before pyspi calculation.

C. MSC

Midnight Scanning Club (MSC) is an intensively sampled dataset on 10 individuals [7]. Each subject has 10 scan sessions, with around 10 hours of resting-state and task scans in total. The resting-state fMRI acquisitions used a gradient-echo EPI sequence with 4mm isotropic voxels, TR=2200ms, and 300 minutes total acquisition time. We used officially preprocessed data release from the MSC project. The original preprocessing steps used a custom pipeline, and details can be found in [7]. Denoising steps include motion censoring and confound regression. Preprocessed data in fsLR 32k space were downloaded from openneuro.org S3 repository and parcellated to Schaefer 100-node 7-network atlas using Connectome Workbench.

D. MPI–MBB

Max Planck Institut Leipzig Mind-Brain-Body Dataset – LEMON dataset is a cross–sectional mind-body-emotion interaction study [8, 9]. The resting-state fMRI acquisitions used a multiband gradient-echo EPI scan sequence with 2.3 mm isotropic voxels, TR=1400ms, and 15.5 minutes total acquisition time. We used officially preprocessed data release from the MPI–MBB project. The original preprocessing steps used a custom Nipype–based pipeline, and details can be found in [8, 9]. Denoising steps include Nipype rapidart and aCompCor. Preprocessed data from 126 subjects with resting-state fMRI in MNI152 2mm space were downloaded from openneuro.org S3 repository and parcellated to Schaefer 100-node 7-network atlas using nilearn.

E. RBC–NKI

Enhanced Nathan Kline Institute – Rockland Sample (NKI–RS) dataset is a large-scale advanced neuroimaging study across the lifespan [10, 11]. For resting-state fMRI acquisitions, three different gradient echo EPI scan sequences were acquired, including two multiband sequences at TR=645ms (3mm isotropic voxels, 10 minutes, optimal temporal resolution) and TR=1400ms (2mm isotropic voxels, 10 minutes, optimal spatial resolution), and one single-band sequence at TR=2500ms (3mm isotropic voxels, 5 minutes). We used preprocessed data release from the Reproducible Brain Charts (RBC) project [12]. The original preprocessing steps used C-PAC and details can be found at `reprobrainchart.github.io`. Two types of denoising methods were available: explicit motion regression (36Parameter) and decomposition-based approach (aCompCor). We selected 592 subjects with all three resting-state scans

(645, 1400, and CAP) for the baseline visit (BAS1). Preprocessed time series in Schaefer 200-node 17-network atlas were downloaded using Datalad.

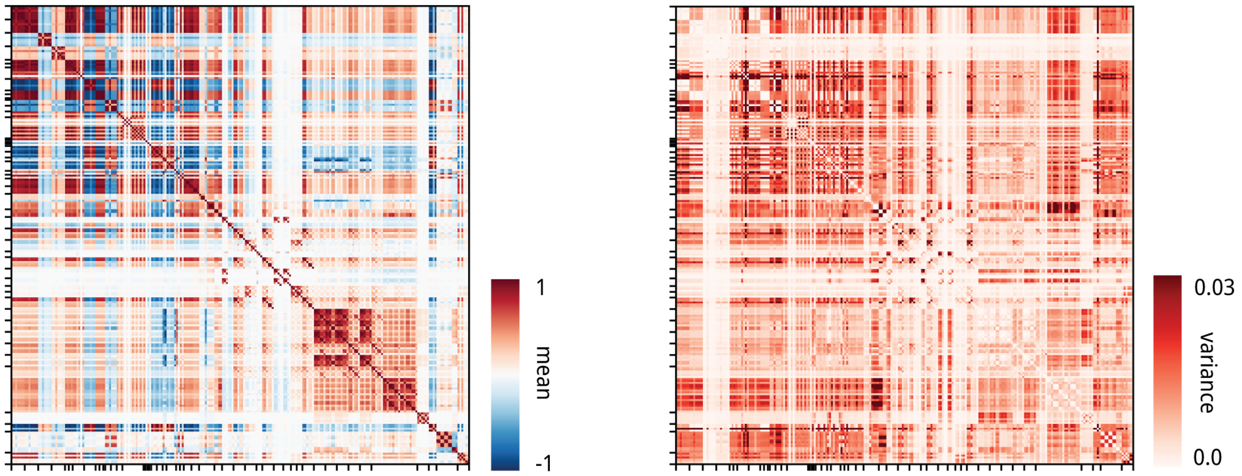

Supplementary Figure 1 | **Similarity profile (Left)** Group mean (same as in Fig. 1), and **(Right)** group variance matrices calculated from the individual similarity profiles across subjects and runs.

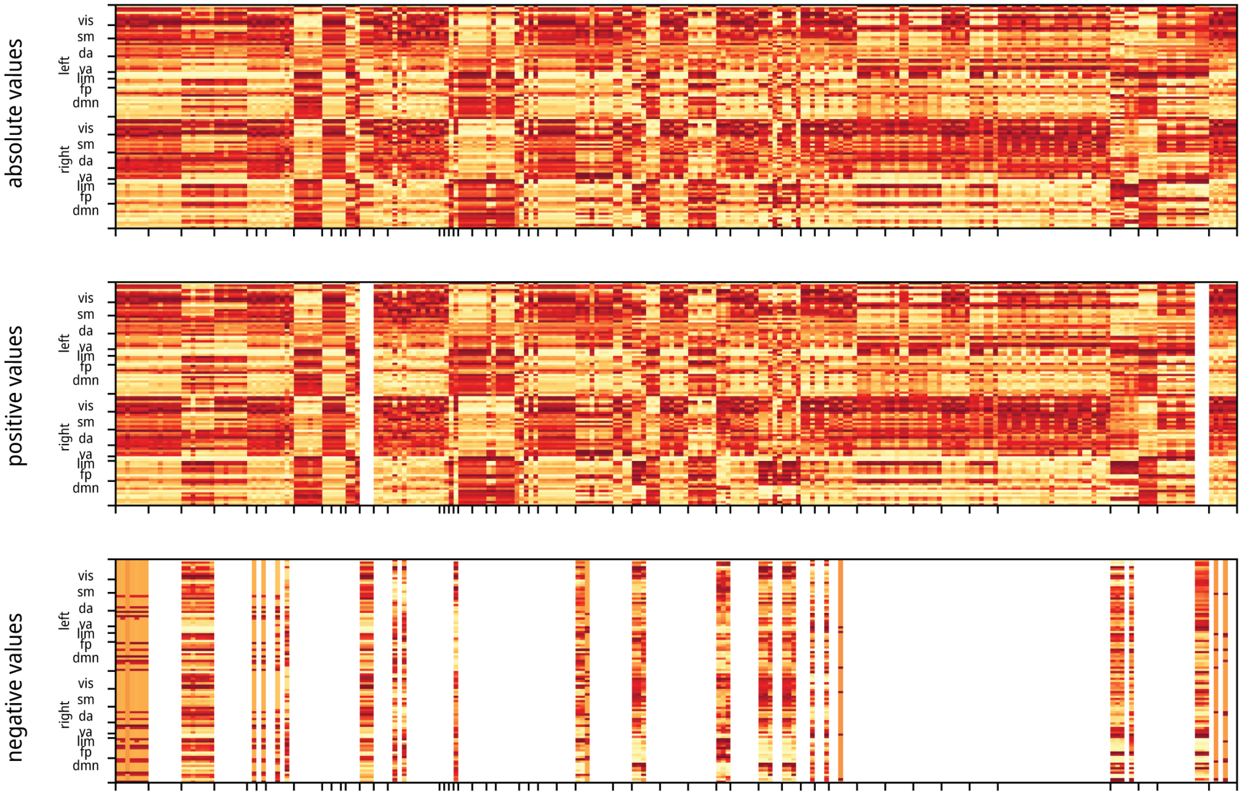

Supplementary Figure 2 | **Hubness** Ranking of hubs quantified by weighted degree of the pairwise statistic matrices, shown for (from top to bottom) absolute values, positive values only, and negative values only. Rankings have not been flipped like in Fig. 2b. Regions are ordered by intrinsic functional networks from [13] for left and right hemispheres. Darker red means more hubness. VIS: visual, SM: somatomotor, DA: dorsal attention, VA: ventral attention, LIM: limbic, FP: fronto-parietal, DMN: default mode network.

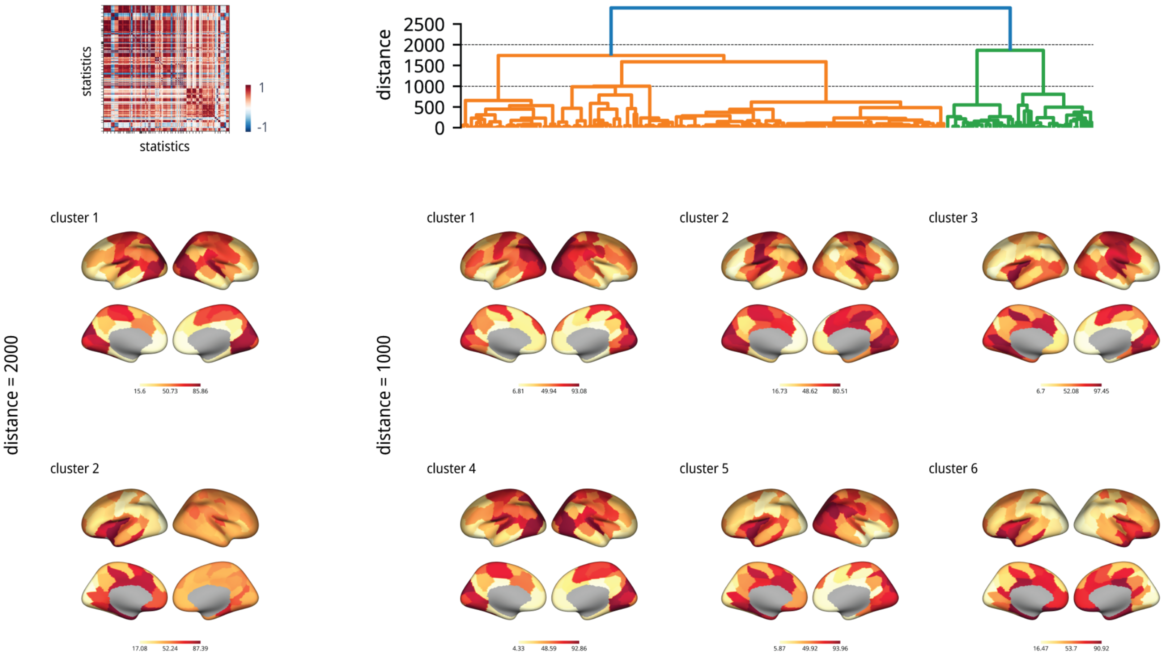

Supplementary Figure 3 | **Hubness clusters** Hierarchical clustering of the hubness results in Fig. 2b. **(Upper left)** Similarity between the hubness results between the statistics in Fig. 2b. **(Upper right)** Dendrogram showing hierarchical clustering of the hubness results. **(Lower left)** The two clusters at $d = 2000$ averaged across statistics and shown on the cortical surface. **(Lower right)** The six clusters at $d = 1000$ averaged across statistics and shown on the cortical surface. Hierarchical clustering is implemented using *scipy* with *ward* linkage method.

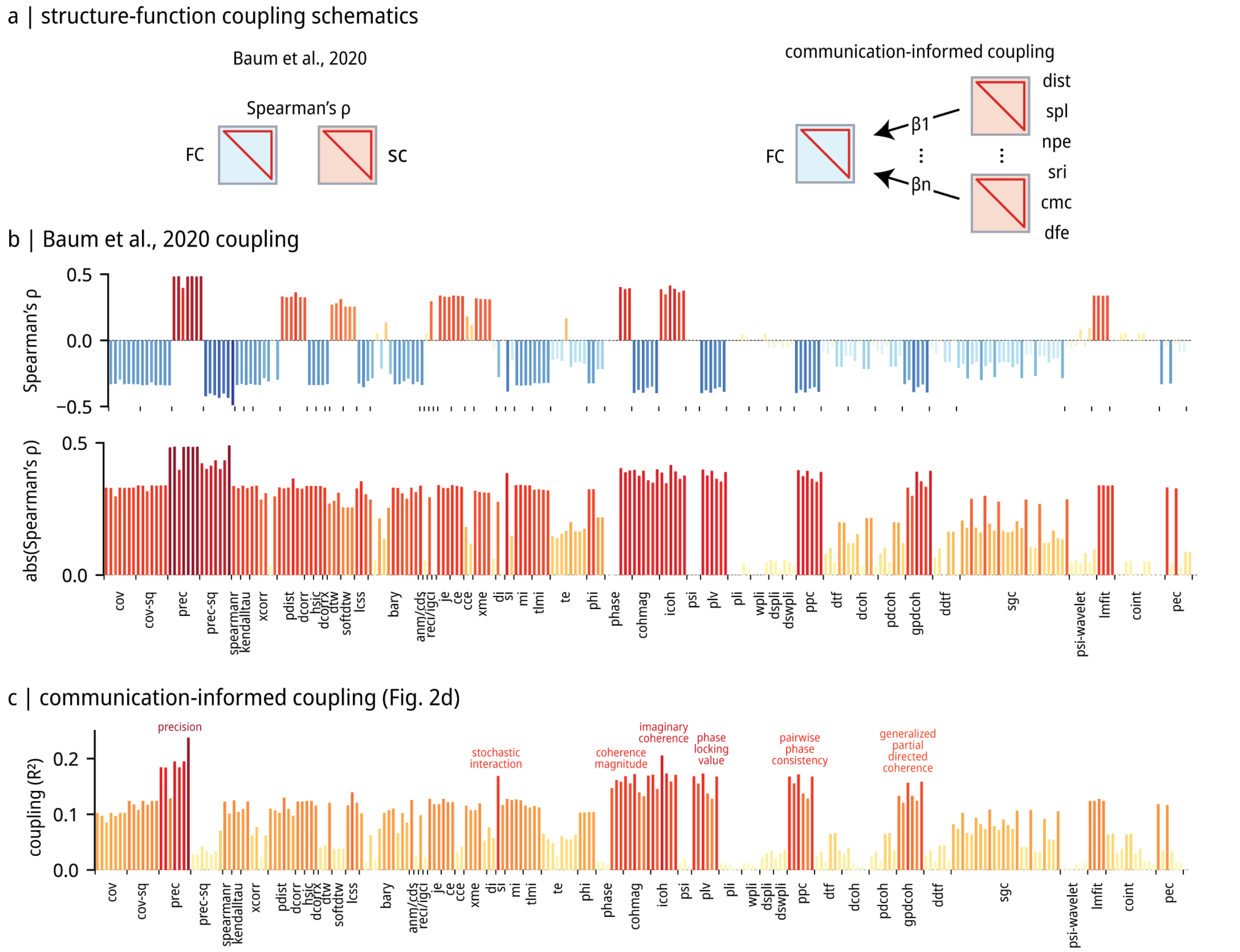

Supplementary Figure 4 | **Structure–function coupling between structural connectivity and matrices of interaction statistics** (a) Schematics showing two structure–function coupling estimation approaches: simple Spearman correlation in panel (b) and multilinear regression model in panel (c). (b) Structure–function coupling calculated as the Spearman's rank correlation coefficient following [14], using the 2272 non-zero elements of the structural connectivity and corresponding values in the FC matrices. (c) Structure–function coupling calculated as the coefficient of determination (adjusted R^2) of a multilinear regression model [15, 16] in Fig. 2d. Communication models matrices derived from structural connectivity are used as the structural predictors.

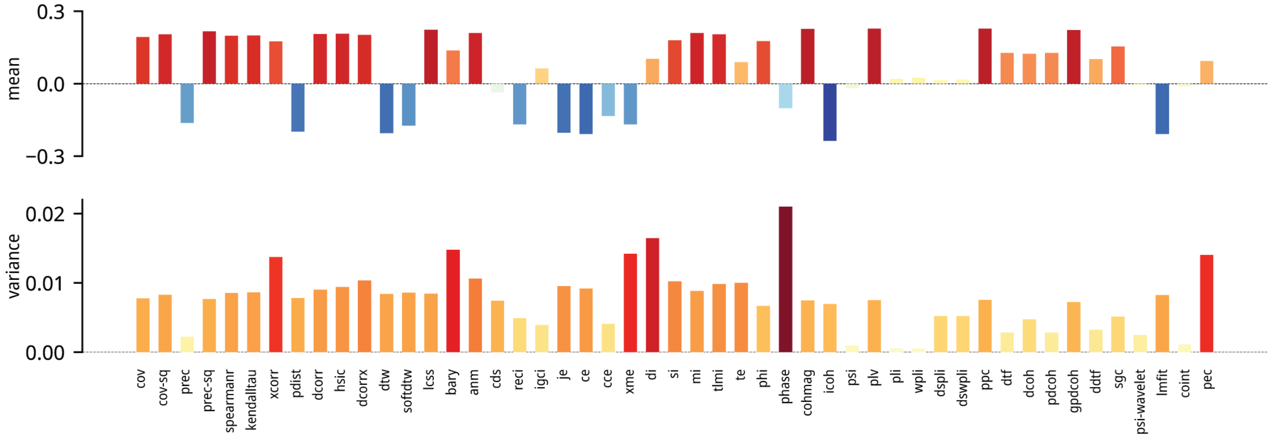

Supplementary Figure 5 | **Multimodal neurophysiological networks** Correlations between statistics and the neurophysiological networks summarized across the five networks (correlated gene expression, laminar similarity, neurotransmitter receptor similarity, electrophysiological connectivity, and metabolic connectivity), showing **(Upper)** mean and **(Lower)** variance across the 49 measures.

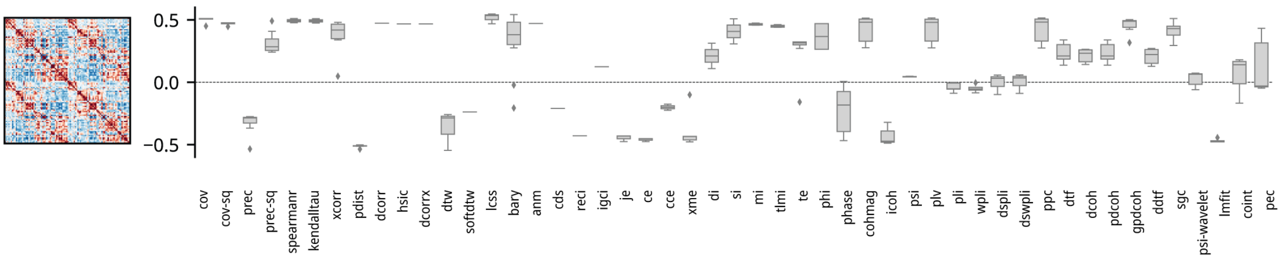

Supplementary Figure 6 | **Correlation with cognitive similarity network** Similar to Fig. 3, pairwise interaction statistics matrices were correlated with inter-regional cognitive similarity network. The box plot shows the median as the center line, upper and lower quantiles as box limits, 1.5x interquartile range as whiskers, and outliers as points. Cognitive similarity network was estimated from regional functional associations of Neurosynth terms. Neurosynth is a meta-analytical database containing the voxel coordinates and related high-frequency keywords for >15,000 fMRI studies [17]. Briefly, we selected 123 neurocognitive terms from the Cognitive Atlas, a public ontology for cognitive science [18], and estimated a probabilistic measure of association for each term. The probabilistic measure can be interpreted as a quantitative account of regional activation relating to the neuropsychological process. A detailed methodological description can be found in **(author?)** [19].

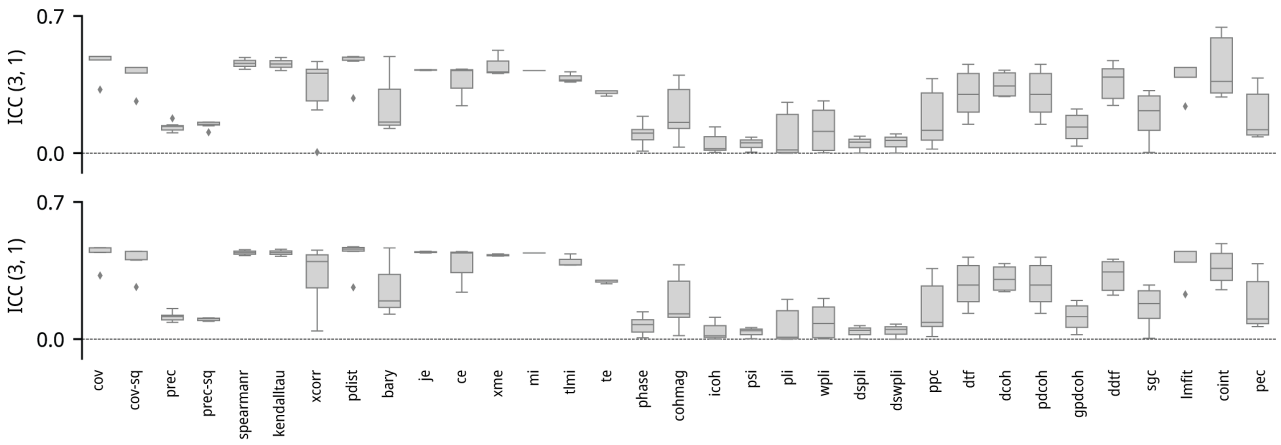

Supplementary Figure 7 | **Test–retest analysis on the HCP and HCP Retest dataset** (Upper) Using 326 subjects, each with 4 resting-state runs from HCP–YA dataset. (Lower) Using 20 subjects, each with 4 resting-state runs from HCP dataset and 4 additional runs from HCP Retest dataset. Intraclass correlation (ICC) was calculated for each pairwise statistic. We used ICC$(3, 1)$ implemented following *PyReliMRI* [20] and was defined in detail by **(author?)** [21]. Note that a reduced list of 179 pairwise statistics (Supplementary Table 2) were used for the HCP dataset in order to make the results compatible with the validation dataset.

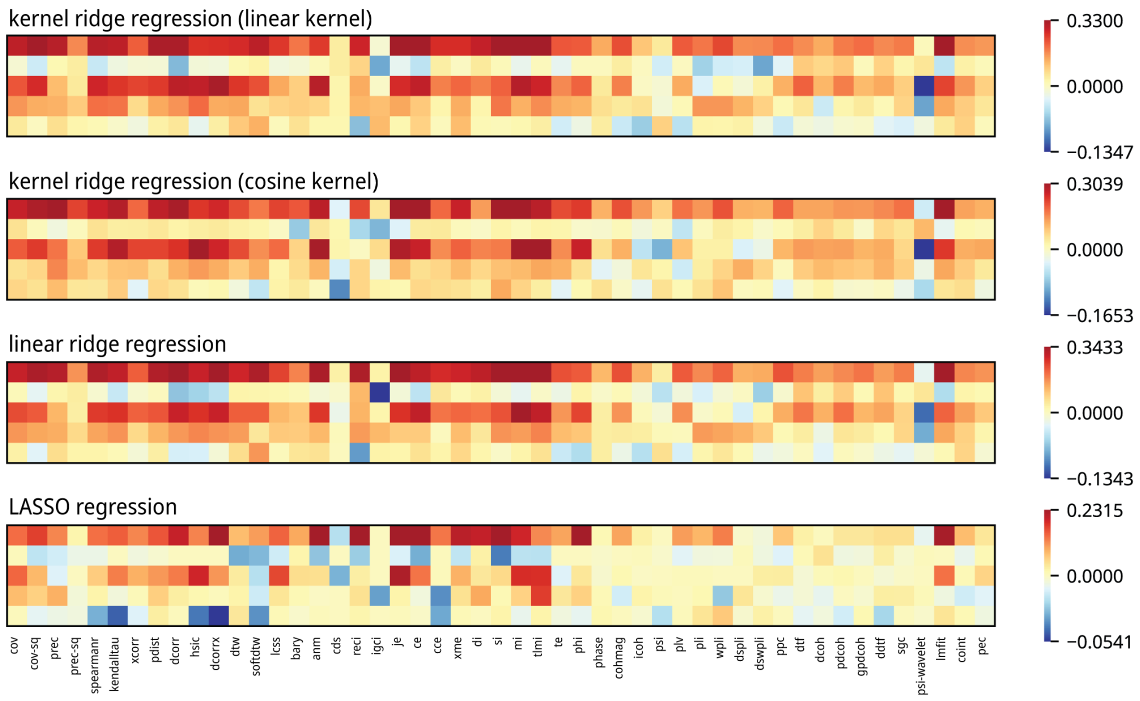

Supplementary Figure 8 | **Behavior prediction** Mean prediction results from Fig. 4b (kernel ridge regression using linear kernel) are shown with alternative statistical learning algorithms, including kernel ridge regression (cosine kernel), linear ridge regression, and LASSO regression. The colorbars cover both negative values (0th percentile to 0; in blue) and positive values (0 to 97.5th percentile; in red).

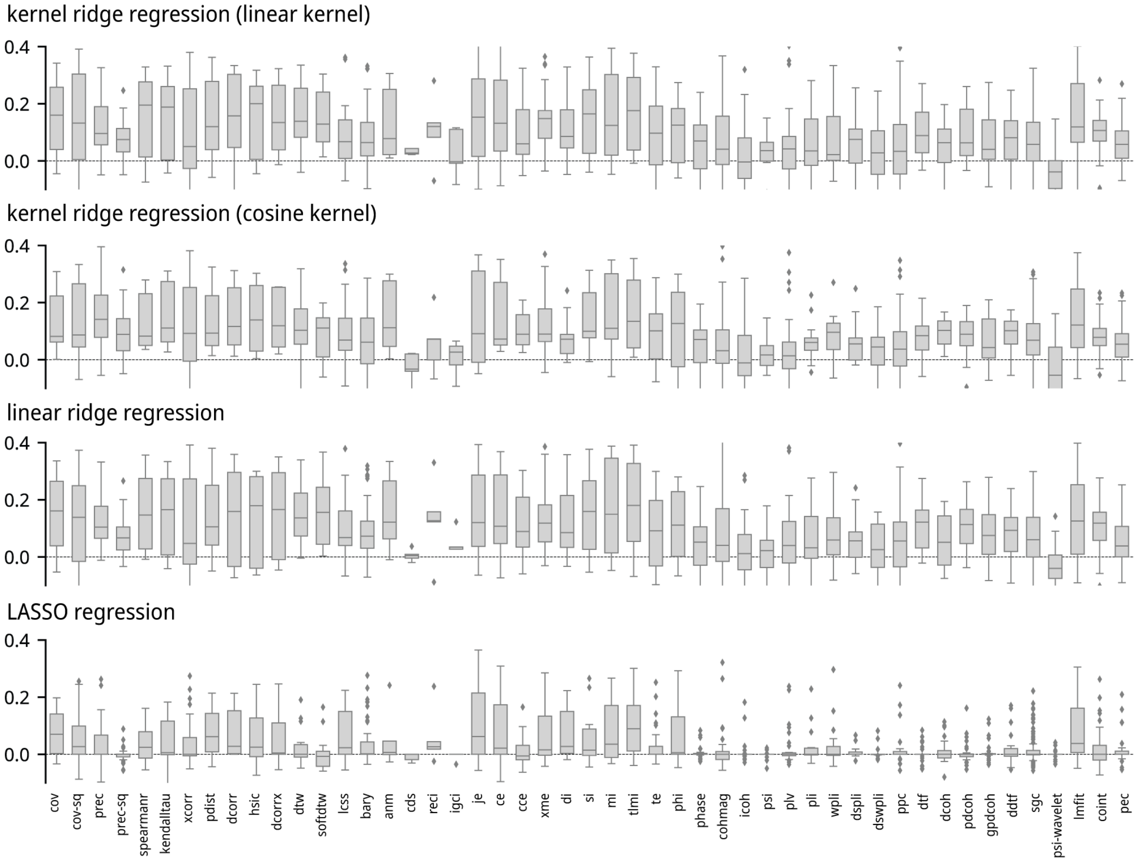

Supplementary Figure 9 | **Behavior prediction** Prediction results from Supplementary Fig. 8 are displayed in box plots, showing individual statistic variations within each of the 49 measures. Box plots show the median as the center line, upper and lower quantiles as box limits, 1.5x interquartile range as whiskers, and outliers as points.

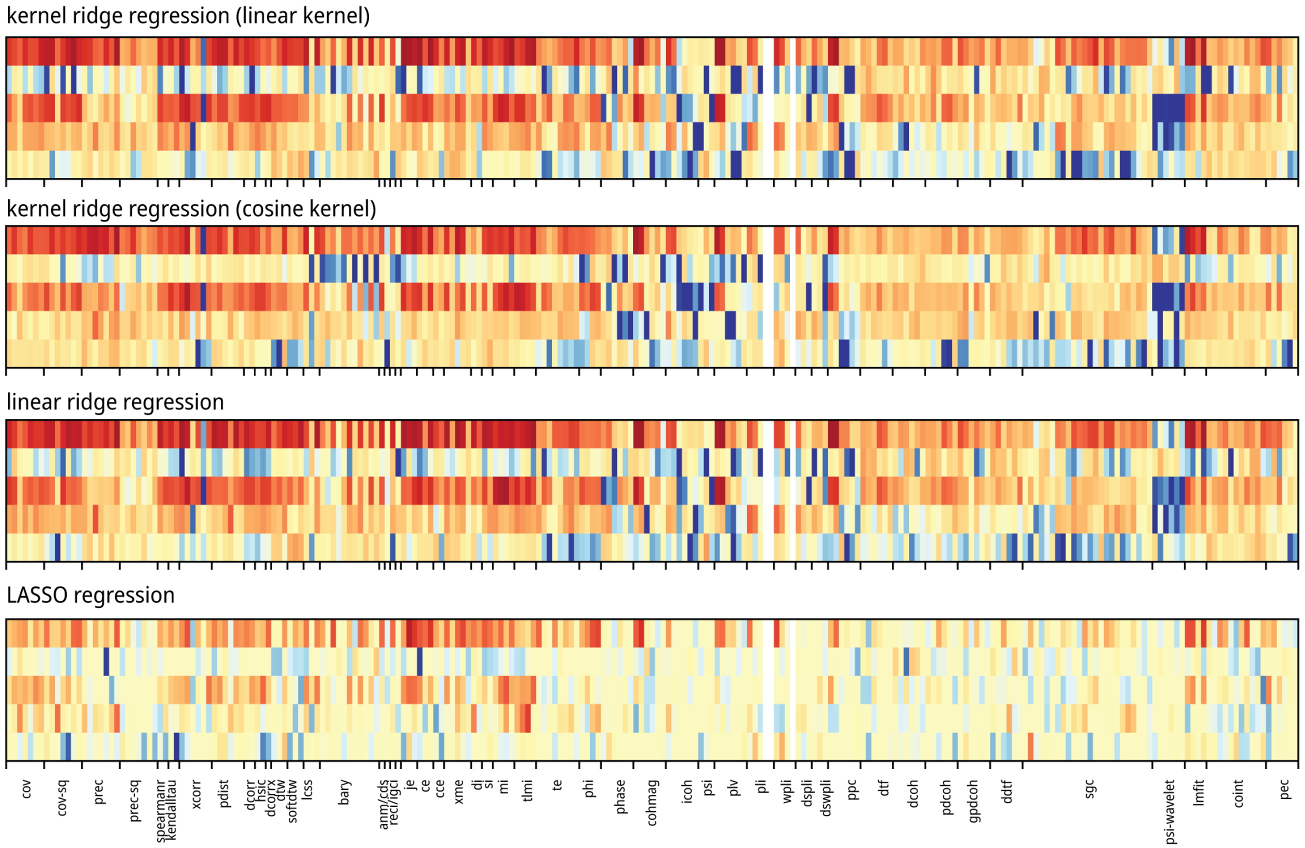

Supplementary Figure 10 | **Behavior prediction** Mean prediction results from Supplementary Fig. 8 are displayed for each of the 239 statistics.

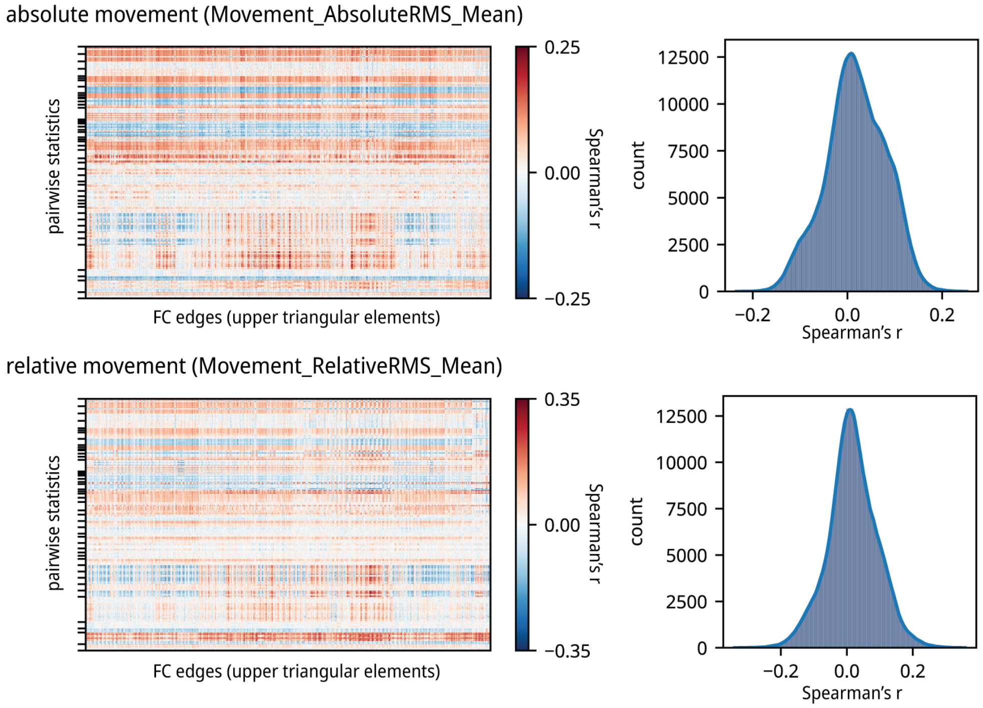

Supplementary Figure 11 | **Sensitivity of pairwise statistics to participant movement** Root mean square of mean absolute (Upper) and relative (Lower) movement data during resting-state fMRI acquisition were correlated with each pairwise interaction statistic.

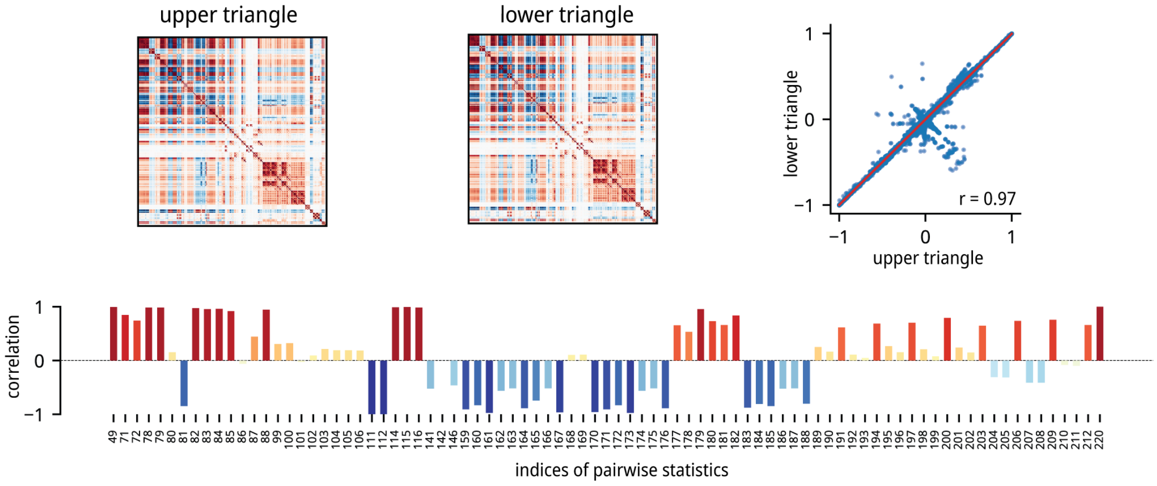

Supplementary Figure 12 | **Directed measures in the pairwise statistics** We extracted the statistics with different upper and lower triangular values (excluding those which only differ by a sign) and study the implication of their directness. On the upper row, the group mean similarity profile (as in Fig. 1) was calculated using upper triangular values (used in the main results) and lower triangular values. Their correlation was calculated similar to Fig. 6. On the bottom row, Spearman's rank correlations between the upper and lower triangular values were calculated for each directed pairwise statistics matrix. The indices on the x-axis correspond to the indices in Supplementary Table 1. A full list of directed pairwise statistics is shown in Supplementary Table 3. The list of pairwise statistics with only sign changes is shown in Supplementary Table 4

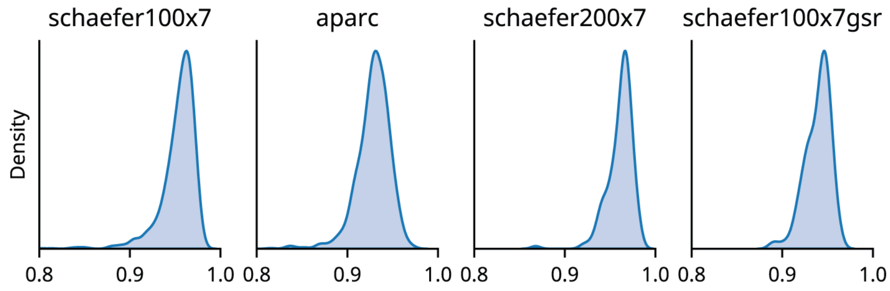

Supplementary Figure 13 | **Correlation distribution between each subject's and the group average term similarity profile** for (from left to right) schaefer100×7 parcellation (used in the main results), Desikan–Killiany parcellation, schaefer200×7 parcellation, and schaefer100×7 parcellation under global signal regression.

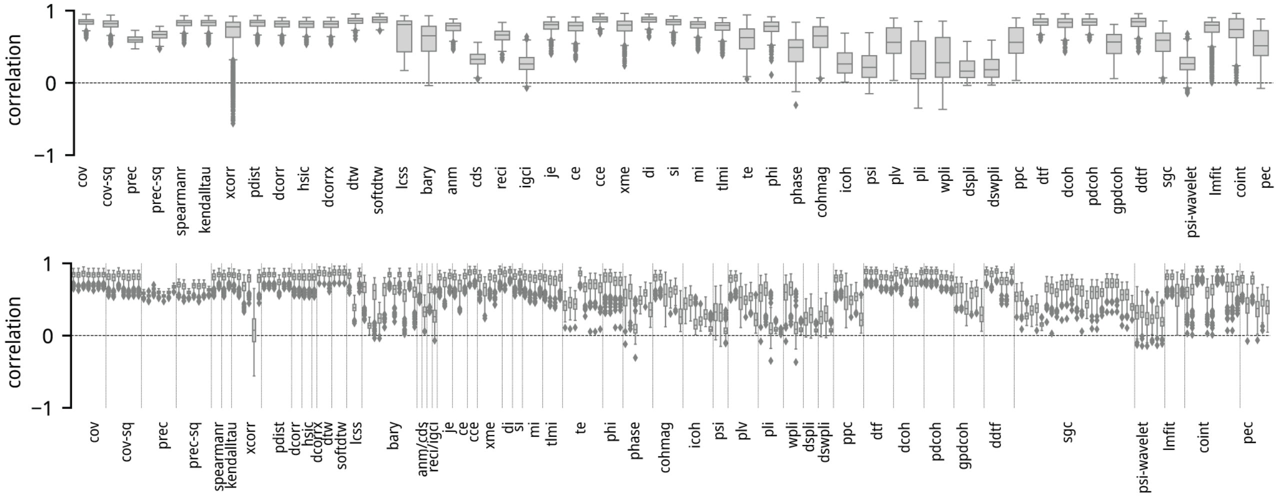

Supplementary Figure 14 | **Correlation distribution between each subject's and group average FC for 239 pairwise statistics** Spearman's rank correlation was taken using the upper triangular elements of the schaefer100×7 parcellation matrices. Shown by 47 categories of measures (Upper) and by 239 individual measures (Lower).

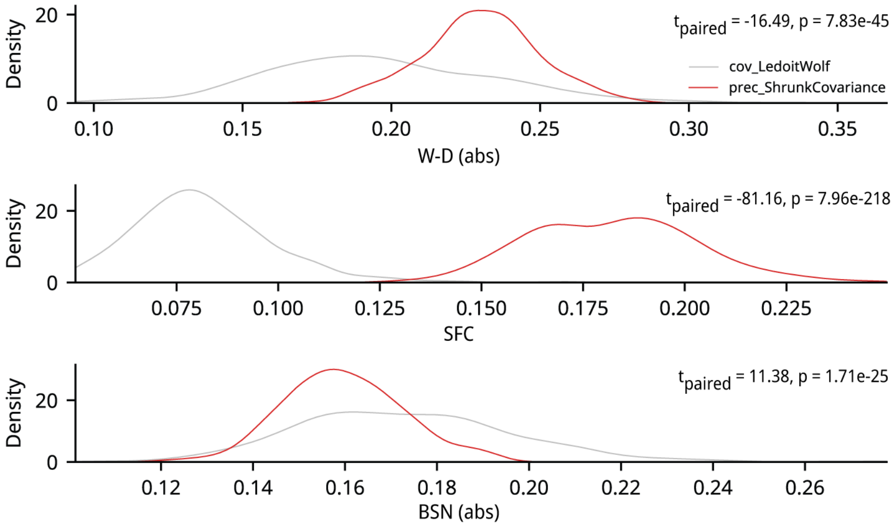

Supplementary Figure 15 | **Comparison of** prec_ShrunkCovariance **and** cov_LedoitWolf **across individuals** Three analyses from our study suitable for individual–level replication are carried out (from top to bottom): weight–distance relationship (W-D), structure–function coupling (SFC), and correspondence with biological similarity networks (BSN). For W-D and BSN, absolute values were taken to make sure covariance and precision estimators are comparable. Paired t tests were carried out between *cov_LedoitWolf* and *prec_ShrunkCovariance* for weight–distance relationship ($t = -16.49$, $p = 7.83 \times 10^{-45}$, Cohen's $d = 1.09$), structure–function coupling ($t = -81.16$, $p = 7.96 \times 10^{-218}$, Cohen's $d = 5.36$), and biological similarity networks ($t = 11.38$, $p = 1.71 \times 10^{-25}$, Cohen's $d = 0.72$). Negative t statistic indicates *cov_LedoitWolf* has smaller values than *prec_ShrunkCovariance*, and vice versa.

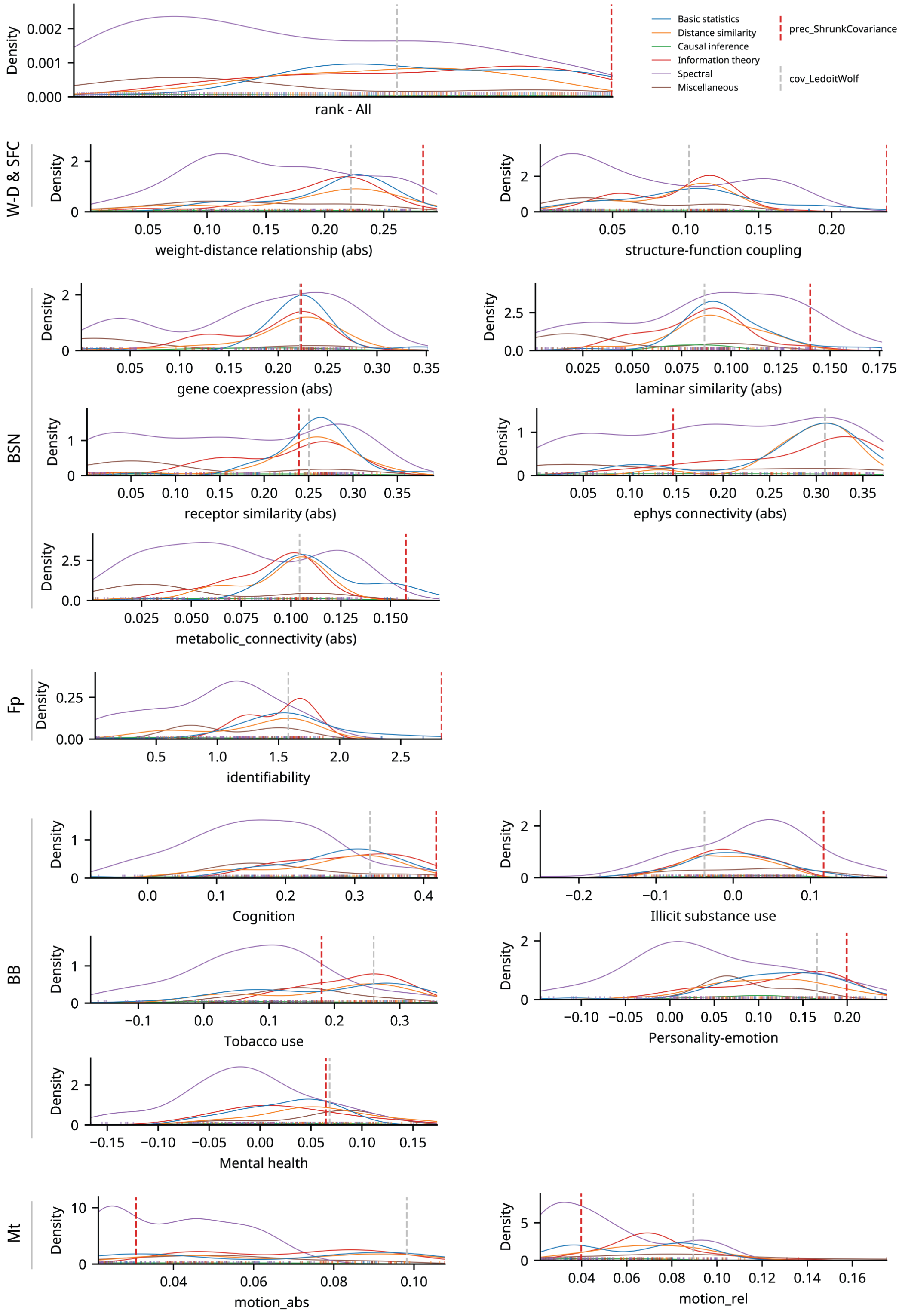

Supplementary Figure 16 | **Value distribution for ranking the pairwise interaction statistics** Raw values to produce the ranking in Supplementary Table 5 were shown as distributions grouped by measure categories. The red vertical line indicates the top-performing statistic (*prec_ShrunkCovariance*) and the gray vertical line shows a commonly used statistic (*cov_LedoitWolf*) similar to Pearson's correlation coefficient. The top panel shows the final ranking, followed by raw values from individual criteria. The criteria used are (from top to bottom) weight–distance relationship (W-D), structure–function coupling (SFC), correspondence with biological similarity networks (BSN), individual–participant identifiability (Fp), brain–behavior prediction (BB), and susceptibility to participant motion (Mt).

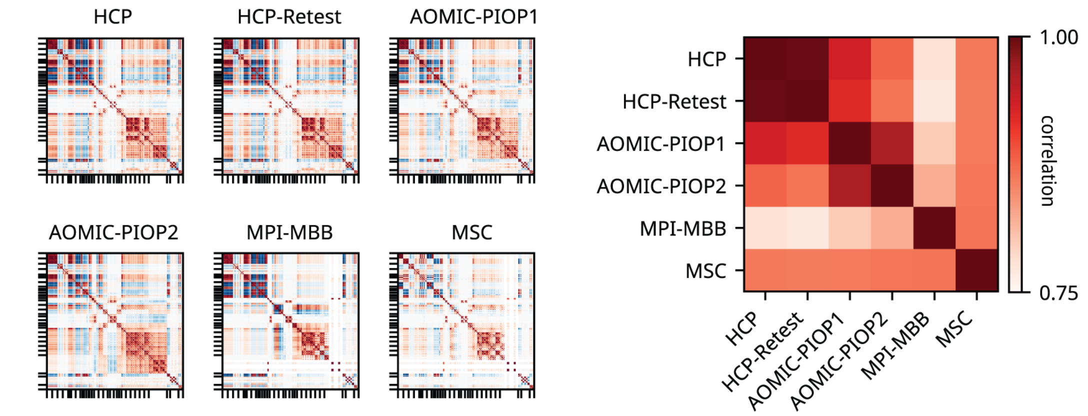

Supplementary Figure 17 | **Group-average term similarity profile across datasets** Similarity profile for HCP, HCP–Retest, AOMIC–PIOP1, AOMIC–PIOP2, MPI–MBB, and MSC. (Right) Comparison of group-average term similarity profile across datasets.

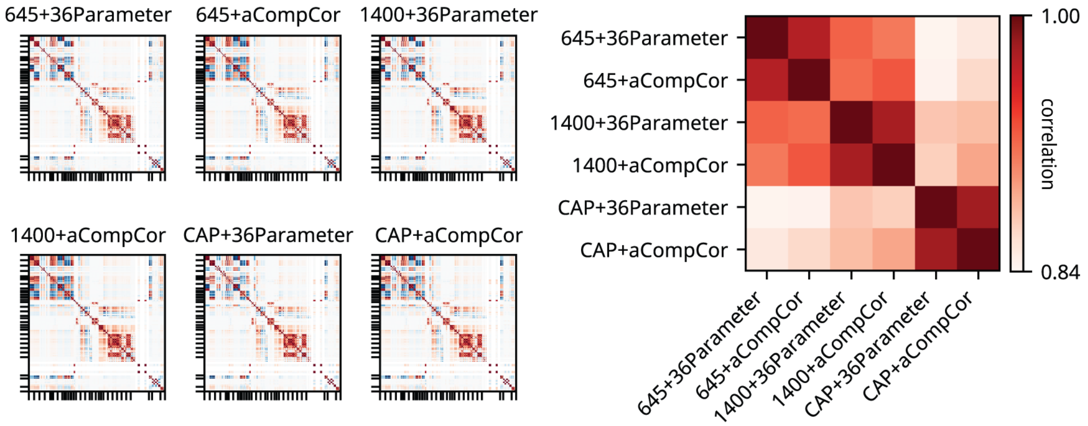

Supplementary Figure 18 | **Group-average term similarity profile across acquisition and motion correction conditions using the RBC–NKI dataset** (Left) Similarity profile for 6 conditions. (Right) Comparison of group-average term similarity profile across acquisition and motion correction conditions using the RBC–NKI dataset.

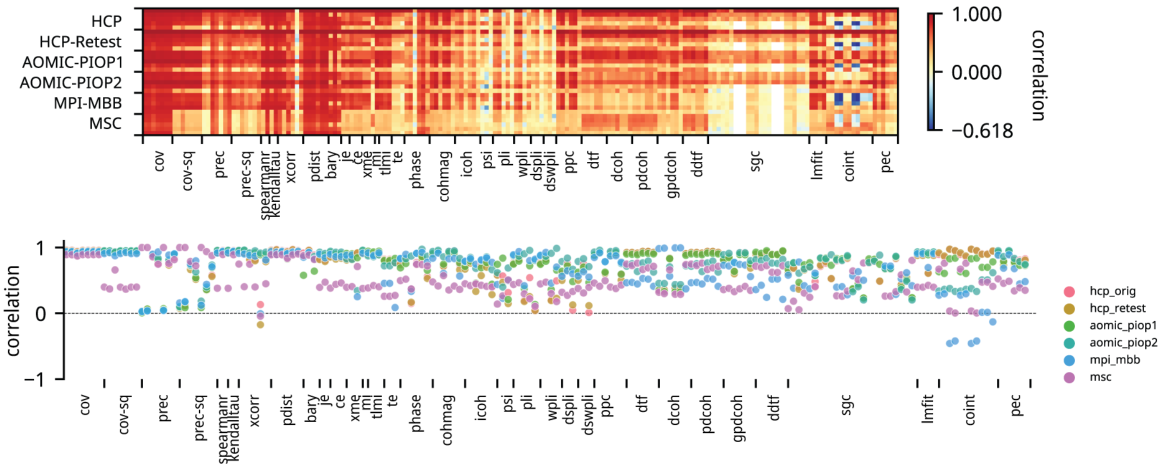

Supplementary Figure 19 | **Variability of pairwise statistics across datasets** (Top) Individual FC from each of the 6 datasets (e.g. FC_HCP) is compared to FC from the other 5 datasets (e.g. FC_HCP–Retest, FC_AOMIC–PIOP1, FC_AOMIC–PIOP2, FC_MPI–MBB, FC_MSC). (Bottom) Individual FC from each of the 6 datasets (e.g. FC_HCP) is compared to the mean FC across the 6 datasets (average of FC_HCP, FC_HCP–Retest, FC_AOMIC–PIOP1, FC_AOMIC–PIOP2, FC_MPI–MBB, FC_MSC). Both comparisons are carried out using Spearman's rank correlation on the upper triangular elements. This is done for each pairwise interaction statistic (x-axis).

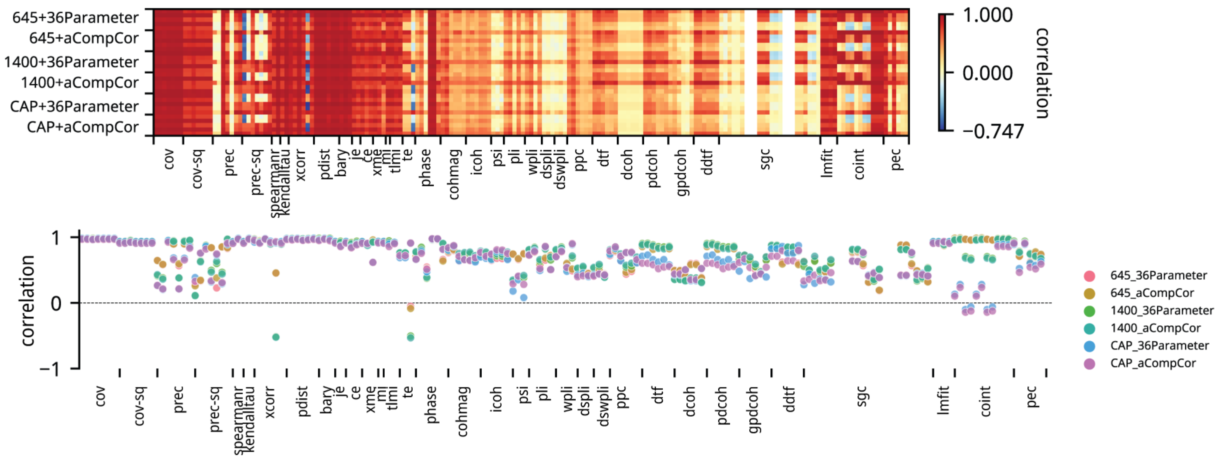

Supplementary Figure 20 | **Variability of pairwise statistics across acquisition and motion correction conditions using the RBC–NKI dataset** (Top) Individual FC from each of the 6 acquisition and motion correction combinations (e.g. FC_645+36Parameter) is compared to FC from the other 5 datasets (e.g. FC_645+aCompCor, FC_1400+36Parameter, FC_1400+aCompCor, FC_CAP+36Parameter, FC_CAP+aCompCor). (Bottom) Individual FC from each of the 6 acquisition and motion correction combinations (e.g. FC_645+36Parameter) is compared to the mean FC across the 6 datasets (average of FC_645+36Parameter, FC_645+aCompCor, FC_1400+36Parameter, FC_1400+aCompCor, FC_CAP+36Parameter, FC_CAP+aCompCor). Both comparisons are carried out using Spearman's rank correlation on the upper triangular elements. This is done for each pairwise interaction statistic (x-axis).

Supplementary Table 1: | **Full list of 239 *pyspi* statistics** The 239 pairwise statistics grouped into 49 measures across 6 major model families used in the main results.

Index	Statistic	Measure	Description	Category
1	cov_EmpiricalCovariance	cov	Covariance	Basic statistics
2	cov_EllipticEnvelope	cov	Covariance	Basic statistics
3	cov_GraphicalLassoCV	cov	Covariance	Basic statistics
4	cov_LedoitWolf	cov	Covariance	Basic statistics
5	cov_MinCovDet	cov	Covariance	Basic statistics
6	cov_OAS	cov	Covariance	Basic statistics
7	cov_ShrunkCovariance	cov	Covariance	Basic statistics
8	cov-sq_EmpiricalCovariance	cov-sq	Covariance	Basic statistics
9	cov-sq_EllipticEnvelope	cov-sq	Covariance	Basic statistics
10	cov-sq_GraphicalLassoCV	cov-sq	Covariance	Basic statistics
11	cov-sq_LedoitWolf	cov-sq	Covariance	Basic statistics
12	cov-sq_MinCovDet	cov-sq	Covariance	Basic statistics
13	cov-sq_OAS	cov-sq	Covariance	Basic statistics
14	cov-sq_ShrunkCovariance	cov-sq	Covariance	Basic statistics
15	prec_EmpiricalCovariance	prec	Precision	Basic statistics
16	prec_EllipticEnvelope	prec	Precision	Basic statistics
17	prec_GraphicalLassoCV	prec	Precision	Basic statistics
18	prec_LedoitWolf	prec	Precision	Basic statistics
19	prec_MinCovDet	prec	Precision	Basic statistics
20	prec_OAS	prec	Precision	Basic statistics
21	prec_ShrunkCovariance	prec	Precision	Basic statistics
22	prec-sq_EmpiricalCovariance	prec-sq	Precision	Basic statistics
23	prec-sq_EllipticEnvelope	prec-sq	Precision	Basic statistics
24	prec-sq_GraphicalLassoCV	prec-sq	Precision	Basic statistics
25	prec-sq_LedoitWolf	prec-sq	Precision	Basic statistics
26	prec-sq_MinCovDet	prec-sq	Precision	Basic statistics
27	prec-sq_OAS	prec-sq	Precision	Basic statistics
28	prec-sq_ShrunkCovariance	prec-sq	Precision	Basic statistics
29	spearmanr-sq	spearmanr	Spearman's rank-correlation coefficient	Basic statistics
30	spearmanr	spearmanr	Spearman's rank-correlation coefficient	Basic statistics
31	kendalltau-sq	kendalltau	Kendall's rank-correlation coefficient	Basic statistics
32	kendalltau	kendalltau	Kendall's rank-correlation coefficient	Basic statistics
33	xcorr_max_sig-True	xcorr	Cross correlation	Basic statistics
34	xcorr-sq_max_sig-True	xcorr	Cross correlation	Basic statistics
35	xcorr_mean_sig-True	xcorr	Cross correlation	Basic statistics
36	xcorr-sq_mean_sig-True	xcorr	Cross correlation	Basic statistics
37	xcorr_mean_sig-False	xcorr	Cross correlation	Basic statistics
38	xcorr-sq_mean_sig-False	xcorr	Cross correlation	Basic statistics
39	pdist_euclidean	pdist	Pairwise distance	Distance similarity
40	pdist_cityblock	pdist	Pairwise distance	Distance similarity
41	pdist_cosine	pdist	Pairwise distance	Distance similarity
42	pdist_chebyshev	pdist	Pairwise distance	Distance similarity
43	pdist_canberra	pdist	Pairwise distance	Distance similarity
44	pdist_braycurtis	pdist	Pairwise distance	Distance similarity
45	dcorr	dcorr	Distance correlation	Distance similarity
46	dcorr_biased	dcorr	Distance correlation	Distance similarity
47	hsic	hsic	Hilbert-Schmidt Independence Criterion	Distance similarity
48	hsic_biased	hsic	Hilbert-Schmidt Independence Criterion	Distance similarity
49	dcorrx_maxlag-1	dcorrx	Cross distance correlation	Distance similarity
50	dtw	dtw	Dynamic time warping	Distance similarity
51	dtw_constraint-itakura	dtw	Dynamic time warping	Distance similarity
52	dtw_constraint-sakoe-chiba	dtw	Dynamic time warping	Distance similarity
53	softdtw	softdtw	Soft dynamic time warping	Distance similarity
54	softdtw_constraint-itakura	softdtw	Soft dynamic time warping	Distance similarity
55	softdtw_constraint-sakoe-chiba	softdtw	Soft dynamic time warping	Distance similarity
56	lcss	lcss	Longest common subsequence	Distance similarity
57	lcss_constraint-itakura	lcss	Longest common subsequence	Distance similarity
58	lcss_constraint-sakoe-chiba	lcss	Longest common subsequence	Distance similarity
59	bary_euclidean_max	bary	Barycenter	Distance similarity

60	bary_dtw_mean	bary	Barycenter	Distance similarity
61	bary_dtw_max	bary	Barycenter	Distance similarity
62	bary_sgddtw_mean	bary	Barycenter	Distance similarity
63	bary_sgddtw_max	bary	Barycenter	Distance similarity
64	bary-sq_euclidean_mean	bary	Barycenter	Distance similarity
65	bary-sq_euclidean_max	bary	Barycenter	Distance similarity
66	bary-sq_dtw_mean	bary	Barycenter	Distance similarity
67	bary-sq_dtw_max	bary	Barycenter	Distance similarity
68	bary-sq_sgddtw_mean	bary	Barycenter	Distance similarity
69	bary-sq_sgddtw_max	bary	Barycenter	Distance similarity
70	anm	anm	Additive noise model	Causal inference
71	cds	cds	Conditional distribution similarity fit	Causal inference
72	reci	reci	Regression error-based causal inference	Causal inference
73	igci	igci	Information-geometric causal inference	Causal inference
74	je_gaussian	je	Joint entropy	Information theory
75	je_kozachenko	je	Joint entropy	Information theory
76	je_kernel_W-0.5	je	Joint entropy	Information theory
77	ce_gaussian	ce	Conditional entropy	Information theory
78	ce_kozachenko	ce	Conditional entropy	Information theory
79	ce_kernel_W-0.5	ce	Conditional entropy	Information theory
80	cce_gaussian	cce	Causally conditioned entropy	Information theory
81	cce_kernel_W-0.5	cce	Causally conditioned entropy	Information theory
82	xme_gaussian_k1	xme	Cross-map entropy	Information theory
83	xme_kozachenko_k1	xme	Cross-map entropy	Information theory
84	xme_kernel_W-0.5_k1	xme	Cross-map entropy	Information theory
85	xme_gaussian_k10	xme	Cross-map entropy	Information theory
86	xme_kernel_W-0.5_k10	xme	Cross-map entropy	Information theory
87	di_gaussian	di	Directed information	Information theory
88	di_kernel_W-0.5	di	Directed information	Information theory
89	si_gaussian_k-1	si	Stochastic interaction	Information theory
90	si_kernel_W-0.5_k-1	si	Stochastic interaction	Information theory
91	mi_gaussian	mi	Mutual information	Information theory
92	mi_kraskov_NN-4	mi	Mutual information	Information theory
93	mi_kraskov_NN-4_DCE	mi	Mutual information	Information theory
94	mi_kernel_W-0.25	mi	Mutual information	Information theory
95	tlmi_gaussian	tlmi	Time-lagged mutual information	Information theory
96	tlmi_kraskov_NN-4	tlmi	Time-lagged mutual information	Information theory
97	tlmi_kraskov_NN-4_DCE	tlmi	Time-lagged mutual information	Information theory
98	tlmi_kernel_W-0.25	tlmi	Time-lagged mutual information	Information theory
99	te_kraskov_NN-4_DCE_k-2_kt-1_l-1_lt-1	te	Transfer entropy	Information theory
100	te_kraskov_NN-4_DCE_k-1_kt-1_l-1_lt-1	te	Transfer entropy	Information theory
101	te_kraskov_NN-4_k-1_kt-1_l-1_lt-1	te	Transfer entropy	Information theory
102	te_kernel_W-0.25_k-1	te	Transfer entropy	Information theory
103	gc_gaussian_k-max-10_tau-max-2	te	Transfer entropy	Information theory
104	gc_gaussian_k-1_kt-1_l-1_lt-1	te	Transfer entropy	Information theory
105	te_symbolic_k-1_kt-1_l-1_lt-1	te	Transfer entropy	Information theory
106	te_symbolic_k-10_kt-1_l-1_lt-1	te	Transfer entropy	Information theory
107	phi_star_t-1_norm-0	phi	Integrated information	Information theory
108	phi_star_t-1_norm-1	phi	Integrated information	Information theory
109	phi_Geo_t-1_norm-0	phi	Integrated information	Information theory
110	phi_Geo_t-1_norm-1	phi	Integrated information	Information theory
111	phase_multitaper_mean_fs-1_fmin-0_fmax-0-5	phase	Coherence phase	Spectral
112	phase_multitaper_mean_fs-1_fmin-0_fmax-0-25	phase	Coherence phase	Spectral
113	phase_multitaper_mean_fs-1_fmin-0-25_fmax-0-5	phase	Coherence phase	Spectral
114	phase_multitaper_max_fs-1_fmin-0_fmax-0-5	phase	Coherence phase	Spectral

115	phase_multitaper_max_fs-1_fmin-0_fmax-0-25	phase	Coherence phase	Spectral
116	phase_multitaper_max_fs-1_fmin-0-25_fmax-0-5	phase	Coherence phase	Spectral
117	cohmag_multitaper_mean_fs-1_fmin-0_fmax-0-5	cohmag	Coherence magnitude	Spectral
118	cohmag_multitaper_mean_fs-1_fmin-0_fmax-0-25	cohmag	Coherence magnitude	Spectral
119	cohmag_multitaper_mean_fs-1_fmin-0-25_fmax-0-5	cohmag	Coherence magnitude	Spectral
120	cohmag_multitaper_max_fs-1_fmin-0_fmax-0-5	cohmag	Coherence magnitude	Spectral
121	cohmag_multitaper_max_fs-1_fmin-0_fmax-0-25	cohmag	Coherence magnitude	Spectral
122	cohmag_multitaper_max_fs-1_fmin-0-25_fmax-0-5	cohmag	Coherence magnitude	Spectral
123	icoh_multitaper_mean_fs-1_fmin-0_fmax-0-5	icoh	Imaginary coherence	Spectral
124	icoh_multitaper_mean_fs-1_fmin-0_fmax-0-25	icoh	Imaginary coherence	Spectral
125	icoh_multitaper_mean_fs-1_fmin-0-25_fmax-0-5	icoh	Imaginary coherence	Spectral
126	icoh_multitaper_max_fs-1_fmin-0_fmax-0-5	icoh	Imaginary coherence	Spectral
127	icoh_multitaper_max_fs-1_fmin-0_fmax-0-25	icoh	Imaginary coherence	Spectral
128	icoh_multitaper_max_fs-1_fmin-0-25_fmax-0-5	icoh	Imaginary coherence	Spectral
129	psi_multitaper_mean_fs-1_fmin-0_fmax-0-5	psi	Phase slope index	Spectral
130	psi_multitaper_mean_fs-1_fmin-0_fmax-0-25	psi	Phase slope index	Spectral
131	psi_multitaper_mean_fs-1_fmin-0-25_fmax-0-5	psi	Phase slope index	Spectral
132	plv_multitaper_mean_fs-1_fmin-0_fmax-0-5	plv	Phase locking value	Spectral
133	plv_multitaper_mean_fs-1_fmin-0_fmax-0-25	plv	Phase locking value	Spectral
134	plv_multitaper_mean_fs-1_fmin-0-25_fmax-0-5	plv	Phase locking value	Spectral
135	plv_multitaper_max_fs-1_fmin-0_fmax-0-5	plv	Phase locking value	Spectral
136	plv_multitaper_max_fs-1_fmin-0_fmax-0-25	plv	Phase locking value	Spectral
137	plv_multitaper_max_fs-1_fmin-0-25_fmax-0-5	plv	Phase locking value	Spectral
138	pli_multitaper_mean_fs-1_fmin-0_fmax-0-5	pli	Phase lag index	Spectral
139	pli_multitaper_mean_fs-1_fmin-0_fmax-0-25	pli	Phase lag index	Spectral
140	pli_multitaper_mean_fs-1_fmin-0-25_fmax-0-5	pli	Phase lag index	Spectral
141	pli_multitaper_max_fs-1_fmin-0_fmax-0-25	pli	Phase lag index	Spectral
142	pli_multitaper_max_fs-1_fmin-0-25_fmax-0-5	pli	Phase lag index	Spectral
143	wpli_multitaper_mean_fs-1_fmin-0_fmax-0-5	wpli	Weighted phase lag index	Spectral
144	wpli_multitaper_mean_fs-1_fmin-0_fmax-0-25	wpli	Weighted phase lag index	Spectral
145	wpli_multitaper_mean_fs-1_fmin-0-25_fmax-0-5	wpli	Weighted phase lag index	Spectral
146	wpli_multitaper_max_fs-1_fmin-0_fmax-0-25	wpli	Weighted phase lag index	Spectral

147	dspli_multitaper_mean_fs-1_fmin-0_fmax-0-5	dspli	Debiased squared phase lag index	Spectral
148	dspli_multitaper_mean_fs-1_fmin-0_fmax-0-25	dspli	Debiased squared phase lag index	Spectral
149	dspli_multitaper_mean_fs-1_fmin-0-25_fmax-0-5	dspli	Debiased squared phase lag index	Spectral
150	dswpli_multitaper_mean_fs-1_fmin-0_fmax-0-5	dswpli	Debiased squared weighted phase lag index	Spectral
151	dswpli_multitaper_mean_fs-1_fmin-0_fmax-0-25	dswpli	Debiased squared weighted phase lag index	Spectral
152	dswpli_multitaper_mean_fs-1_fmin-0-25_fmax-0-5	dswpli	Debiased squared weighted phase lag index	Spectral
153	ppc_multitaper_mean_fs-1_fmin-0_fmax-0-5	ppc	Pairwise phase consistency	Spectral
154	ppc_multitaper_mean_fs-1_fmin-0_fmax-0-25	ppc	Pairwise phase consistency	Spectral
155	ppc_multitaper_mean_fs-1_fmin-0-25_fmax-0-5	ppc	Pairwise phase consistency	Spectral
156	ppc_multitaper_max_fs-1_fmin-0_fmax-0-5	ppc	Pairwise phase consistency	Spectral
157	ppc_multitaper_max_fs-1_fmin-0_fmax-0-25	ppc	Pairwise phase consistency	Spectral
158	ppc_multitaper_max_fs-1_fmin-0-25_fmax-0-5	ppc	Pairwise phase consistency	Spectral
159	dtf_multitaper_mean_fs-1_fmin-0_fmax-0-5	dtf	Directed transfer function	Spectral
160	dtf_multitaper_mean_fs-1_fmin-0_fmax-0-25	dtf	Directed transfer function	Spectral
161	dtf_multitaper_mean_fs-1_fmin-0-25_fmax-0-5	dtf	Directed transfer function	Spectral
162	dtf_multitaper_max_fs-1_fmin-0_fmax-0-5	dtf	Directed transfer function	Spectral
163	dtf_multitaper_max_fs-1_fmin-0_fmax-0-25	dtf	Directed transfer function	Spectral
164	dtf_multitaper_max_fs-1_fmin-0-25_fmax-0-5	dtf	Directed transfer function	Spectral
165	dcoh_multitaper_mean_fs-1_fmin-0_fmax-0-5	dcoh	Directed coherence	Spectral
166	dcoh_multitaper_mean_fs-1_fmin-0_fmax-0-25	dcoh	Directed coherence	Spectral
167	dcoh_multitaper_mean_fs-1_fmin-0-25_fmax-0-5	dcoh	Directed coherence	Spectral
168	dcoh_multitaper_max_fs-1_fmin-0_fmax-0-5	dcoh	Directed coherence	Spectral
169	dcoh_multitaper_max_fs-1_fmin-0_fmax-0-25	dcoh	Directed coherence	Spectral
170	dcoh_multitaper_max_fs-1_fmin-0-25_fmax-0-5	dcoh	Directed coherence	Spectral
171	pdcoh_multitaper_mean_fs-1_fmin-0_fmax-0-5	pdcoh	Partial directed coherence	Spectral
172	pdcoh_multitaper_mean_fs-1_fmin-0_fmax-0-25	pdcoh	Partial directed coherence	Spectral
173	pdcoh_multitaper_mean_fs-1_fmin-0-25_fmax-0-5	pdcoh	Partial directed coherence	Spectral
174	pdcoh_multitaper_max_fs-1_fmin-0_fmax-0-5	pdcoh	Partial directed coherence	Spectral
175	pdcoh_multitaper_max_fs-1_fmin-0_fmax-0-25	pdcoh	Partial directed coherence	Spectral
176	pdcoh_multitaper_max_fs-1_fmin-0-25_fmax-0-5	pdcoh	Partial directed coherence	Spectral
177	gpdcoh_multitaper_mean_fs-1_fmin-0_fmax-0-5	gpdcoh	Generalized partial directed coherence	Spectral
178	gpdcoh_multitaper_mean_fs-1_fmin-0_fmax-0-25	gpdcoh	Generalized partial directed coherence	Spectral

179	gpdcoh_multitaper_mean_fs-1_fmin-0-25_fmax-0-5	gpdcoh	Generalized partial directed coherence	Spectral
180	gpdcoh_multitaper_max_fs-1_fmin-0_fmax-0-5	gpdcoh	Generalized partial directed coherence	Spectral
181	gpdcoh_multitaper_max_fs-1_fmin-0_fmax-0-25	gpdcoh	Generalized partial directed coherence	Spectral
182	gpdcoh_multitaper_max_fs-1_fmin-0-25_fmax-0-5	gpdcoh	Generalized partial directed coherence	Spectral
183	ddtf_multitaper_mean_fs-1_fmin-0_fmax-0-5	ddtf	Direct directed transfer function	Spectral
184	ddtf_multitaper_mean_fs-1_fmin-0_fmax-0-25	ddtf	Direct directed transfer function	Spectral
185	ddtf_multitaper_mean_fs-1_fmin-0-25_fmax-0-5	ddtf	Direct directed transfer function	Spectral
186	ddtf_multitaper_max_fs-1_fmin-0_fmax-0-5	ddtf	Direct directed transfer function	Spectral
187	ddtf_multitaper_max_fs-1_fmin-0_fmax-0-25	ddtf	Direct directed transfer function	Spectral
188	ddtf_multitaper_max_fs-1_fmin-0-25_fmax-0-5	ddtf	Direct directed transfer function	Spectral
189	sgc_nonparametric_mean_fs-1_fmin-0_fmax-0-5	sgc	Spectral Granger causality	Spectral
190	sgc_nonparametric_mean_fs-1_fmin-0_fmax-0-25	sgc	Spectral Granger causality	Spectral
191	sgc_nonparametric_mean_fs-1_fmin-0-25_fmax-0-5	sgc	Spectral Granger causality	Spectral
192	sgc_nonparametric_max_fs-1_fmin-0_fmax-0-5	sgc	Spectral Granger causality	Spectral
193	sgc_nonparametric_max_fs-1_fmin-0_fmax-0-25	sgc	Spectral Granger causality	Spectral
194	sgc_nonparametric_max_fs-1_fmin-0-25_fmax-0-5	sgc	Spectral Granger causality	Spectral
195	sgc_parametric_mean_fs-1_fmin-0_fmax-0-5_order-None	sgc	Spectral Granger causality	Spectral
196	sgc_parametric_mean_fs-1_fmin-0_fmax-0-25_order-None	sgc	Spectral Granger causality	Spectral
197	sgc_parametric_mean_fs-1_fmin-0-25_fmax-0-5_order-None	sgc	Spectral Granger causality	Spectral
198	sgc_parametric_mean_fs-1_fmin-1e-05_fmax-0-5_order-1	sgc	Spectral Granger causality	Spectral
199	sgc_parametric_mean_fs-1_fmin-0_fmax-0-25_order-1	sgc	Spectral Granger causality	Spectral
200	sgc_parametric_mean_fs-1_fmin-0-25_fmax-0-5_order-1	sgc	Spectral Granger causality	Spectral
201	sgc_parametric_mean_fs-1_fmin-1e-05_fmax-0-5_order-20	sgc	Spectral Granger causality	Spectral
202	sgc_parametric_mean_fs-1_fmin-0_fmax-0-25_order-20	sgc	Spectral Granger causality	Spectral
203	sgc_parametric_mean_fs-1_fmin-0-25_fmax-0-5_order-20	sgc	Spectral Granger causality	Spectral
204	sgc_parametric_max_fs-1_fmin-1e-05_fmax-0-5_order-None	sgc	Spectral Granger causality	Spectral
205	sgc_parametric_max_fs-1_fmin-0_fmax-0-25_order-None	sgc	Spectral Granger causality	Spectral
206	sgc_parametric_max_fs-1_fmin-0-25_fmax-0-5_order-None	sgc	Spectral Granger causality	Spectral
207	sgc_parametric_max_fs-1_fmin-1e-05_fmax-0-5_order-1	sgc	Spectral Granger causality	Spectral
208	sgc_parametric_max_fs-1_fmin-0_fmax-0-25_order-1	sgc	Spectral Granger causality	Spectral

209	sgc_parametric_max_fs-1_fmin-0-25_fmax-0-5_order-1	sgc	Spectral Granger causality	Spectral
210	sgc_parametric_max_fs-1_fmin-1e-05_fmax-0-5_order-20	sgc	Spectral Granger causality	Spectral
211	sgc_parametric_max_fs-1_fmin-0_fmax-0-25_order-20	sgc	Spectral Granger causality	Spectral
212	sgc_parametric_max_fs-1_fmin-0-25_fmax-0-5_order-20	sgc	Spectral Granger causality	Spectral
213	psi_wavelet_mean_fs-1_fmin-0_fmax-0-5_mean	psi-wavelet	Phase slope index (wavelet)	Spectral
214	psi_wavelet_mean_fs-1_fmin-0_fmax-0-25_mean	psi-wavelet	Phase slope index (wavelet)	Spectral
215	psi_wavelet_mean_fs-1_fmin-0-25_fmax-0-5_mean	psi-wavelet	Phase slope index (wavelet)	Spectral
216	psi_wavelet_max_fs-1_fmin-0_fmax-0-5_max	psi-wavelet	Phase slope index (wavelet)	Spectral
217	psi_wavelet_max_fs-1_fmin-0_fmax-0-25_max	psi-wavelet	Phase slope index (wavelet)	Spectral
218	psi_wavelet_max_fs-1_fmin-0-25_fmax-0-5_max	psi-wavelet	Phase slope index (wavelet)	Spectral
219	lmfit_Ridge	lmfit	Linear model fit	Miscellaneous
220	lmfit_SGDRegressor	lmfit	Linear model fit	Miscellaneous
221	lmfit_ElasticNet	lmfit	Linear model fit	Miscellaneous
222	lmfit_BayesianRidge	lmfit	Linear model fit	Miscellaneous
223	coint_johansen_max_eig_stat_order-0_ardiff-10	coint	Cointegration	Miscellaneous
224	coint_johansen_trace_stat_order-0_ardiff-10	coint	Cointegration	Miscellaneous
225	coint_johansen_max_eig_stat_order-0_ardiff-1	coint	Cointegration	Miscellaneous
226	coint_johansen_trace_stat_order-0_ardiff-1	coint	Cointegration	Miscellaneous
227	coint_johansen_max_eig_stat_order-1_ardiff-10	coint	Cointegration	Miscellaneous
228	coint_johansen_trace_stat_order-1_ardiff-10	coint	Cointegration	Miscellaneous
229	coint_johansen_max_eig_stat_order-1_ardiff-1	coint	Cointegration	Miscellaneous
230	coint_johansen_trace_stat_order-1_ardiff-1	coint	Cointegration	Miscellaneous
231	coint_aeg_tstat_trend-c_autolag-aic_maxlag-10	coint	Cointegration	Miscellaneous
232	coint_aeg_tstat_trend-ct_autolag-aic_maxlag-10	coint	Cointegration	Miscellaneous
233	coint_aeg_tstat_trend-ct_autolag-bic_maxlag-10	coint	Cointegration	Miscellaneous
234	pec	pec	Power envelope correlation	Miscellaneous
235	pec_orth	pec	Power envelope correlation	Miscellaneous
236	pec_log	pec	Power envelope correlation	Miscellaneous
237	pec_orth_log	pec	Power envelope correlation	Miscellaneous
238	pec_orth_abs	pec	Power envelope correlation	Miscellaneous
239	pec_orth_log_abs	pec	Power envelope correlation	Miscellaneous

Supplementary Table 2: | **Reduced list of 179 *pyspi* statistics** The subset of 179 pairwise statistics used in the calculation of Fig. 6c.

Index	Statistic	Measure	Description	Category
1	cov_EmpiricalCovariance	cov	Covariance	Basic statistics
2	cov_EllipticEnvelope	cov	Covariance	Basic statistics
3	cov_GraphicalLassoCV	cov	Covariance	Basic statistics
4	cov_LedoitWolf	cov	Covariance	Basic statistics
5	cov_MinCovDet	cov	Covariance	Basic statistics
6	cov_OAS	cov	Covariance	Basic statistics
7	cov_ShrunkCovariance	cov	Covariance	Basic statistics
8	cov-sq_EmpiricalCovariance	cov-sq	Covariance	Basic statistics
9	cov-sq_EllipticEnvelope	cov-sq	Covariance	Basic statistics
10	cov-sq_GraphicalLassoCV	cov-sq	Covariance	Basic statistics
11	cov-sq_LedoitWolf	cov-sq	Covariance	Basic statistics
12	cov-sq_MinCovDet	cov-sq	Covariance	Basic statistics
13	cov-sq_OAS	cov-sq	Covariance	Basic statistics
14	cov-sq_ShrunkCovariance	cov-sq	Covariance	Basic statistics
15	prec_EmpiricalCovariance	prec	Precision	Basic statistics
16	prec_EllipticEnvelope	prec	Precision	Basic statistics
17	prec_GraphicalLassoCV	prec	Precision	Basic statistics
18	prec_LedoitWolf	prec	Precision	Basic statistics
19	prec_MinCovDet	prec	Precision	Basic statistics
20	prec_OAS	prec	Precision	Basic statistics
21	prec_ShrunkCovariance	prec	Precision	Basic statistics
22	prec-sq_EmpiricalCovariance	prec-sq	Precision	Basic statistics
23	prec-sq_EllipticEnvelope	prec-sq	Precision	Basic statistics
24	prec-sq_GraphicalLassoCV	prec-sq	Precision	Basic statistics
25	prec-sq_LedoitWolf	prec-sq	Precision	Basic statistics
26	prec-sq_MinCovDet	prec-sq	Precision	Basic statistics
27	prec-sq_OAS	prec-sq	Precision	Basic statistics
28	prec-sq_ShrunkCovariance	prec-sq	Precision	Basic statistics
29	spearmanr-sq	spearmanr	Spearman's rank-correlation coefficient	Basic statistics
30	spearmanr	spearmanr	Spearman's rank-correlation coefficient	Basic statistics
31	kendalltau-sq	kendalltau	Kendall's rank-correlation coefficient	Basic statistics
32	kendalltau	kendalltau	Kendall's rank-correlation coefficient	Basic statistics
33	xcorr_max_sig-True	xcorr	Cross correlation	Basic statistics
34	xcorr-sq_max_sig-True	xcorr	Cross correlation	Basic statistics
35	xcorr_mean_sig-True	xcorr	Cross correlation	Basic statistics
36	xcorr-sq_mean_sig-True	xcorr	Cross correlation	Basic statistics
37	xcorr_mean_sig-False	xcorr	Cross correlation	Basic statistics
38	xcorr-sq_mean_sig-False	xcorr	Cross correlation	Basic statistics
39	pdist_euclidean	pdist	Pairwise distance	Distance similarity
40	pdist_cityblock	pdist	Pairwise distance	Distance similarity
41	pdist_cosine	pdist	Pairwise distance	Distance similarity
42	pdist_chebyshev	pdist	Pairwise distance	Distance similarity
43	pdist_canberra	pdist	Pairwise distance	Distance similarity
44	pdist_braycurtis	pdist	Pairwise distance	Distance similarity
45	bary_euclidean_max	bary	Barycenter	Distance similarity
46	bary-sq_euclidean_mean	bary	Barycenter	Distance similarity
47	bary-sq_euclidean_max	bary	Barycenter	Distance similarity
48	je_gaussian	je	Joint entropy	Information theory
49	je_kernel_W-0.5	je	Joint entropy	Information theory
50	ce_gaussian	ce	Conditional entropy	Information theory
51	ce_kozachenko	ce	Conditional entropy	Information theory
52	ce_kernel_W-0.5	ce	Conditional entropy	Information theory
53	xme_gaussian_k1	xme	Cross-map entropy	Information theory
54	xme_gaussian_k10	xme	Cross-map entropy	Information theory
55	xme_kernel_W-0.5_k10	xme	Cross-map entropy	Information theory
56	mi_gaussian	mi	Mutual information	Information theory
57	tlmi_gaussian	tlmi	Time-lagged mutual information	Information theory
58	tlmi_kraskov_NN-4	tlmi	Time-lagged mutual information	Information theory
59	tlmi_kraskov_NN-4_DCE	tlmi	Time-lagged mutual information	Information theory
60	gc_gaussian_k-1_kt-1_l-1_lt-1	te	Transfer entropy	Information theory

61	te_symbolic_k-1_kt-1_l-1_lt-1	te	Transfer entropy	Information theory
62	te_symbolic_k-10_kt-1_l-1_lt-1	te	Transfer entropy	Information theory
63	phase_multitaper_mean_fs-1_fmin-0_fmax-0-5	phase	Coherence phase	Spectral
64	phase_multitaper_mean_fs-1_fmin-0_fmax-0-25	phase	Coherence phase	Spectral
65	phase_multitaper_mean_fs-1_fmin-0-25_fmax-0-5	phase	Coherence phase	Spectral
66	phase_multitaper_max_fs-1_fmin-0_fmax-0-5	phase	Coherence phase	Spectral
67	phase_multitaper_max_fs-1_fmin-0_fmax-0-25	phase	Coherence phase	Spectral
68	phase_multitaper_max_fs-1_fmin-0-25_fmax-0-5	phase	Coherence phase	Spectral
69	cohmag_multitaper_mean_fs-1_fmin-0_fmax-0-5	cohmag	Coherence magnitude	Spectral
70	cohmag_multitaper_mean_fs-1_fmin-0_fmax-0-25	cohmag	Coherence magnitude	Spectral
71	cohmag_multitaper_mean_fs-1_fmin-0-25_fmax-0-5	cohmag	Coherence magnitude	Spectral
72	cohmag_multitaper_max_fs-1_fmin-0_fmax-0-5	cohmag	Coherence magnitude	Spectral
73	cohmag_multitaper_max_fs-1_fmin-0_fmax-0-25	cohmag	Coherence magnitude	Spectral
74	cohmag_multitaper_max_fs-1_fmin-0-25_fmax-0-5	cohmag	Coherence magnitude	Spectral
75	icoh_multitaper_mean_fs-1_fmin-0_fmax-0-5	icoh	Imaginary coherence	Spectral
76	icoh_multitaper_mean_fs-1_fmin-0_fmax-0-25	icoh	Imaginary coherence	Spectral
77	icoh_multitaper_mean_fs-1_fmin-0-25_fmax-0-5	icoh	Imaginary coherence	Spectral
78	icoh_multitaper_max_fs-1_fmin-0_fmax-0-5	icoh	Imaginary coherence	Spectral
79	icoh_multitaper_max_fs-1_fmin-0_fmax-0-25	icoh	Imaginary coherence	Spectral
80	icoh_multitaper_max_fs-1_fmin-0-25_fmax-0-5	icoh	Imaginary coherence	Spectral
81	psi_multitaper_mean_fs-1_fmin-0_fmax-0-5	psi	Phase slope index	Spectral
82	psi_multitaper_mean_fs-1_fmin-0_fmax-0-25	psi	Phase slope index	Spectral
83	psi_multitaper_mean_fs-1_fmin-0-25_fmax-0-5	psi	Phase slope index	Spectral
84	pli_multitaper_mean_fs-1_fmin-0_fmax-0-5	pli	Phase lag index	Spectral
85	pli_multitaper_mean_fs-1_fmin-0_fmax-0-25	pli	Phase lag index	Spectral
86	pli_multitaper_mean_fs-1_fmin-0-25_fmax-0-5	pli	Phase lag index	Spectral
87	pli_multitaper_max_fs-1_fmin-0_fmax-0-25	pli	Phase lag index	Spectral
88	pli_multitaper_max_fs-1_fmin-0-25_fmax-0-5	pli	Phase lag index	Spectral
89	wpli_multitaper_mean_fs-1_fmin-0_fmax-0-5	wpli	Weighted phase lag index	Spectral
90	wpli_multitaper_mean_fs-1_fmin-0_fmax-0-25	wpli	Weighted phase lag index	Spectral
91	wpli_multitaper_mean_fs-1_fmin-0-25_fmax-0-5	wpli	Weighted phase lag index	Spectral
92	wpli_multitaper_max_fs-1_fmin-0-25_fmax-0-5	wpli	Weighted phase lag index	Spectral
93	dspli_multitaper_mean_fs-1_fmin-0_fmax-0-5	dspli	Debiased squared phase lag index	Spectral

94	dspli_multitaper_mean_fs-1_fmin-0_fmax-0-25	dspli	Debiased squared phase lag index	Spectral
95	dspli_multitaper_mean_fs-1_fmin-0-25_fmax-0-5	dspli	Debiased squared phase lag index	Spectral
96	dswpli_multitaper_mean_fs-1_fmin-0_fmax-0-5	dswpli	Debiased squared weighted phase lag index	Spectral
97	dswpli_multitaper_mean_fs-1_fmin-0_fmax-0-25	dswpli	Debiased squared weighted phase lag index	Spectral
98	dswpli_multitaper_mean_fs-1_fmin-0-25_fmax-0-5	dswpli	Debiased squared weighted phase lag index	Spectral
99	ppc_multitaper_mean_fs-1_fmin-0_fmax-0-5	ppc	Pairwise phase consistency	Spectral
100	ppc_multitaper_mean_fs-1_fmin-0_fmax-0-25	ppc	Pairwise phase consistency	Spectral
101	ppc_multitaper_mean_fs-1_fmin-0-25_fmax-0-5	ppc	Pairwise phase consistency	Spectral
102	ppc_multitaper_max_fs-1_fmin-0_fmax-0-5	ppc	Pairwise phase consistency	Spectral
103	ppc_multitaper_max_fs-1_fmin-0_fmax-0-25	ppc	Pairwise phase consistency	Spectral
104	ppc_multitaper_max_fs-1_fmin-0-25_fmax-0-5	ppc	Pairwise phase consistency	Spectral
105	dtf_multitaper_mean_fs-1_fmin-0_fmax-0-5	dtf	Directed transfer function	Spectral
106	dtf_multitaper_mean_fs-1_fmin-0_fmax-0-25	dtf	Directed transfer function	Spectral
107	dtf_multitaper_mean_fs-1_fmin-0-25_fmax-0-5	dtf	Directed transfer function	Spectral
108	dtf_multitaper_max_fs-1_fmin-0_fmax-0-5	dtf	Directed transfer function	Spectral
109	dtf_multitaper_max_fs-1_fmin-0_fmax-0-25	dtf	Directed transfer function	Spectral
110	dtf_multitaper_max_fs-1_fmin-0-25_fmax-0-5	dtf	Directed transfer function	Spectral
111	dcoh_multitaper_mean_fs-1_fmin-0_fmax-0-5	dcoh	Directed coherence	Spectral
112	dcoh_multitaper_mean_fs-1_fmin-0_fmax-0-25	dcoh	Directed coherence	Spectral
113	dcoh_multitaper_mean_fs-1_fmin-0-25_fmax-0-5	dcoh	Directed coherence	Spectral
114	dcoh_multitaper_max_fs-1_fmin-0_fmax-0-5	dcoh	Directed coherence	Spectral
115	dcoh_multitaper_max_fs-1_fmin-0_fmax-0-25	dcoh	Directed coherence	Spectral
116	dcoh_multitaper_max_fs-1_fmin-0-25_fmax-0-5	dcoh	Directed coherence	Spectral
117	pdcoh_multitaper_mean_fs-1_fmin-0_fmax-0-5	pdcoh	Partial directed coherence	Spectral
118	pdcoh_multitaper_mean_fs-1_fmin-0_fmax-0-25	pdcoh	Partial directed coherence	Spectral
119	pdcoh_multitaper_mean_fs-1_fmin-0-25_fmax-0-5	pdcoh	Partial directed coherence	Spectral
120	pdcoh_multitaper_max_fs-1_fmin-0_fmax-0-5	pdcoh	Partial directed coherence	Spectral
121	pdcoh_multitaper_max_fs-1_fmin-0_fmax-0-25	pdcoh	Partial directed coherence	Spectral
122	pdcoh_multitaper_max_fs-1_fmin-0-25_fmax-0-5	pdcoh	Partial directed coherence	Spectral
123	gpdcoh_multitaper_mean_fs-1_fmin-0_fmax-0-5	gpdcoh	Generalized partial directed coherence	Spectral
124	gpdcoh_multitaper_mean_fs-1_fmin-0_fmax-0-25	gpdcoh	Generalized partial directed coherence	Spectral
125	gpdcoh_multitaper_mean_fs-1_fmin-0-25_fmax-0-5	gpdcoh	Generalized partial directed coherence	Spectral

126	gpdcoh_multitaper_max_fs-1_fmin-0_fmax-0-5	gpdcoh	Generalized partial directed coherence	Spectral
127	gpdcoh_multitaper_max_fs-1_fmin-0_fmax-0-25	gpdcoh	Generalized partial directed coherence	Spectral
128	gpdcoh_multitaper_max_fs-1_fmin-0-25_fmax-0-5	gpdcoh	Generalized partial directed coherence	Spectral
129	ddtf_multitaper_mean_fs-1_fmin-0_fmax-0-5	ddtf	Direct directed transfer function	Spectral
130	ddtf_multitaper_mean_fs-1_fmin-0_fmax-0-25	ddtf	Direct directed transfer function	Spectral
131	ddtf_multitaper_mean_fs-1_fmin-0-25_fmax-0-5	ddtf	Direct directed transfer function	Spectral
132	ddtf_multitaper_max_fs-1_fmin-0_fmax-0-5	ddtf	Direct directed transfer function	Spectral
133	ddtf_multitaper_max_fs-1_fmin-0_fmax-0-25	ddtf	Direct directed transfer function	Spectral
134	ddtf_multitaper_max_fs-1_fmin-0-25_fmax-0-5	ddtf	Direct directed transfer function	Spectral
135	sgc_nonparametric_mean_fs-1_fmin-0_fmax-0-5	sgc	Spectral Granger causality	Spectral
136	sgc_nonparametric_mean_fs-1_fmin-0_fmax-0-25	sgc	Spectral Granger causality	Spectral
137	sgc_nonparametric_mean_fs-1_fmin-0-25_fmax-0-5	sgc	Spectral Granger causality	Spectral
138	sgc_nonparametric_max_fs-1_fmin-0_fmax-0-5	sgc	Spectral Granger causality	Spectral
139	sgc_nonparametric_max_fs-1_fmin-0_fmax-0-25	sgc	Spectral Granger causality	Spectral
140	sgc_nonparametric_max_fs-1_fmin-0-25_fmax-0-5	sgc	Spectral Granger causality	Spectral
141	sgc_parametric_mean_fs-1_fmin-0_fmax-0-5_order-None	sgc	Spectral Granger causality	Spectral
142	sgc_parametric_mean_fs-1_fmin-0_fmax-0-25_order-None	sgc	Spectral Granger causality	Spectral
143	sgc_parametric_mean_fs-1_fmin-0-25_fmax-0-5_order-None	sgc	Spectral Granger causality	Spectral
144	sgc_parametric_mean_fs-1_fmin-1e-05_fmax-0-5_order-1	sgc	Spectral Granger causality	Spectral
145	sgc_parametric_mean_fs-1_fmin-0_fmax-0-25_order-1	sgc	Spectral Granger causality	Spectral
146	sgc_parametric_mean_fs-1_fmin-0-25_fmax-0-5_order-1	sgc	Spectral Granger causality	Spectral
147	sgc_parametric_mean_fs-1_fmin-1e-05_fmax-0-5_order-20	sgc	Spectral Granger causality	Spectral
148	sgc_parametric_mean_fs-1_fmin-0_fmax-0-25_order-20	sgc	Spectral Granger causality	Spectral
149	sgc_parametric_mean_fs-1_fmin-0-25_fmax-0-5_order-20	sgc	Spectral Granger causality	Spectral
150	sgc_parametric_max_fs-1_fmin-1e-05_fmax-0-5_order-None	sgc	Spectral Granger causality	Spectral
151	sgc_parametric_max_fs-1_fmin-0_fmax-0-25_order-None	sgc	Spectral Granger causality	Spectral
152	sgc_parametric_max_fs-1_fmin-0-25_fmax-0-5_order-None	sgc	Spectral Granger causality	Spectral
153	sgc_parametric_max_fs-1_fmin-1e-05_fmax-0-5_order-1	sgc	Spectral Granger causality	Spectral
154	sgc_parametric_max_fs-1_fmin-0_fmax-0-25_order-1	sgc	Spectral Granger causality	Spectral
155	sgc_parametric_max_fs-1_fmin-0-25_fmax-0-5_order-1	sgc	Spectral Granger causality	Spectral

156	sgc_parametric_max_fs-1_fmin-1e-05_fmax-0-5_order-20	sgc	Spectral Granger causality	Spectral
157	sgc_parametric_max_fs-1_fmin-0_fmax-0-25_order-20	sgc	Spectral Granger causality	Spectral
158	sgc_parametric_max_fs-1_fmin-0-25_fmax-0-5_order-20	sgc	Spectral Granger causality	Spectral
159	lmfit_Ridge	lmfit	Linear model fit	Miscellaneous
160	lmfit_SGDRegressor	lmfit	Linear model fit	Miscellaneous
161	lmfit_ElasticNet	lmfit	Linear model fit	Miscellaneous
162	lmfit_BayesianRidge	lmfit	Linear model fit	Miscellaneous
163	coint_johansen_max_eig_stat_order-0_ardiff-10	coint	Cointegration	Miscellaneous
164	coint_johansen_trace_stat_order-0_ardiff-10	coint	Cointegration	Miscellaneous
165	coint_johansen_max_eig_stat_order-0_ardiff-1	coint	Cointegration	Miscellaneous
166	coint_johansen_trace_stat_order-0_ardiff-1	coint	Cointegration	Miscellaneous
167	coint_johansen_max_eig_stat_order-1_ardiff-10	coint	Cointegration	Miscellaneous
168	coint_johansen_trace_stat_order-1_ardiff-10	coint	Cointegration	Miscellaneous
169	coint_johansen_max_eig_stat_order-1_ardiff-1	coint	Cointegration	Miscellaneous
170	coint_johansen_trace_stat_order-1_ardiff-1	coint	Cointegration	Miscellaneous
171	coint_aeg_tstat_trend-c_autolag-aic_maxlag-10	coint	Cointegration	Miscellaneous
172	coint_aeg_tstat_trend-ct_autolag-aic_maxlag-10	coint	Cointegration	Miscellaneous
173	coint_aeg_tstat_trend-ct_autolag-bic_maxlag-10	coint	Cointegration	Miscellaneous
174	pec	pec	Power envelope correlation	Miscellaneous
175	pec_orth	pec	Power envelope correlation	Miscellaneous
176	pec_log	pec	Power envelope correlation	Miscellaneous
177	pec_orth_log	pec	Power envelope correlation	Miscellaneous
178	pec_orth_abs	pec	Power envelope correlation	Miscellaneous
179	pec_orth_log_abs	pec	Power envelope correlation	Miscellaneous

Supplementary Table 3: | Directed *pyspi* statistics (excluding those only with opposite sign) Pairwise statistics with different upper and lower triangular values excluding those only differ by a sign.

Index	Statistic	Measure	Description	Category
49	dcorrx_maxlag-1	dcorrx	Cross distance correlation	Distance similarity
71	cds	cds	Conditional distribution similarity fit	Causal inference
72	reci	reci	Regression error-based causal inference	Causal inference
78	ce_kozachenko	ce	Conditional entropy	Information theory
79	ce_kernel_W-0.5	ce	Conditional entropy	Information theory
80	cce_gaussian	cce	Causally conditioned entropy	Information theory
81	cce_kernel_W-0.5	cce	Causally conditioned entropy	Information theory
82	xme_gaussian_k1	xme	Cross-map entropy	Information theory
83	xme_kozachenko_k1	xme	Cross-map entropy	Information theory
84	xme_kernel_W-0.5_k1	xme	Cross-map entropy	Information theory
85	xme_gaussian_k10	xme	Cross-map entropy	Information theory
86	xme_kernel_W-0.5_k10	xme	Cross-map entropy	Information theory
87	di_gaussian	di	Directed information	Information theory
88	di_kernel_W-0.5	di	Directed information	Information theory
99	te_kraskov_NN-4_DCE_k-2_kt-1_l-1_lt-1	te	Transfer entropy	Information theory
100	te_kraskov_NN-4_DCE_k-1_kt-1_l-1_lt-1	te	Transfer entropy	Information theory
101	te_kraskov_NN-4_k-1_kt-1_l-1_lt-1	te	Transfer entropy	Information theory
102	te_kernel_W-0.25_k-1	te	Transfer entropy	Information theory
103	gc_gaussian_k-max-10_tau-max-2	te	Transfer entropy	Information theory
104	gc_gaussian_k-1_kt-1_l-1_lt-1	te	Transfer entropy	Information theory
105	te_symbolic_k-1_kt-1_l-1_lt-1	te	Transfer entropy	Information theory
106	te_symbolic_k-10_kt-1_l-1_lt-1	te	Transfer entropy	Information theory
111	phase_multitaper_mean_fs-1_fmin-0_fmax-0-5	phase	Coherence phase	Spectral
112	phase_multitaper_mean_fs-1_fmin-0_fmax-0-25	phase	Coherence phase	Spectral
114	phase_multitaper_max_fs-1_fmin-0_fmax-0-5	phase	Coherence phase	Spectral
115	phase_multitaper_max_fs-1_fmin-0_fmax-0-25	phase	Coherence phase	Spectral
116	phase_multitaper_max_fs-1_fmin-0-25_fmax-0-5	phase	Coherence phase	Spectral
141	pli_multitaper_max_fs-1_fmin-0_fmax-0-25	pli	Phase lag index	Spectral
142	pli_multitaper_max_fs-1_fmin-0-25_fmax-0-5	pli	Phase lag index	Spectral
146	wpli_multitaper_max_fs-1_fmin-0_fmax-0-25	wpli	Weighted phase lag index	Spectral
159	dtf_multitaper_mean_fs-1_fmin-0_fmax-0-5	dtf	Directed transfer function	Spectral
160	dtf_multitaper_mean_fs-1_fmin-0_fmax-0-25	dtf	Directed transfer function	Spectral
161	dtf_multitaper_mean_fs-1_fmin-0-25_fmax-0-5	dtf	Directed transfer function	Spectral
162	dtf_multitaper_max_fs-1_fmin-0_fmax-0-5	dtf	Directed transfer function	Spectral
163	dtf_multitaper_max_fs-1_fmin-0_fmax-0-25	dtf	Directed transfer function	Spectral
164	dtf_multitaper_max_fs-1_fmin-0-25_fmax-0-5	dtf	Directed transfer function	Spectral
165	dcoh_multitaper_mean_fs-1_fmin-0_fmax-0-5	dcoh	Directed coherence	Spectral
166	dcoh_multitaper_mean_fs-1_fmin-0_fmax-0-25	dcoh	Directed coherence	Spectral

167	dcoh_multitaper_mean_fs-1_fmin-0-25_fmax-0-5	dcoh	Directed coherence	Spectral
168	dcoh_multitaper_max_fs-1_fmin-0_fmax-0-5	dcoh	Directed coherence	Spectral
169	dcoh_multitaper_max_fs-1_fmin-0_fmax-0-25	dcoh	Directed coherence	Spectral
170	dcoh_multitaper_max_fs-1_fmin-0-25_fmax-0-5	dcoh	Directed coherence	Spectral
171	pdcoh_multitaper_mean_fs-1_fmin-0_fmax-0-5	pdcoh	Partial directed coherence	Spectral
172	pdcoh_multitaper_mean_fs-1_fmin-0_fmax-0-25	pdcoh	Partial directed coherence	Spectral
173	pdcoh_multitaper_mean_fs-1_fmin-0-25_fmax-0-5	pdcoh	Partial directed coherence	Spectral
174	pdcoh_multitaper_max_fs-1_fmin-0_fmax-0-5	pdcoh	Partial directed coherence	Spectral
175	pdcoh_multitaper_max_fs-1_fmin-0_fmax-0-25	pdcoh	Partial directed coherence	Spectral
176	pdcoh_multitaper_max_fs-1_fmin-0-25_fmax-0-5	pdcoh	Partial directed coherence	Spectral
177	gpdcoh_multitaper_mean_fs-1_fmin-0_fmax-0-5	gpdcoh	Generalized partial directed coherence	Spectral
178	gpdcoh_multitaper_mean_fs-1_fmin-0_fmax-0-25	gpdcoh	Generalized partial directed coherence	Spectral
179	gpdcoh_multitaper_mean_fs-1_fmin-0-25_fmax-0-5	gpdcoh	Generalized partial directed coherence	Spectral
180	gpdcoh_multitaper_max_fs-1_fmin-0_fmax-0-5	gpdcoh	Generalized partial directed coherence	Spectral
181	gpdcoh_multitaper_max_fs-1_fmin-0_fmax-0-25	gpdcoh	Generalized partial directed coherence	Spectral
182	gpdcoh_multitaper_max_fs-1_fmin-0-25_fmax-0-5	gpdcoh	Generalized partial directed coherence	Spectral
183	ddtf_multitaper_mean_fs-1_fmin-0_fmax-0-5	ddtf	Direct directed transfer function	Spectral
184	ddtf_multitaper_mean_fs-1_fmin-0_fmax-0-25	ddtf	Direct directed transfer function	Spectral
185	ddtf_multitaper_mean_fs-1_fmin-0-25_fmax-0-5	ddtf	Direct directed transfer function	Spectral
186	ddtf_multitaper_max_fs-1_fmin-0_fmax-0-5	ddtf	Direct directed transfer function	Spectral
187	ddtf_multitaper_max_fs-1_fmin-0_fmax-0-25	ddtf	Direct directed transfer function	Spectral
188	ddtf_multitaper_max_fs-1_fmin-0-25_fmax-0-5	ddtf	Direct directed transfer function	Spectral
189	sgc_nonparametric_mean_fs-1_fmin-0_fmax-0-5	sgc	Spectral Granger causality	Spectral
190	sgc_nonparametric_mean_fs-1_fmin-0_fmax-0-25	sgc	Spectral Granger causality	Spectral
191	sgc_nonparametric_mean_fs-1_fmin-0-25_fmax-0-5	sgc	Spectral Granger causality	Spectral
192	sgc_nonparametric_max_fs-1_fmin-0_fmax-0-5	sgc	Spectral Granger causality	Spectral
193	sgc_nonparametric_max_fs-1_fmin-0_fmax-0-25	sgc	Spectral Granger causality	Spectral
194	sgc_nonparametric_max_fs-1_fmin-0-25_fmax-0-5	sgc	Spectral Granger causality	Spectral
195	sgc_parametric_mean_fs-1_fmin-0_fmax-0-5_order-None	sgc	Spectral Granger causality	Spectral
196	sgc_parametric_mean_fs-1_fmin-0_fmax-0-25_order-None	sgc	Spectral Granger causality	Spectral
197	sgc_parametric_mean_fs-1_fmin-0-25_fmax-0-5_order-None	sgc	Spectral Granger causality	Spectral

198	sgc_parametric_mean_fs-1_fmin-1e-05_fmax-0-5_order-1	sgc	Spectral Granger causality	Spectral
199	sgc_parametric_mean_fs-1_fmin-0_fmax-0-25_order-1	sgc	Spectral Granger causality	Spectral
200	sgc_parametric_mean_fs-1_fmin-0-25_fmax-0-5_order-1	sgc	Spectral Granger causality	Spectral
201	sgc_parametric_mean_fs-1_fmin-1e-05_fmax-0-5_order-20	sgc	Spectral Granger causality	Spectral
202	sgc_parametric_mean_fs-1_fmin-0_fmax-0-25_order-20	sgc	Spectral Granger causality	Spectral
203	sgc_parametric_mean_fs-1_fmin-0-25_fmax-0-5_order-20	sgc	Spectral Granger causality	Spectral
204	sgc_parametric_max_fs-1_fmin-1e-05_fmax-0-5_order-None	sgc	Spectral Granger causality	Spectral
205	sgc_parametric_max_fs-1_fmin-0_fmax-0-25_order-None	sgc	Spectral Granger causality	Spectral
206	sgc_parametric_max_fs-1_fmin-0-25_fmax-0-5_order-None	sgc	Spectral Granger causality	Spectral
207	sgc_parametric_max_fs-1_fmin-1e-05_fmax-0-5_order-1	sgc	Spectral Granger causality	Spectral
208	sgc_parametric_max_fs-1_fmin-0_fmax-0-25_order-1	sgc	Spectral Granger causality	Spectral
209	sgc_parametric_max_fs-1_fmin-0-25_fmax-0-5_order-1	sgc	Spectral Granger causality	Spectral
210	sgc_parametric_max_fs-1_fmin-1e-05_fmax-0-5_order-20	sgc	Spectral Granger causality	Spectral
211	sgc_parametric_max_fs-1_fmin-0_fmax-0-25_order-20	sgc	Spectral Granger causality	Spectral
212	sgc_parametric_max_fs-1_fmin-0-25_fmax-0-5_order-20	sgc	Spectral Granger causality	Spectral
220	lmfit_SGDRegressor	lmfit	Linear model fit	Miscellaneous

Supplementary Table 4: | **Directed *pyspi* statistics with opposite sign** Pairwise statistics with different upper and lower triangular values that only differ by a sign.

Index	Statistic	Measure	Description	Category
73	igci	igci	Information-geometric causal inference	Causal inference
113	phase_multitaper_mean_fs-1_fmin-0-25_fmax-0-5	phase	Coherence phase	Spectral
129	psi_multitaper_mean_fs-1_fmin-0_fmax-0-5	psi	Phase slope index	Spectral
130	psi_multitaper_mean_fs-1_fmin-0_fmax-0-25	psi	Phase slope index	Spectral
131	psi_multitaper_mean_fs-1_fmin-0-25_fmax-0-5	psi	Phase slope index	Spectral
138	pli_multitaper_mean_fs-1_fmin-0_fmax-0-5	pli	Phase lag index	Spectral
139	pli_multitaper_mean_fs-1_fmin-0_fmax-0-25	pli	Phase lag index	Spectral
140	pli_multitaper_mean_fs-1_fmin-0-25_fmax-0-5	pli	Phase lag index	Spectral
143	wpli_multitaper_mean_fs-1_fmin-0_fmax-0-5	wpli	Weighted phase lag index	Spectral
144	wpli_multitaper_mean_fs-1_fmin-0_fmax-0-25	wpli	Weighted phase lag index	Spectral
145	wpli_multitaper_mean_fs-1_fmin-0-25_fmax-0-5	wpli	Weighted phase lag index	Spectral

Supplementary Table 5: | **Individual and composite ranks of 239 *pyspi* statistics** Six potentially desirable criteria evaluated through the project were used to rank the 239 pairwise statistics. The criteria are: (1) negative weight–distance relationship (W-D), (2) positive structure–function coupling (SFC), (3) close correspondence with biological similarity networks (BSN), (4) high individual–participant identifiability (Fp), (5) high brain–behavior prediction (BB), and (6) low susceptibility to participant motion (Mt). Note that (3) was derived as a composite ranking averaging over the 5 neurophysiological networks in Fig. 3, (5) was derived as a composite ranking averaging over the 5 cognitive-behavior predictors in Fig. 4b, and (6) was derived as a composite ranking averaging over the two motion metrics in Fig. 11. The overall composite ranking (All) was derived by averaging the six individual rankings, and was used to reorder the final table. Tied elements were assigned ranks using the default strategy in *scipy.stats.rankdata*. `NaN` elements were ignored during the ranking.

Index	Statistic	W-D	SFC	BSN	Fp	BB	Mt	All
21	prec_ShrunkCovariance	3	1	83	1	1	61	1
17	prec_GraphicalLassoCV	17	39	41.5	2	95	NaN	2
20	prec_OAS	26	4	116	4	64	46	3
117	cohmag_multitaper_mean_fs-1_fmin-0_fmax-0-5	18	17	2	87	4	140	4
89	si_gaussian_k-1	2	15	1	25	31	201.5	5
15	prec_EmpiricalCovariance	32	5	122	5	68.5	45	6
133	plv_multitaper_mean_fs-1_fmin-0_fmax-0-25	14	28	10.5	78	6	148	7
19	prec_MinCovDet	28	6	118	7	89.5	40.5	8
18	prec_LedoitWolf	25	3	110	3	101	49	9
118	cohmag_multitaper_mean_fs-1_fmin-0_fmax-0-25	13	26	12	61	27	157	10
16	prec_EllipticEnvelope	27	7	117	6	100	40.5	11
132	plv_multitaper_mean_fs-1_fmin-0_fmax-0-5	19	16	3	98	33	131	12
154	ppc_multitaper_mean_fs-1_fmin-0_fmax-0-25	12	27	14	66	38	154	13
177	gpdcoh_multitaper_mean_fs-1_fmin-0_fmax-0-5	41	35	22	105	79	76	14
153	ppc_multitaper_mean_fs-1_fmin-0_fmax-0-5	16	18	4	93	89.5	138	15
123	icoh_multitaper_mean_fs-1_fmin-0_fmax-0-5	7	12	16	142	131	57.5	16
77	ce_gaussian	46	44	49	23	19	195.5	17
94	mi_kernel_W-0.25	70	49	46.5	30	37	160.5	18
74	je_gaussian	45	45	46.5	24	46	191	19
124	icoh_multitaper_mean_fs-1_fmin-0_fmax-0-25	38	30	43	97	133	63	20.5
42	pdist_chebyshev	34	38	28	96	58	150	20.5
222	lmfit_BayesianRidge	51	56	62	31	14	191	22
220	lmfit_SGDRegressor	52	52	61	32	26	183	23
92	mi_kraskov_NN-4	72	48	39	63	42	151	24
91	mi_gaussian	44	43	48	22	63	195.5	25
93	mi_kraskov_NN-4_DCE	47	46	51	57	60	155.5	26
85	xme_gaussian_k10	36	70	77	45	10	181	27
219	lmfit_Ridge	53	55	65	37	18	195.5	28
79	ce_kernel_W-0.5	97	66	58.5	39	20	145	29
14	cov-sq_ShrunkCovariance	54.5	54	63.5	35	24	195.5	30
48	hsic_biased	61	60	54	49	39	166.5	31
221	lmfit_ElasticNet	33	42	35.5	64	142	116	32
114	phase_multitaper_max_fs-1_fmin-0_fmax-0-5	30	29	7	204	121	42.5	33
56	lcss	37	78	24.5	62	72	162.5	34
31	kendalltau-sq	48	50	70	29	50	195.5	35
70	anm	43	47	44.5	77	76	155.5	36
24	prec-sq_GraphicalLassoCV	35	162	19	15	147	NaN	37

45	dcorr	64	61	71.5	44	35	168	38
126	icoh_multitaper_max_fs-1_fmin-0_fmax-0-5	15	9	18	203	187	17	39
8	cov-sq_EmpiricalCovariance	54.5	53	63.5	33	53	195.5	40
76	je_kernel_W-0.5	99	71	97.5	48	15	130	41
34	xcorr-sq_max_sig-True	66	63	53	53	65.5	160.5	42
46	dcorr_biased	65	62	73	43	54	169	43
97	tlmi_kraskov_NN-4_DCE	63	82	88	84	3	147	44
47	hsic	60	59	52	51	80	166.5	45
13	cov-sq_OAS	50	58	66	35	59	201.5	46
125	icoh_multitaper_mean_fs-1_fmin-0-25_fmax-0-5	1	2	5	220	230	13.5	47
82	xme_gaussian_k1	68	81	90.5	41	17	181	48
96	tlmi_kraskov_NN-4	92	83	74	86	5	139	49
29	spearmanr-sq	57	64	78	40	52	189	50
84	xme_kernel_W-0.5_k1	103	93	89	60	2	136.5	51
95	tlmi_gaussian	59	80	92	42	40	177	52
178	gpdcoh_multitaper_mean_fs-1_fmin-0_fmax-0-25	62	68	56	81	141	85	53
115	phase_multitaper_max_fs-1_fmin-0_fmax-0-25	22	21	8	181	217	52	54
180	gpdcoh_multitaper_max_fs-1_fmin-0_fmax-0-5	42	36	23	161	176.5	64.5	55
11	cov-sq_LedoitWolf	49	57	69	35	93	204	56.5
52	dtw_constraint-sakoe-chiba	23	69	76	46	65.5	227.5	56.5
120	cohmag_multitaper_max_fs-1_fmin-0_fmax-0-5	4	32	13	176	199.5	83	58
127	icoh_multitaper_max_fs-1_fmin-0_fmax-0-25	31	22	24.5	183	223	34.5	59
156	ppc_multitaper_max_fs-1_fmin-0_fmax-0-5	5	33	9	200	233	38.5	60
135	plv_multitaper_max_fs-1_fmin-0_fmax-0-5	6	34	10.5	199	231	38.5	61
98	tlmi_kernel_W-0.25	91	84	81	56	56.5	152.5	62
49	dcorrx_maxlag-1	93	79	86	52	51	162.5	63
181	gpdcoh_multitaper_max_fs-1_fmin-0_fmax-0-25	56	51	38	154	160	66.5	64
128	icoh_multitaper_max_fs-1_fmin-0-25_fmax-0-5	24	13	32.5	227	226	7	65
234	pec	58	73	44.5	100	114	143	66
28	prec-sq_ShrunkCovariance	123	133	17	111	110.5	44	67
179	gpdcoh_multitaper_mean_fs-1_fmin-0-25_fmax-0-5	67	25	6	211	205	25	68
58	lcss_constraint-sakoe-chiba	20	67	79	38	112	227.5	69
121	cohmag_multitaper_max_fs-1_fmin-0_fmax-0-25	9	37	26	171	204	100	70.5
157	ppc_multitaper_max_fs-1_fmin-0_fmax-0-25	10	40	20	194	235.5	47.5	70.5
33	xcorr_max_sig-True	89	88	87	76	44.5	164.5	72.5
136	plv_multitaper_max_fs-1_fmin-0_fmax-0-25	11	41	21	193	235.5	47.5	72.5
66	bary-sq_dtw_mean	29	85	80	75	55	226	74.5
75	je_kozachenko	100	72	93	116	56.5	112.5	74.5
119	cohmag_multitaper_mean_fs-1_fmin-0-25_fmax-0-5	117	10	29	208	127	60	76
90	si_kernel_W-0.5_k-1	21	77	145	106	28	175	77
9	cov-sq_EllipticEnvelope	82	74	84	79	25	210	78
78	ce_kozachenko	98	65	50	163	73.5	105	79
57	lcss_constraint-itakura	8	31	15	224	207	71	80
158	ppc_multitaper_max_fs-1_fmin-0-25_fmax-0-5	115	19	35.5	218	166	15.5	81
68	bary-sq_sgddtw_mean	75	110	101	68	7.5	211.5	82
137	plv_multitaper_max_fs-1_fmin-0-25_fmax-0-5	112	20	30	219	185	15.5	83

43	pdist_canberra	73	87	105	58	48.5	211.5	84
155	ppc_multitaper_mean_fs-1_fmin-0-25_fmax-0-5	116	11	37	209	156	57.5	85.5
116	phase_multitaper_max_fs-1_fmin-0-25_fmax-0-5	101	24	27	212	201	21.5	85.5
134	plv_multitaper_mean_fs-1_fmin-0-25_fmax-0-5	111	8	32.5	210	173.5	56	87
64	bary-sq_euclidean_mean	80	103	109	69	16	217.5	88
12	cov-sq_MinCovDet	85	75	85	82	68.5	205	89
39	pdist_euclidean	71	86	100	54	75	217.5	90
182	gpdcoh_multitaper_max_fs-1_fmin-0-25_fmax-0-5	105	23	34	213	202	27	91.5
7	cov_ShrunkCovariance	78.5	104	111.5	71.5	21	217.5	91.5
109	phi_Geo_t-1_norm-0	39	100	150	16	98	207	93
40	pdist_cityblock	84	95	120	55	47	213.5	94
209	sgc_parametric_max_fs-1_fmin-0-25_fmax-0-5_order-1	90	118	142	9	119.5	142	95
4	cov_LedoitWolf	76	108	113	71.5	29.5	223	96.5
110	phi_Geo_t-1_norm-1	40	99	151	17	105	209	96.5
51	dtw_constraint-itakura	185	159	60	122	7.5	92.5	98
203	sgc_parametric_mean_fs-1_fmin-0-25_fmax-0-5_order-20	124	96	57	175	145	33	100
83	xme_kozachenko_k1	104	92	82	174	91	87	100
6	cov_OAS	77	109	114.5	71.5	36	222	100
236	pec_log	69	76	67.5	107	165	146	102
195	sgc_parametric_mean_fs-1_fmin-0_fmax-0-5_order-None	122	122	137	28	107	115	103
122	cohmag_multitaper_max_fs-1_fmin-0-25_fmax-0-5	118	14	31	214	228.5	27	104
41	pdist_cosine	81	106	106	74	48.5	217.5	105
10	cov-sq_GraphicalLassoCV	129	90	71.5	85	153	108	106
200	sgc_parametric_mean_fs-1_fmin-0-25_fmax-0-5_order-1	94	119	149	8	124	149	107
1	**cov_EmpiricalCovariance**	78.5	105	111.5	71.5	62	217.5	108
30	spearmanr	86	112	126	67	42	213.5	109
87	di_gaussian	157	127	95	115	29.5	124.5	110
212	sgc_parametric_max_fs-1_fmin-0-25_fmax-0-5_order-20	121	97	55	178	175	24	111
44	pdist_braycurtis	87	114	127	65	42	217.5	112
32	kendalltau	83	98	119	59	73.5	221	113
201	sgc_parametric_mean_fs-1_fmin-1e-05_fmax-0-5_order-20	135	124	130	50	130	88	114
162	dtf_multitaper_max_fs-1_fmin-0_fmax-0-5	109.5	141	103.5	132.5	71	105	115
198	sgc_parametric_mean_fs-1_fmin-1e-05_fmax-0-5_order-1	119	125	134	13	147	128.5	116
108	phi_star_t-1_norm-1	152	102	97.5	102	126	89.5	117
199	sgc_parametric_mean_fs-1_fmin-0_fmax-0-25_order-1	130	132	135	14	134.5	124.5	118
206	sgc_parametric_max_fs-1_fmin-0-25_fmax-0-5_order-None	106	91	124	150	122.5	77	119
175	pdcoh_multitaper_max_fs-1_fmin-0_fmax-0-25	113.5	137	107.5	127.5	77	109.5	120
2	cov_EllipticEnvelope	96	115	128	88	22	225	121
107	phi_star_t-1_norm-0	151	101	96	101	136.5	89.5	122
163	dtf_multitaper_max_fs-1_fmin-0_fmax-0-25	113.5	136	107.5	127.5	83	109.5	123
50	dtw	206	166	75	124	9	101.5	124
5	cov_MinCovDet	95	116	129	89	34	224	125
196	sgc_parametric_mean_fs-1_fmin-0_fmax-0-25_order-None	133	131	162	26	118	118	126
3	cov_GraphicalLassoCV	132	120	125	103	116.5	94	127

65	bary-sq_euclidean_max	88	94	102	195	116.5	97	128.5
27	prec-sq_OAS	177	183	41.5	125	113	53	128.5
38	xcorr-sq_mean_sig-False	153	148	94	151	32	120	130
36	xcorr-sq_mean_sig-True	142	126	99	117	96	121	131
202	sgc_parametric_mean_fs-1_fmin-0_fmax-0-25_order-20	143	130	148	47	129	105	132
99	te_kraskov_NN-4_DCE_k-2_kt-1_l-1_lt-1	102	142	179	134	87.5	62	133
106	te_symbolic_k-10_kt-1_l-1_lt-1	108	146	168	10	125	158	134
197	sgc_parametric_mean_fs-1_fmin-0-25_fmax-0-5_order-None	107	89	90.5	153	212	64.5	135
104	gc_gaussian_k-1_kt-1_l-1_lt-1	127.5	154.5	174.5	11.5	78	175	136
174	pdcoh_multitaper_max_fs-1_fmin-0_fmax-0-5	109.5	140	103.5	132.5	139	105	137
103	gc_gaussian_k-max-10_tau-max-2	131	150	165	27	136.5	133	138
105	te_symbolic_k-1_kt-1_l-1_lt-1	127.5	154.5	174.5	11.5	110.5	175	139
59	bary_euclidean_max	74	111	131	201	162.5	84	140
25	prec-sq_LedoitWolf	176	181	40	123	191.5	55	141
189	sgc_nonparametric_mean_fs-1_fmin-0_fmax-0-5	136	123	133	99	172	NaN	142
69	bary-sq_sgddtw_max	126	121	136	198	140	70	143
55	softdtw_constraint-sakoe-chiba	223	169	140	140	11	112.5	144
72	reci	137	113	132	186	103	128.5	145
211	sgc_parametric_max_fs-1_fmin-0_fmax-0-25_order-20	164	156	181	80	134.5	86	146
22	prec-sq_EmpiricalCovariance	189	192	58.5	135	184	54	147
204	sgc_parametric_max_fs-1_fmin-1e-05_fmax-0-5_order-None	178	164	188	19	162.5	101.5	148
26	prec-sq_MinCovDet	209	193	121	146	102	51	149
210	sgc_parametric_max_fs-1_fmin-1e-05_fmax-0-5_order-20	165	153	173	83	170.5	81.5	150
205	sgc_parametric_max_fs-1_fmin-0_fmax-0-25_order-None	180	165	189	18	170.5	105	151
53	softdtw	223	169	140	140	44.5	112.5	152
54	softdtw_constraint-itakura	223	169	140	140	61	112.5	153
190	sgc_nonparametric_mean_fs-1_fmin-0_fmax-0-25	138	129	154	92	207	NaN	154
191	sgc_nonparametric_mean_fs-1_fmin-0-25_fmax-0-5	134	107	67.5	206	207	NaN	155
63	bary_sgddtw_max	125	128	138	205	183	78.5	156
23	prec-sq_EllipticEnvelope	208	194	123	145	143	50	157
172	pdcoh_multitaper_mean_fs-1_fmin-0_fmax-0-25	160.5	179.5	159.5	110	82	187	158
67	bary-sq_dtw_max	146	135	157	202	169	74	159
207	sgc_parametric_max_fs-1_fmin-1e-05_fmax-0-5_order-1	187	185	177	21	193.5	122.5	160
35	xcorr_mean_sig-True	147	149	143	190	179	78.5	161
225	coint_johansen_max_eig_stat_order-0_ardiff-1	190.5	190	230.5	90.5	13	172.5	162
100	te_kraskov_NN-4_DCE_k-1_kt-1_l-1_lt-1	120	152	183	147	213	73	163
164	dtf_multitaper_max_fs-1_fmin-0-25_fmax-0-5	158.5	174.5	155.5	158.5	147	95.5	164
208	sgc_parametric_max_fs-1_fmin-0_fmax-0-25_order-1	188	184	176	20	203	122.5	165
226	coint_johansen_trace_stat_order-0_ardiff-1	182.5	172	222.5	94.5	93	133	166
230	coint_johansen_trace_stat_order-1_ardiff-1	182.5	171	222.5	94.5	97	133	167
176	pdcoh_multitaper_max_fs-1_fmin-0-25_fmax-0-5	158.5	174.5	155.5	158.5	159	95.5	168

160	dtf_multitaper_mean_fs-1_fmin-0_fmax-0-25	160.5	179.5	159.5	109	109	187	169
88	di_kernel_W-0.5	149	151	200	104	157	144	170
187	ddtf_multitaper_max_fs-1_fmin-0_fmax-0-25	166	160	152.5	120	189	118	171
81	cce_kernel_W-0.5	154	163	144	112	149	187	172
112	phase_multitaper_mean_fs-1_fmin-0_fmax-0-25	205	214	234	166	23	72	173
235	pec_orth	210	177	204.5	191	99	34.5	174
186	ddtf_multitaper_max_fs-1_fmin-0_fmax-0-5	167	161	152.5	121	198	118	175
192	sgc_nonparametric_max_fs-1_fmin-0_fmax-0-5	155	134	164	170	152	NaN	176
101	te_kraskov_NN-4_k-1_kt-1_l-1_lt-1	150	158	184	144	209	75	177
61	bary_dtw_max	139	147	158	207	182	91	178
143	wpli_multitaper_mean_fs-1_fmin-0_fmax-0-5	195	221	212	157	84	69	179
147	dspli_multitaper_mean_fs-1_fmin-0_fmax-0-5	221	201	202	169	128	19	180
232	coint_aeg_tstat_trend-ct_autolag-aic_maxlag-10	202.5	207.5	214.5	179	12	126.5	181
193	sgc_nonparametric_max_fs-1_fmin-0_fmax-0-25	156	145	168	164	164	NaN	182.5
183	ddtf_multitaper_mean_fs-1_fmin-0_fmax-0-5	163	195	179	113	93	203	182.5
80	cce_gaussian	227	186	170	138	86	141	184.5
139	pli_multitaper_mean_fs-1_fmin-0_fmax-0-25	192	222	221	152	81	80	184.5
138	pli_multitaper_mean_fs-1_fmin-0_fmax-0-5	194	223	217	162	85	68	186
184	ddtf_multitaper_mean_fs-1_fmin-0_fmax-0-25	162	176	168	108	154.5	181	187
194	sgc_nonparametric_max_fs-1_fmin-0-25_fmax-0-5	148	117	114.5	223	199.5	NaN	188
166	dcoh_multitaper_mean_fs-1_fmin-0_fmax-0-25	181	167	163	114	167	164.5	189
229	coint_johansen_max_eig_stat_order-1_ardiff-1	190.5	189	230.5	90.5	87.5	172.5	190
86	xme_kernel_W-0.5_k10	186	157	192	155	138	135	191
144	wpli_multitaper_mean_fs-1_fmin-0_fmax-0-25	193	220	216	148	105	81.5	192.5
150	dswpli_multitaper_mean_fs-1_fmin-0_fmax-0-5	225	203	196.5	167	150.5	21.5	192.5
148	dspli_multitaper_mean_fs-1_fmin-0_fmax-0-25	212	187	199	165	181	30	194
159	dtf_multitaper_mean_fs-1_fmin-0_fmax-0-5	168.5	197.5	171.5	118.5	119.5	207	195
111	phase_multitaper_mean_fs-1_fmin-0_fmax-0-5	201	213	233	173	105	59	196
237	pec_orth_log	214	182	203	196	168	21.5	197
171	pdcoh_multitaper_mean_fs-1_fmin-0_fmax-0-5	168.5	197.5	171.5	118.5	122.5	207	198
151	dswpli_multitaper_mean_fs-1_fmin-0_fmax-0-25	213	191	196.5	160	195.5	32	199
169	dcoh_multitaper_max_fs-1_fmin-0_fmax-0-25	199	229	146	136	191.5	98.5	200.5
233	coint_aeg_tstat_trend-ct_autolag-bic_maxlag-10	216	225	227.5	172	67	92.5	200.5
231	coint_aeg_tstat_trend-c_autolag-aic_maxlag-10	202.5	207.5	214.5	180	70	126.5	202
224	coint_johansen_trace_stat_order-0_ardiff-10	144.5	138	225.5	187.5	108	199.5	203
62	bary_sgddtw_mean	215	205	161	182	216	31	204

228	coint_johansen_trace_stat_order-1_ardiff-10	144.5	139	225.5	187.5	115	199.5	205
185	ddtf_multitaper_mean_fs-1_fmin-0-25_fmax-0-5	172	209	194	126	132	178.5	206
129	psi_multitaper_mean_fs-1_fmin-0_fmax-0-5	173	216	209.5	192	186	37	207
168	dcoh_multitaper_max_fs-1_fmin-0_fmax-0-5	200	230	147	143	197	98.5	208
223	coint_johansen_max_eig_stat_order-0_ardiff-10	140.5	144	218.5	184.5	158	170.5	209
102	te_kernel_W-0.25_k-1	230	196	182	129	144	136.5	210
165	dcoh_multitaper_mean_fs-1_fmin-0_fmax-0-5	174	188	166	137	176.5	191	211.5
188	ddtf_multitaper_max_fs-1_fmin-0-25_fmax-0-5	170	206	198	149	150.5	159	211.5
227	coint_johansen_max_eig_stat_order-1_ardiff-10	140.5	143	218.5	184.5	180	170.5	213
130	psi_multitaper_mean_fs-1_fmin-0_fmax-0-25	175	204	235	177	211	42.5	214
149	dspli_multitaper_mean_fs-1_fmin-0-25_fmax-0-5	204	178	227.5	238	193.5	4	215
71	cds	207	199	191	222	195.5	36	216
161	dtf_multitaper_mean_fs-1_fmin-0-25_fmax-0-5	197.5	210.5	185.5	130	154.5	184.5	217
73	igci	236	200	195	225	190	21.5	218
152	dswpli_multitaper_mean_fs-1_fmin-0-25_fmax-0-5	196	173	229	235	232	4	219
238	pec_orth_abs	234	212	208	189	221	13.5	220
37	xcorr_mean_sig-False	171	202	179	236	227	66.5	221
239	pec_orth_log_abs	237	219	206	197	214	11	222
170	dcoh_multitaper_max_fs-1_fmin-0-25_fmax-0-5	179	239	187	168	161	152.5	223
173	pdcoh_multitaper_mean_fs-1_fmin-0-25_fmax-0-5	197.5	210.5	185.5	131	178	184.5	224
60	bary_dtw_mean	238	217	193	215	218	12	225
167	dcoh_multitaper_mean_fs-1_fmin-0-25_fmax-0-5	184	218	190	156	173.5	178.5	226
218	psi_wavelet_max_fs-1_fmin-0-25_fmax-0-5_max	211	215	201	234	234	7	227
213	psi_wavelet_mean_fs-1_fmin-0_fmax-0-5_mean	233	236	220	232	188	1	228
113	phase_multitaper_mean_fs-1_fmin-0-25_fmax-0-5	217	226	209.5	221	222	18	229
145	wpli_multitaper_mean_fs-1_fmin-0-25_fmax-0-5	218	227	207	216	219	29	230
216	psi_wavelet_max_fs-1_fmin-0_fmax-0-5_max	226	224	204.5	233	225	4	231
140	pli_multitaper_mean_fs-1_fmin-0-25_fmax-0-5	220	228	211	217	228.5	27	232
131	psi_multitaper_mean_fs-1_fmin-0-25_fmax-0-5	219	231	238	226	215	9	233
217	psi_wavelet_max_fs-1_fmin-0_fmax-0-25_max	231	234	232	230	210	7	234
215	psi_wavelet_mean_fs-1_fmin-0-25_fmax-0-5_mean	235	237	213	237	224	2	235
214	psi_wavelet_mean_fs-1_fmin-0_fmax-0-25_mean	232	235	224	231	220	10	236
146	wpli_multitaper_max_fs-1_fmin-0_fmax-0-25	228	232	236	229	NaN	NaN	237
141	pli_multitaper_max_fs-1_fmin-0_fmax-0-25	229	233	237	228	NaN	NaN	238
142	pli_multitaper_max_fs-1_fmin-0-25_fmax-0-5	239	238	239	239	NaN	NaN	239

[1] D.C. Van Essen, K. Ugurbil, E. Auerbach, D. Barch, T.E.J. Behrens, R. Bucholz, A. Chang, L. Chen, M. Corbetta, S.W. Curtiss, S. Della Penna, D. Feinberg, M.F. Glasser, N. Harel, A.C. Heath, L. Larson-Prior, D. Marcus, G. Michalareas, S. Moeller, R. Oostenveld, S.E. Petersen, F. Prior, B.L. Schlaggar, S.M. Smith, A.Z. Snyder, J. Xu, and E. Yacoub. The Human Connectome Project: A data acquisition perspective. *NeuroImage*, 62(4):2222–2231, October 2012.

[2] David C Van Essen, Stephen M Smith, Deanna M Barch, Timothy EJ Behrens, Essa Yacoub, Kamil Ugurbil, Wu-Minn HCP Consortium, et al. The wu-minn human connectome project: an overview. *Neuroimage*, 80:62–79, 2013.

[3] Matthew F Glasser, Stephen M Smith, Daniel S Marcus, Jesper L R Andersson, Edward J Auerbach, Timothy E J Behrens, Timothy S Coalson, Michael P Harms, Mark Jenkinson, Steen Moeller, Emma C Robinson, Stamatios N Sotiropoulos, Junqian Xu, Essa Yacoub, Kamil Ugurbil, and David C Van Essen. The Human Connectome Project's neuroimaging approach. *Nature Neuroscience*, 19(9):1175–1187, September 2016.

[4] Matthew F Glasser, Stamatios N Sotiropoulos, J Anthony Wilson, Timothy S Coalson, Bruce Fischl, Jesper L Andersson, Junqian Xu, Saad Jbabdi, Matthew Webster, Jonathan R Polimeni, et al. The minimal preprocessing pipelines for the human connectome project. *Neuroimage*, 80:105–124, 2013.

[5] Lukas Snoek, Maite M. Van Der Miesen, Tinka Beemsterboer, Andries Van Der Leij, Annemarie Eigenhuis, and H. Steven Scholte. The Amsterdam Open MRI Collection, a set of multimodal MRI datasets for individual difference analyses. *Scientific Data*, 8(1):85, March 2021.

[6] Oscar Esteban, Christopher J. Markiewicz, Ross W. Blair, Craig A. Moodie, A. Ilkay Isik, Asier Erramuzpe, James D. Kent, Mathias Goncalves, Elizabeth DuPre, Madeleine Snyder, Hiroyuki Oya, Satrajit S. Ghosh, Jessey Wright, Joke Durnez, Russell A. Poldrack, and Krzysztof J. Gorgolewski. fMRIPrep: a robust preprocessing pipeline for functional MRI. *Nature Methods*, 16(1):111–116, January 2019.

[7] Evan M. Gordon, Timothy O. Laumann, Adrian W. Gilmore, Dillan J. Newbold, Deanna J. Greene, Jeffrey J. Berg, Mario Ortega, Catherine Hoyt-Drazen, Caterina Gratton, Haoxin Sun, Jacqueline M. Hampton, Rebecca S. Coalson, Annie L. Nguyen, Kathleen B. McDermott, Joshua S. Shimony, Abraham Z. Snyder, Bradley L. Schlaggar, Steven E. Petersen, Steven M. Nelson, and Nico U.F. Dosenbach. Precision Functional Mapping of Individual Human Brains. *Neuron*, 95(4):791–807.e7, August 2017.

[8] Natacha Mendes, Sabine Oligschläger, Mark E. Lauckner, Johannes Golchert, Julia M. Huntenburg, Marcel Falkiewicz, Melissa Ellamil, Sarah Krause, Blazej M. Baczkowski, Roberto Cozatl, Anastasia Osoianu, Deniz Kumral, Jared Pool, Laura Golz, Maria Dreyer, Philipp Haueis, Rebecca Jost, Yelyzaveta Kramarenko, Haakon Engen, Katharina Ohrnberger, Krzysztof J. Gorgolewski, Nicolas Farrugia, Anahit Babayan, Andrea Reiter, H. Lina Schaare, Janis Reinelt, Josefin Röbbig, Marie Uhlig, Miray Erbey, Michael Gaebler, Jonathan Smallwood, Arno Villringer, and Daniel S. Margulies. A functional connectome phenotyping dataset including cognitive state and personality measures. *Scientific Data*, 6(1):180307, February 2019.

[9] Anahit Babayan, Miray Erbey, Deniz Kumral, Janis D. Reinelt, Andrea M. F. Reiter, Josefin Röbbig, H. Lina Schaare, Marie Uhlig, Alfred Anwander, Pierre-Louis Bazin, Annette Horstmann, Leonie Lampe, Vadim V. Nikulin, Hadas Okon-Singer, Sven Preusser, André Pampel, Christiane S. Rohr, Julia Sacher, Angelika Thöne-Otto, Sabrina Trapp, Till Nierhaus, Denise Altmann, Katrin Arelin, Maria Blöchl, Edith Bongartz, Patric Breig, Elena Cesnaite, Sufang Chen, Roberto Cozatl, Saskia Czerwonatis, Gabriele Dambrauskaite, Maria Dreyer, Jessica Enders, Melina Engelhardt, Marie Michele Fischer, Norman Forschack, Johannes Golchert, Laura Golz, C. Alexandrina Guran, Susanna Hedrich, Nicole Hentschel, Daria I. Hoffmann, Julia M. Huntenburg, Rebecca Jost, Anna Kosatschek, Stella Kunzendorf, Hannah Lammers, Mark E. Lauckner, Keyvan Mahjoory, Ahmad S. Kanaan, Natacha Mendes, Ramona Menger, Enzo Morino, Karina Näthe, Jennifer Neubauer, Handan Noyan, Sabine Oligschläger, Patricia Panczyszyn-Trzewik, Dorothee Poehlchen, Nadine Putzke, Sabrina Roski, Marie-Catherine Schaller, Anja Schieferbein, Benito Schlaak, Robert Schmidt, Krzysztof J. Gorgolewski, Hanna Maria Schmidt, Anne Schrimpf, Sylvia Stasch, Maria Voss, Annett Wiedemann, Daniel S. Margulies, Michael Gaebler, and Arno Villringer. A mind-brain-body dataset of MRI, EEG, cognition, emotion, and peripheral physiology in young and old adults. *Scientific Data*, 6(1):180308, February 2019.

[10] Russell H. Tobe, Anna MacKay-Brandt, Ryan Lim, Melissa Kramer, Melissa M. Breland, Lucia Tu, Yiwen Tian, Kristin Dietz Trautman, Caixia Hu, Raj Sangoi, Lindsay Alexander, Vilma Gabbay, F. Xavier Castellanos, Bennett L. Leventhal, R. Cameron Craddock, Stanley J. Colcombe, Alexandre R. Franco, and Michael P. Milham. A longitudinal resource for studying connectome development and its psychiatric associations during childhood. *Scientific Data*, 9(1):300, June 2022.

[11] Kate Brody Nooner, Stanley J. Colcombe, Russell H. Tobe, Maarten Mennes, Melissa M. Benedict, Alexis L. Moreno, Laura J. Panek, Shaquanna Brown, Stephen T. Zavitz, Qingyang Li, Sharad Sikka, David Gutman, Saroja Bangaru, Rochelle Tziona Schlachter, Stephanie M. Kamiel, Ayesha R. Anwar, Caitlin M. Hinz, Michelle S. Kaplan, Anna B. Rachlin, Samantha Adelsberg, Brian Cheung, Ranjit Khanuja, Chaogan Yan, Cameron C. Craddock, Vincent Calhoun, William Courtney, Margaret King, Dylan Wood, Christine L. Cox, A. M. Clare Kelly, Adriana Di Martino, Eva Petkova, Philip T. Reiss, Nancy Duan, Dawn Thomsen, Bharat Biswal, Barbara Coffey, Matthew J. Hoptman, Daniel C. Javitt, Nunzio Pomara, John J. Sidtis, Harold S. Koplewicz, Francisco Xavier Castellanos, Bennett L. Leventhal, and Michael P. Milham. The NKI-Rockland Sample: A Model for Accelerating the Pace of Discovery Science in Psychiatry. *Frontiers in Neuroscience*, 6, 2012.

[12] G. Shafiei, N. B. Esper, M. S. Hoffmann, L. Ai, J. Clucas, S. Covitz, S. Giavasis, C. Lane, K. Mehta, T. M. Moore, T. Salo, R. T. Shinohara, T. M. Tapera, M. E. Calkins, S. Colcombe, C. Davatzikos, R. E. Gur, R. C. Gur, P. M. Pan, A. P. Jackowski, L. A. Rohde, N. Tottenham, X. N. Zuo, M. Cieslak, A. R. Franco, G. Kiar, G. A. Salum, M. P. Milham, and T. D. Satterthwaite. Reproducible brain charts: An open data resource for mapping the developing brain and mental health. `https://osf.io/er248`, 2024. Retrieved from https://osf.io/er248.

[13] B. T. Thomas Yeo, Fenna M. Krienen, Jorge Sepulcre, Mert R. Sabuncu, Danial Lashkari, Marisa Hollinshead, Joshua L. Roffman, Jordan W. Smoller, Lilla Zöllei, Jonathan R. Polimeni, Bruce Fischl, Hesheng Liu, and Randy L. Buckner. The organization of the human cerebral cortex estimated by intrinsic functional connectivity. *Journal of Neurophysiology*, 106(3):1125–1165, 2011.

[14] Graham L Baum, Zaixu Cui, David R Roalf, Rastko Ciric, Richard F Betzel, Bart Larsen, Matthew Cieslak, Philip A Cook, Cedric H Xia, Tyler M Moore, et al. Development of structure–function coupling in human brain networks during youth. *Proceedings of the National Academy of Sciences*, 117(1):771–778, 2020.

[15] Bertha Vázquez-Rodríguez, Laura E. Suárez, Ross D. Markello, Golia Shafiei, Casey Paquola, Patric Hagmann, Martijn P. van den Heuvel, Boris C. Bernhardt, R. Nathan Spreng, and Bratislav Misic. Gradients of structure–function tethering across neocortex. *Proceedings of the National Academy of Sciences*, 116(42):21219–21227, 2019.

[16] Zhen-Qi Liu, Golia Shafiei, Sylvain Baillet, and Bratislav Misic. Spatially heterogeneous structure-function coupling in haemodynamic and electromagnetic brain networks. *NeuroImage*, 278:120276, 2023.

[17] Tal Yarkoni, Russell A Poldrack, Thomas E Nichols, David C Van Essen, and Tor D Wager. Large-scale automated synthesis of human functional neuroimaging data. *Nature methods*, 8(8):665–670, 2011.

[18] Russell A Poldrack, Aniket Kittur, Donald Kalar, Eric Miller, Christian Seppa, Yolanda Gil, D Stott Parker, Fred W Sabb, and Robert M Bilder. The cognitive atlas: toward a knowledge foundation for cognitive neuroscience. *Frontiers in neuroinformatics*, 5:17, 2011.

[19] Justine Y Hansen, Ross D Markello, Jacob W Vogel, Jakob Seidlitz, Danilo Bzdok, and Bratislav Misic. Mapping gene transcription and neurocognition across human neocortex. *Nature Human Behaviour*, 5(9):1240–1250, 2021.

[20] Michael I. Demidenko and Russell A. Poldrack. PyReliMRI: An Open-source Python tool for Estimates of Reliability in MRI Data, September 2023.

[21] David Liljequist, Britt Elfving, and Kirsti Skavberg Roaldsen. Intraclass correlation–a discussion and demonstration of basic features. *PloS one*, 14(7):e0219854, 2019.
