# Supplementary material for: Benchmarking methods for mapping functional connectivity in the brain
Source: Nat Methods. 2025 Jun 6;22(7):1593–602. doi: 10.1038/s41592-025-02704-4 (PMC12240839; doi:10.1038/s41592-025-02704-4)
Supplement: Supplementary file 2 — Reporting Summary [file 41592_2025_2704_MOESM2_ESM.pdf]

nature portfolio

Corresponding author(s): Bratislav Misic

Last updated by author(s): Jan 17, 2025

Reporting Summary

Nature Portfolio wishes to improve the reproducibility of the work that we publish. This form provides structure for consistency and transparency in reporting. For further information on Nature Portfolio policies, see our Editorial Policies and the Editorial Policy Checklist.

Statistics

For all statistical analyses, confirm that the following items are present in the figure legend, table legend, main text, or Methods section.

n/a	Confirmed	
☐	☒	The exact sample size (*n*) for each experimental group/condition, given as a discrete number and unit of measurement
☐	☒	A statement on whether measurements were taken from distinct samples or whether the same sample was measured repeatedly
☐	☒	The statistical test(s) used AND whether they are one- or two-sided<br>*Only common tests should be described solely by name; describe more complex techniques in the Methods section.*
☐	☒	A description of all covariates tested
☐	☒	A description of any assumptions or corrections, such as tests of normality and adjustment for multiple comparisons
☐	☒	A full description of the statistical parameters including central tendency (e.g. means) or other basic estimates (e.g. regression coefficient) AND variation (e.g. standard deviation) or associated estimates of uncertainty (e.g. confidence intervals)
☐	☒	For null hypothesis testing, the test statistic (e.g. F, t, r) with confidence intervals, effect sizes, degrees of freedom and P value noted<br>*Give P values as exact values whenever suitable.*
☒	☐	For Bayesian analysis, information on the choice of priors and Markov chain Monte Carlo settings
☒	☐	For hierarchical and complex designs, identification of the appropriate level for tests and full reporting of outcomes
☐	☒	Estimates of effect sizes (e.g. Cohen's *d*, Pearson's *r*), indicating how they were calculated

Our web collection on statistics for biologists contains articles on many of the points above.

Software and code

Policy information about availability of computer code

Data collection	We used publicly available datasets and did not require software and code for data collection.
Data analysis	The code and scripts used for analysis are available at https://github.com/netneurolab/liu_fc-pyspi with a list of Python packages used. The pyspi package (v0.4.1, commit c19d06) used to calculate the pairwise statistics is available at https://github.com/DynamicsAndNeuralSystems/pyspi. A singularity container is available at https://osf.io/75je2/. We also used Brain Connectivity Toolbox (https://sites.google.com/site/bctnet, Version 2019-03-03), Brainconn (https://github.com/FIU-Neuro/brainconn, master branch at commit 8cd436), and netneurotools (https://github.com/netneurolab/netneurotools, v0.2.3). Additional software packages used in the preprocessing of brain annotations can be found in the original publications cited.

For manuscripts utilizing custom algorithms or software that are central to the research but not yet described in published literature, software must be made available to editors and reviewers. We strongly encourage code deposition in a community repository (e.g. GitHub). See the Nature Portfolio guidelines for submitting code & software for further information.

Data

Policy information about availability of data

All manuscripts must include a data availability statement. This statement should provide the following information, where applicable:

- Accession codes, unique identifiers, or web links for publicly available datasets
- A description of any restrictions on data availability
- For clinical datasets or third party data, please ensure that the statement adheres to our policy

The Human Connectome Project (HCP) data is available at https://db.humanconnectome.org/data/projects/HCP_1200. Multimodal neurophysiological networks (including the Neurosynth-derived cognitive similarity network) are available at https://github.com/netneurolab/hansen_many_networks. Behavioral phenotypes are available at https://github.com/yetianmed/subcortex. The raw pyspi outputs and the singularity container used for calculation are available at https://osf.io/75je2/.

Human research participants

Policy information about studies involving human research participants and Sex and Gender in Research.

Reporting on sex and gender	N/A (we used Human Connectome Project (HCP) Young Adult S1200 release)
Population characteristics	N/A (we used Human Connectome Project (HCP) Young Adult S1200 release)
Recruitment	N/A (we used Human Connectome Project (HCP) Young Adult S1200 release)
Ethics oversight	N/A (we used Human Connectome Project (HCP) Young Adult S1200 release)

Note that full information on the approval of the study protocol must also be provided in the manuscript.

Field-specific reporting

Please select the one below that is the best fit for your research. If you are not sure, read the appropriate sections before making your selection.

☒ Life sciences ☐ Behavioural & social sciences ☐ Ecological, evolutionary & environmental sciences

For a reference copy of the document with all sections, see nature.com/documents/nr-reporting-summary-flat.pdf

Life sciences study design

All studies must disclose on these points even when the disclosure is negative.

Sample size	No sample size calculation was performed. For HCP data, we chose 326 subjects without family relationship. Effect of sample size was tested in the Sensitivity analysis.
Data exclusions	No data was excluded.
Replication	The analysis was conducted and replicated with (1) Schaefer 100-node y-network atlas, (2) Desikan-Killiany atlas, (3) Schaefer 200-node 7-network atlas (4) global signal removal.
Randomization	No randomization was performed as this study does not include experimental groups.
Blinding	Blinding is not relevant to this study because it does not include experimental groups.

Reporting for specific materials, systems and methods

We require information from authors about some types of materials, experimental systems and methods used in many studies. Here, indicate whether each material, system or method listed is relevant to your study. If you are not sure if a list item applies to your research, read the appropriate section before selecting a response.

Materials & experimental systems

n/a	Involved in the study
☒	☐ Antibodies
☒	☐ Eukaryotic cell lines
☒	☐ Palaeontology and archaeology
☒	☐ Animals and other organisms
☒	☐ Clinical data
☒	☐ Dual use research of concern

Methods

n/a	Involved in the study
☒	☐ ChIP-seq
☒	☐ Flow cytometry
☐	☒ MRI-based neuroimaging

Magnetic resonance imaging

Experimental design

Design type: Resting-state fMRI

Design specifications: N/A (Resting-state fMRI; HCP Young Adult Protocols)

Behavioral performance measures: N/A (Resting-state fMRI; HCP Young Adult Protocols)

Acquisition

Imaging type(s): functional, structural, diffusion

Field strength: 3T

Sequence & imaging parameters: HCP Young Adult Protocols available at https://www.humanconnectome.org/hcp-protocols-ya-3t-imaging

Area of acquisition: Whole-brain

Diffusion MRI: ☒ Used ☐ Not used

Parameters: HCP Young Adult Protocols available at https://www.humanconnectome.org/hcp-protocols-ya-3t-imaging

Preprocessing

Preprocessing software: We used the pre-processed Human Connectome Project (HCP) Young Adult S1200 release openly available at https://www.humanconnectome.org/study/hcp-young-adult/document/1200-subjects-data-release.

Normalization: Image processing includes correcting for gradient distortion caused by non-linearities, correcting for bias field distortions, and registering the images to a standard reference space.

Normalization template: fs_LR_32k surface mesh

Noise and artifact removal: FMRIB's ICA-based X-noisefier (FIX)

Volume censoring: No volume censoring was performed.

Statistical modeling & inference

Model type and settings: Predictive models were used for brain-behavior prediction. We used kernel ridge regression with linear kernel for the main analysis and replicated with kernel ridge regression with cosine kernel, linear ridge regression, and LASSO regression.

Effect(s) tested: We tested whether pairwise interaction statistics predict the brain-behavior relationship.

Specify type of analysis: ☒ Whole brain ☐ ROI-based ☐ Both

Statistic type for inference (See Eklund et al. 2016): N/A

Correction: N/A

Models & analysis

n/a	Involved in the study
☐	☒ Functional and/or effective connectivity
☐	☒ Graph analysis
☐	☒ Multivariate modeling or predictive analysis

Functional and/or effective connectivity	We used pyspi package to calculate 239 pairwise interaction statistics. A complete list is attached with the manuscript.
Graph analysis	We used both subject- and group-level weighted functional connectivity derived from the 239 pairwise interaction statistics.
Multivariate modeling and predictive analysis	We used kernel ridge regression with linear kernel to predict behavioral phenotypes from functional connectivity in a nested 10-fold cross validation setting.

March 2021
